# Identification of optimal feature genes in patients with thyroid associated ophthalmopathy and their relationship with immune infiltration: a bioinformatics analysis

**DOI:** 10.3389/fendo.2023.1203120

**Published:** 2023-10-13

**Authors:** Chao Xiong, Yaohua Wang, Yue Li, Jinhai Yu, Sha Wu, Lili Wu, Boyuan Zhang, Yunxiu Chen, Puying Gan, Hongfei Liao

**Affiliations:** ^1^ Department of Ophthalmology, Affiliated Eye Hospital of Nanchang University, Nanchang, Jiangxi, China; ^2^ Jiangxi Clinical Research Center for Ophthalmic Disease, Nanchang, Jiangxi, China; ^3^ Jiangxi Research Institute of Ophthalmology and Visual Science, Nanchang, Jiangxi, China; ^4^ Jiangxi Provincial Key Laboratory for Ophthalmology, Nanchang, Jiangxi, China

**Keywords:** thyroid associated ophthalmopathy, optimal feature genes, pathogenesis, immune infiltration, GEO

## Abstract

**Background:**

Thyroid associated ophthalmopathy (TAO) is an organ-specific autoimmune disease that has a significant impact on individuals and society. The etiology of TAO is complicated and poorly understood. Thus, the goal of this study was to use bioinformatics to look into the pathogenesis of TAO and to identify the optimum feature genes (OFGs) and immune infiltration patterns of TAO.

**Methods:**

Firstly, the GSE58331 microarray data set was utilized to find 366 differentially expressed genes (DEGs). To find important modular genes, the dataset was evaluated using weighted gene coexpression network analysis (WGCNA). Then, the overlap genes of major module genes and DEGs were further assessed by applying three machine learning techniques to find the OFGs. The CIBERSORT approach was utilized to examine immune cell infiltration in normal and TAO samples, as well as the link between optimum characteristic genes and immune cells. Finally, the related pathways of the OFGs were predicted using single gene set enrichment analysis (ssGSEA).

**Results:**

KLB, TBC1D2B, LINC01140, SGCG, TMEM37, and LINC01697 were the six best feature genes that were employed to create a nomogram with high predictive performance. The immune cell infiltration investigation revealed that the development of TAO may include memory B cells, T cell follicular helper cells, resting NK cells, macrophages of type M0, macrophages of type M1, resting dendritic cells, active mast cells, and neutrophils. In addition, ssGSEA results found that these characteristic genes were closely associated with lipid metabolism pathways.

**Conclusion:**

In this research, we found that KLB, TBC1D2B, LINC01140, SGCG, TMEM37, and LINC01697 are intimately associated with the development and progression of TAO, as well as with lipid metabolism pathways.

## Introduction

Thyroid associated ophthalmopathy (TAO), also known as thyroid eye disease, Graves’ ophthalmopathy, or Graves’ orbitopathy, is a disabling and aesthetically unpleasing condition of the orbit that is typically linked to hyperthyroidism caused by Graves’ disease (1). The frequency of TAO ranges from 0.54 to 3.3 cases per 100,000 people per year, with females being more affected than males (2). TAO causes eyelid retraction, diplopia, exophthalmia, exposed keratitis, corneal opacity, and ulcers, as well as compression optic neuropathy, which affects eyesight in extreme instances (3). Patients with TAO have a higher prevalence of mild and non-progressive cases, with moderate to severe cases affecting only 5–6% of patients. Even mild TAO can have an impact on patients’ quality of life (QoL) and pose a significant threat to public health (4).

Most of the signs and symptoms of TAO are brought on by increased orbital bone volume pressure caused by orbital soft tissue dilation. The pathogenesis of TAO is complex and is believed to be mainly related to environmental, genetic and immune factors. Many factors that influence TAO, including race, sex, age, smoking history (5), radiation stimulant hormone (RAI) therapy (6), Hypercholesterolemia (7), Oxidative stress and thyroid-stimulating hormone receptor antibodies, have been confirmed by studies (1, 8). It is currently believed that pathological autoimmune responses are targeted at cross-reacting autoantigens in the thyroid and retrobulbar tissues (3, 9). Cytokines and immune mechanisms are considered to play a major role in the pathogenesis of TAO. However, the underlying mechanism of TAO is still unknown, and existing therapies can only slow the disease’s development. Therefore, further studies on the pathogenesis and potential therapeutic targets of TAO are urgently needed.

In this study, bioinformatics analysis, WGCNA and three machine learning methods were used to screen and identify best feature genes of TAO. For the purpose of further understanding the molecular immune mechanism during the development of TAO, the CIBERSORT method was then applied to systematically analyze the infiltrating fraction of 22 different types of immune cells in TAO and normal specimens. This analysis also examined the association between OFGs and infiltrating immune cells. Finally, ssGSEA was performed to predict the possible related pathways of OFGs.

## Materials & methods

### Data collecting and analyzing

We used the keyword graves ophthalmopathy to filter out the GSE58331 microarray, which contained data on TAO anterior orbit tissue and normal anterior orbit tissue, from the Gene Expression Omnibus database. The GSE58331 dataset included 175 samples (anterior orbital tissue or lacrimal gland tissue), 29 of which were from healthy individuals and the remainder from patients with inflammatory diseases such as NSOI, sarcoidosis, GPA, and TAO. The collection contained 35 TAO samples, comprising 27 samples of anterior orbital tissue and 8 samples of lacrimal glands. Forty-nine samples from GSE58331 were selected, including 27 anterior orbital tissue samples from patients with TAO and 22 anterior orbital tissue samples from normal subjects.

### Differentially expressed gene analysis and WGCNA

The R (version 4.2.0) limma package was applied for examining differentially expressed genes (DEGs) from datasets. Statistical differences were defined as a P value <0.05 and a fold change (FC) ≥1.5 (|log2FC| ≥0.584963). Due to the interconnection of gene sets and the relationship between gene sets and phenotypes, WGCNA was used to find highly synergistic gene sets and identify biomarkers (10). The module’s minimum number of genes is set to 30, and the “WGCNA” R package (11) was employed to build a coexpression network for the GSE58331. Correlation analysis was used to identify the crucial modules, and the hub genes were chosen from the genes in those modules.

### DO, GO and KEGG enrichment analyses

The intersection of DEGs with hub genes is referred to as key genes. Using the “clusterProfile”, “enrichplot”, “ggplot2”, “org.Hs.eg.db”, “GOplot”, and “DOSE” packages in R, we conducted a disease ontology (DO), gene ontology (GO), and Kyoto Encyclopedia of Genes and Genomes (KEGG) study to ascertain the function of the key genes.

### Screening of OFGs

Three machine learning methods were utilized to select OFGs in our study. To determine the optimal penalty value with the least binomial deviation, the R package “glmnet” was utilized for least absolute shrinkage and selection operator (LASSO) (12) logistic regression analysis. The R packages “e1071”, “kernlab”, and “caret” were used to carry out the support vector machine recursive feature elimination (SVM-RFE) (13) method, and the result with the minimum cross validation error was studied. The RF method to determining the level with the least amount of error was developed by the R package “randomForest”. By intersecting the outcomes of these three machine learning algorithms, the final OFGs will be obtained.

### Validation of OFGs

The diagnostic significance of OFGs in TAO was assessed using receiver operating characteristic (ROC) curves created in R by using the “pROC” tool. The area under the ROC curve (AUC) showed the diagnostic effectiveness of OFGs.

### Infiltrating immune cell evaluation and correlation analysis

A violin diagram was used to visually depict the differences in immune cells between TAO and control samples. The R software was employed to examine the relationships between OFGs and infiltrating immune cells.

### Single-gene gene set enrichment analysis

To ascertain the relative enrichment of each HALLMARK pathway in the samples, ssGSEA was performed in R using the “GSVA” package. Finally, the correlation analysis between OFGs and pathway was carried out.

## Results

### Screening of DEGs

**Figure 1:**
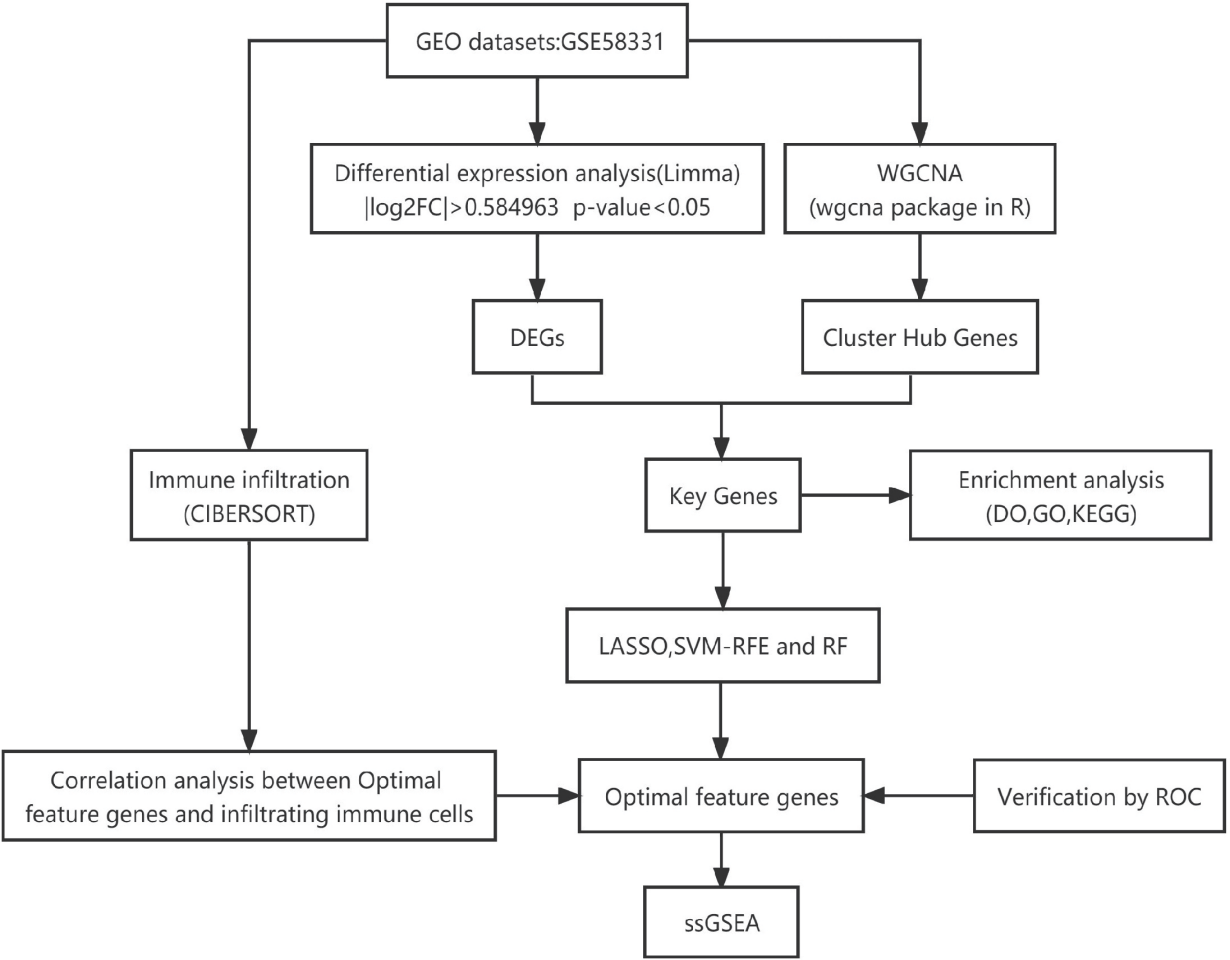
The flow chart of this investigation.

**Figure 2:**
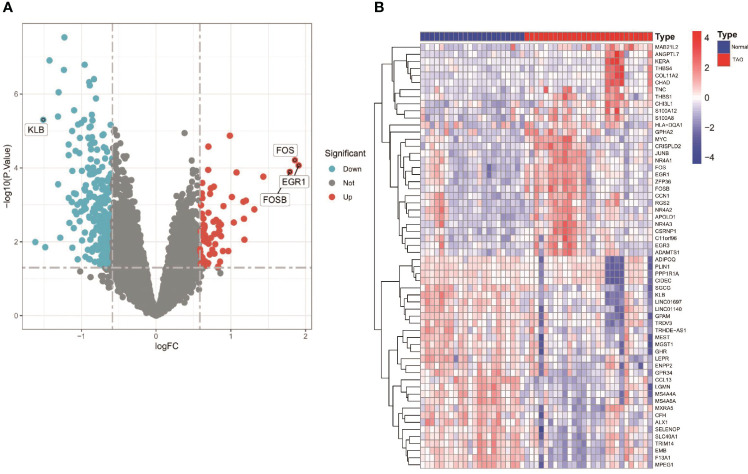
Differentially expressed genes (DEGS) in orbital adipose tissue between the TAO and Normal individuals. **(A)** Volcano plot of DEGs. Data points in red are up-regulated genes, and in blue are down-regulated genes. The top up-regulated and down-regulated genes are shown. **(B)** A Heatmap of the top 30 up-regulated DEGs and top 30 down-regulated DEGs are shown.

**Figure 1** shows an overview of the present study. We performed differential gene expression analysis to investigate gene expression in TAO patients and normal samples. A total of 366 DEGs were screened from GSE58331 data set, including 69 upregulated and 297 downregulated genes (**Figures 2A, B**).

### Screening of target modules and genes based on WGCNA

**Figure 3:**
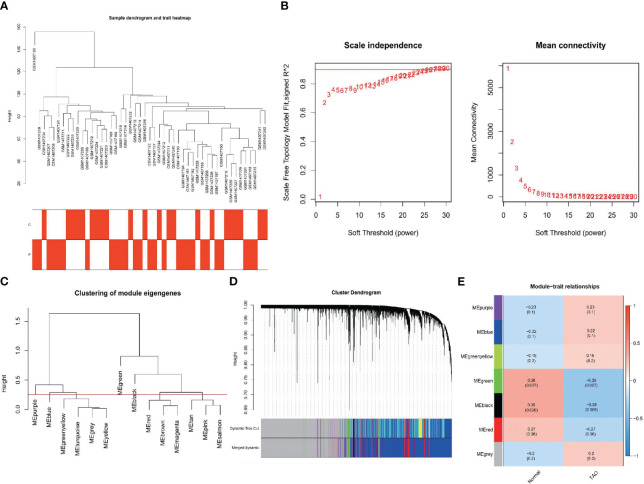
Construction of WGCNA networks. **(A)** Sample dendrogram and trait heatmap. The two traits are TAO and normal. **(B)** Scale independence and mean connectivity of various soft-thresholding values. **(C)** Clustering of module eigengenes. **(D)** Gene dendrogram and modules color. **(E)** Module-trait relationships.

As shown in **Figure 3A**, clustering analysis of all samples showed that the GSM1407185 sample was poorly clustered. Therefore, this sample was excluded as an outlier in the WGCNA analysis. When β=26, the scale-free distribution and gene connections were most consistent, according to the study of soft threshold selection (**Figure 3B**). By combining modules with feature factors greater than 0.75 and limiting the smallest number of genes in the module to 30 in the weighted gene coexpression network, seven coexpression modules were examined further (**Figure 3C**). MEgreen and MEblack are the two modules with the strongest correlation, according to our investigation into module correlation (**Figure 3D**). For additional investigation, 483 genes were chosen from the two modules.

### Functional enrichment analyses

**Figure 4:**
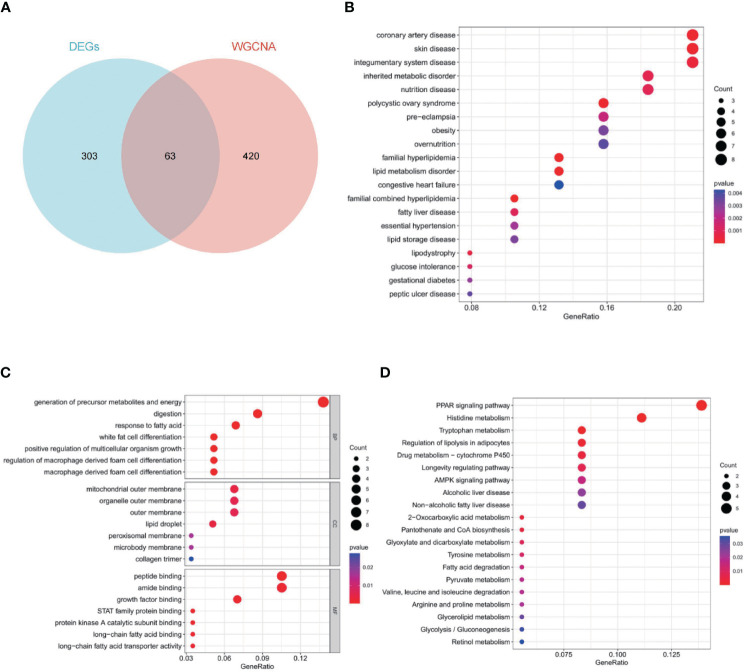
Sixty-three key genes and their functional analysis. The bubble shows significant items according to the P-value. **(A)** Venn diagram demonstrated the intersection set of WGCNA and EDGs. **(B)** Disease Ontology (DO) enrichment analysis. **(C)** Gene Ontology (GO) enrichment analysis. BP, biological processes; CC, cellular components; MF, molecular functions. **(D)** Gene Set Enrichment Analysis (GSEA).

To obtain 63 key genes, the intersection of the EDGs and the WGCNA key module genes was employed (**Figure 4A**). To further understand the roles of those genes, function enrichment analysis was performed. Disease ontology (DO) enrichment analysis showed that it was mainly concentrated in coronary artery disease, skin disease, integumentary system disease, inherited metabolic disorder and nutrition disease (**Figure 4B**). For biological process (BP), mainly related to generation of precursor metabolites and energy, digestion, response to fatty acid and white fat cell differentiation. For cellular component (CC), it mainly contains mitochondrial outer membrane, organelle outer membrane, outer membrane and lipid droplet. Peptide binding, amide binding, growth factor binding, and STAT family protein binding were highly enriched for molecular function (MF) (**Figure 4C**). The key genes were predominantly abundant in the PPAR signaling pathway, histidine metabolism, tryptophan metabolism, regulation of lipolysis in adipocytes, and drug metabolism-cytochrome P450, according to the results of KEGG pathway analysis (**Figure 4D**).

### Screening and verification of optimal feature genes

**Figure 5:**
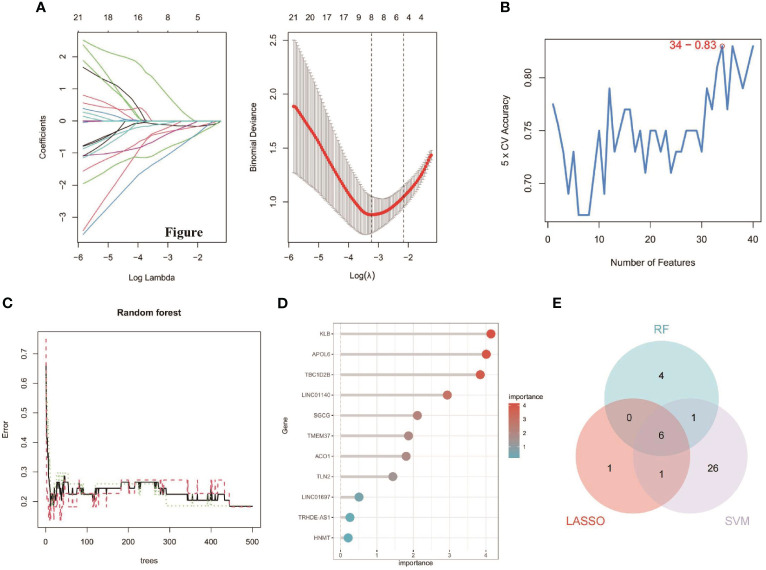
Detection of diagnostic biomarkers for TAO. **(A)** Biomarker detection using LASSO regression analysis. **(B)** biomarker detection by SVM-REF. **(C, D)** Biomarker detection by random forest. **(E)** Venn diagram shows the shared diagnostic markers between LASSO, SVM-REF, and random forest.

**Figure 6:**
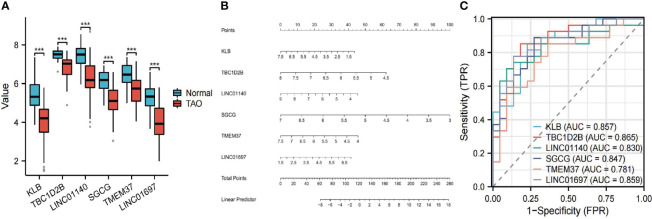
**(A)** The expression difference of the six genes between TAO and Normal. ***, P < 0.001. **(B)** Based on the six genes, a nomogram was constructed for TAO. **(C)** The predictive value of the six genes in TAO from the ROC curve. Each panel displayed the AUC under the curve and 95% CI.

The 63 key genes were employed in LASSO, SVM-RFE, and RF analyses to select the OFGs in TAO. The LASSO logistic regression approach was used in this work to discover 8 major biomarkers from key genes (**Figure 5A**). The SVM-RFE method identified 34 genes as important biomarkers (**Figure 5B**). Furthermore, using the RF technique, 11 genes were identified as critical biomarkers (**Figures 5C, D**). KLB, TBC1D2B, LINC01140, SGCG, TMEM37 and LINC01697 were overlapping genes in the three algorithms (**Figure 5E**), and these 6 genes were down-regulated genes (**Figure 6A**). The predictive nomogram was produced using a scoring method in which the relative expression of each gene was given a value, and the sum of all the gene scores was used to get the overall score. The nomogram’s ROC analysis produced an AUC of 0.860, indicating a strong predictive value (**Figure 6B**). With AUCs of 0.857, 0.865, 0.830, 0.847, 0.781, and 0.859, respectively, the ROC curves of KLB, TBC1D2B, LINC01140, SGCG, TMEM37, and LINC01697 demonstrated their likelihood as important biomarkers (**Figure 6C**), demonstrating that the six biological indicators exhibited high prediction accuracy.

### Infiltration of immune cells results

**Figure 7:**
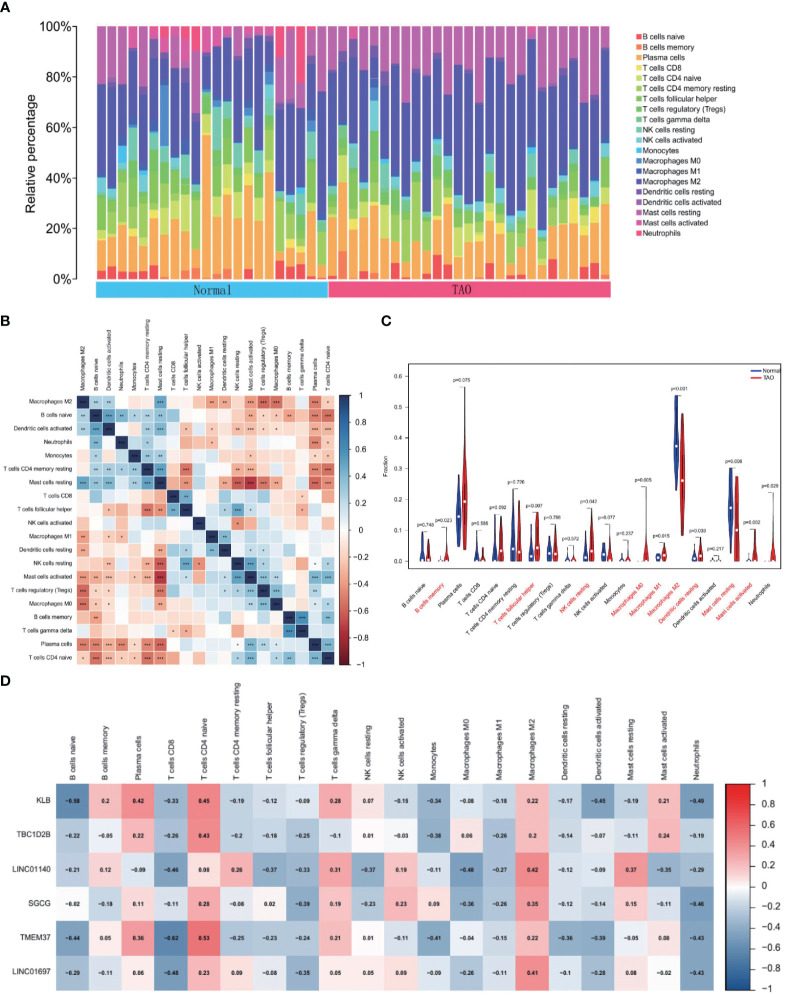
Immune cell infiltration analysis. **(A)** The bar plot shows the proportion of immune cells in different samples. **(B)** Heatmap of correlation in 22 types of immune cells. Blue represents a positive correlation, and red represents a negative correlation. Darker color implies stronger association. *P < 0.05; **P < 0.01; ***P < 0.001. **(C)** Violin diagram of the proportion of 22 types of immune cells. The red marks represent the difference in infiltration between the TAO and Normal samples. **(D)** Correlation analysis of immune cell infiltrations with six optimal feature genes. Red represents a positive correlation, and blue represents a negative correlation. Darker color implies stronger correlation.

To find variations in immune infiltration between normal and TAO specimens, CIBERSORT was used (**Figures 7A, B**). As indicated from the correlation heatmap of the 22 types of immune cells, activated mast cells and resting NK cells, activated dendritic cells and naive B cells, T cells gamma delta and memory B cells and resting mast cells and resting T cells CD4 memory displayed a significant positive correlation, respectively. T cells regulatory (Tregs) and M2 macrophages, Tregs and resting mast cells, M0 macrophages and M2 macrophages, naive T cells CD4 and naive B cells, naive T cells CD4 and resting T cells CD4 memory, resting NK cells and resting mast cells, activated mast cells and resting mast cells, plasma cells and resting mast cells and T cells follicular helper and resting T cells CD4 memory displayed significant negative correlations, respectively. TAO samples generally contained more memory B cells, T follicular helper cells, resting NK cells, M0 macrophages, M1 macrophages, resting dendritic cells, activated mast cells, and neutrophils compared to normal samples. However, the proportions of M2 macrophages and resting mast cells were relatively lower (**Figure 7C**). Correlation heat map (**Figure 7D**) showing correlation analysis of 6 characteristic genes with immunoinfiltrating cells. KLB revealed a positive association with plasma cells and naive T cells CD4 and a negative correlation with naive B cells, activated dendritic cells, and neutrophils based on the findings of correlation analysis. TBC1D2B and naive T cells CD4 displayed a favorable connection. M2 macrophages and LINC01140 exhibited a positive association, whereas T cells CD8 and M0 macrophages and LINC01140 showed a negative correlation. Neutrophils showed a negative connection with SGCG. T cells CD8, naive B cells, monocytes, and neutrophils all showed a substantial negative connection with TMEM37, but naive T cells CD4 showed a positive correlation. The findings also revealed that LINC01697 substantially linked favorably with M2 macrophages and negatively with T cell CD8 and neutrophils.

### ssGSEA

**Figure 8:**
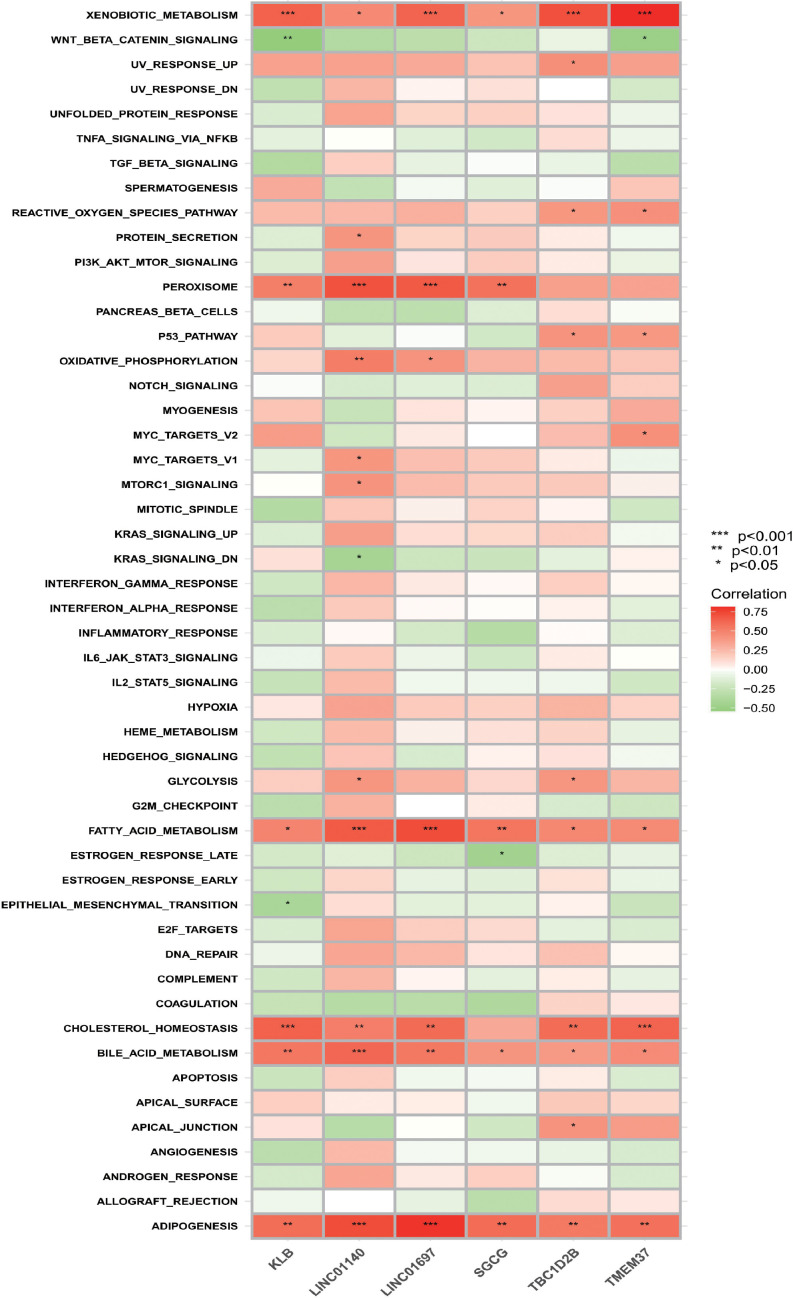
Correlation between OFGs and gene sets. Red represents a positive correlation, whereas green represents a negative correlation. The darker the color, the higher the correlation.

According to the results of ssGSEA (**Figure 8**), it is not difficult to find that the six OFGs are positively correlated with heterogeneous biological metabolism, fatty acid metabolism, bile acid metabolism and fat formation to varying degrees. All OFGs except SGCG were positively correlated with cholesterol homeostasis. In addition, KLB, LINC01140, LINC01697 and SGCG were positively correlated with the peroxisome gene set.

## Discussion

Thyroid associated eye disease (TAO) is an autoimmune thyroid associated orbital disease occurring in the eye and retrobulbar tissues, and has the highest incidence among adult orbital diseases. When the symptoms are severe, the appearance will be significantly affected and the vision of the patient will be endangered. At this time, the treatment effect of drugs and surgery is poor (2). The pathogenesis of TAO is complex, with the main processes including cytokine production, inflammation, hyaluronic acid synthesis, adipogenesis, and myofibrillogenesis (14). Understanding the molecular mechanism of TAO is very important for early diagnosis and precise treatment. Therefore, the discovery of characteristic biomarkers is crucial.

In our research, 63 key genes in TAO were evaluated using DEG and WGCNA, followed by DO, GO and KEGG pathway enrichment analyses. Intriguingly, DO pathway enrichment analyses showed that the majority of DEG-enriched diseases were linked to coronary heart disease, skin disease, integumentary system disease and familial hyperlipidemia, highlighting their potential roles in the development of TAO. Based on GO and KEGG enrichment studies, we preliminarily found that these genes are closely correlated with biological processes such as adipose differentiation and metabolism (response to fatty acid, white fat cell differentiation) and their related pathways (PPAR signaling pathway, regulation of adipocyte lipolysis, fatty acid degradation). Thereafter, three machine learning algorithms were used to pick the best feature genes connected to TAO. Ultimately, ROC curves and a nomogram were created to determine the OFGs’ diagnostic value. Six genes (KLB, TBC1D2B, LINC01140, SGCG, TMEM37, and LINC01697) were eventually found to have superior performance in discriminating between TAO and normal samples.

The βKlotho protein, encoded by the KLB gene, was first discovered in 2000 (15). In order to act as the physiological receptors for FGF21 and FGF19, respectively, βKlotho forms binary complexes with FGFR1c, which is expressed by adipocytes, and FGFR4, which is expressed by hepatocytes (16, 17). The liver and adipose tissue generate fibroblast growth factor-21 (FGF-21), a protein that belongs to the FGF family (18). Numerous types of physiological stress, such as hunger and inflammation, cause an increase in FGF21 levels in the blood. FGF21 interacts with the βKlotho-FGFR1c complex, causing a change in catabolic metabolism (19). Only a few tissues express βKlotho, such as the liver and both white and brown adipose tissue (20). In contrast to healthy control samples, our research found that the anterior orbital tissue of TAO patients had significantly lower levels of the KLB gene, which codes for the protein βKlotho. These results were inconsistent with the high expression of βKlotho in white adipose tissue. The significant downregulation of KLB gene in TAO orbital adipose tissue will reduce the FGF21-induced fat catabolism, which may be one of the reasons for increased orbital adipose tissue in patients with hyperthyroidism.

The TBC1D2B gene, which codes for a GTPase-activating protein involved in membrane trafficking, interacts with the early endosomal marker protein RAB5. The Rab family of small GTPases regulates internal membrane traffic by promoting the production, transport, and fusion of membrane-bound organelles and vesicles in eukaryotic cells (21). The expression of E-cadherin, which is essential for maintaining cell-cell interactions in physiology, is reduced during the epithelial-to-mesenchymal transition (EMT), which is a significant event (22). E-cadherin endocytosis has been demonstrated to be regulated by TBC1D2B, and its internalization and degradation were enhanced when TBC1D2B was downregulated (23). we hypothesize that TBC1D2B deletion may accelerate E-cadherin degradation, lead to EMT abnormalities in the anterior orbital tissue, epithelial cell differentiation into fibroblasts, and promote the development of fibrosis.

The long intergene non-coding RNAs LINC01140 and LINC01697 are able to cis-regulate transcription of nearby protein-coding genes and trans-regulate transcription of distal protein-coding genes (24). Low levels of LINC01140 have been linked to poor overall survival, poor disease-specific survival, and poor disease-free survival, according to research by Hu et al. (25) in metastatic sarcomas. Li et al.’s study (26), which found that LINC01140 expression was dramatically downregulated in breast cancer, suggests that it may be used as a biomarker to gauge a patient’s prognosis. Additionally, LINC01697 has been identified as a possible prognostic biomarker for gastric cancer (27, 28)and oral squamous cell carcinoma (29, 30). According to recent research, the co-expression relationship of differentially expressed lncRNA and extracellular matrix related mRNA implies that lncRNA may be involved in the control of extracellular matrix remodeling in TAO orbital fat/connective tissue (31). Furthermore, the lncRNA-miRNA-mRNA network was built using high-throughput sequencing of orbital tissue, which was likewise linked to the etiology of TAO (32). LINC01140 has been shown in cell tests to control macrophage M2 polarization (33) and lessen their inflammatory response (34). However, no investigations on how LINC01697 operates in non-tumor cells have been published.

Sarcolemmal transmembrane glycoprotein (SGCG) is a component of the sarcoglycan complex, which connects the extracellular matrix with the F-actin cytoskeleton in muscle cells (35). Skeletal muscle fibrosis frequently occurs due to an SGCG deficiency-related vulnerability to muscle injury. After repeated cycles of degeneration, collagen and other extracellular matrix elements replace the muscle tissue, which causes the development of scar tissue and accelerates the course of the illness (36). TMEM37 is a transmembrane protein (37) involved in the transmembrane transport of calcium ions and the regulation of ion transmembrane transport. At present, there are few studies on transmembrane protein TMEM37, and its more detailed biological function is not clear. Six OFGs (KLB, TBC1D2B, LINC01140, SGCG, TMEM37, and LINC01697) are expected to affect TAO progression and serve as diagnosis indicators in light of the findings discussed above. Nevertheless, a significant number of clinically relevant articles are still needed to confirm their diagnostic value.

The current study used CIBERSORT to evaluate the immune infiltrating process within TAO in order to more precisely explore the impacts produced by immune cell infiltration. The infiltration of memory B cells, T cells follicular helper, resting NK cells, M0 Macrophages, M1 Macrophages, resting dendritic cells, activated Mast cells and Neutrophils increased, while the infiltration of M2 Macrophages and resting Mast cells decreased, probably showing associations with TAO occurrence and progresses. Moreover, we observed that the six genes were associated with many immunocytes. T follicular helper (Tfh) cells are identified as a unique T-cell subset that aids in germinal center formation, B-cell growth and affinity maturation, and immunoglobulin class flipping as an essential component of adaptive immunity (38). Tfh cells feature the characteristic transcription factor B-cell lymphoma 6, distinct surface markers such as chemokine receptor 5, T-cell costimulatory factors, and programmed cell death 1 and distinct cytokine expression patterns (such as IL-21, IL-4, and IL-10). Serum IL-21 levels significantly increased in Graves’ disease (GD) patients, decreased following therapy, and were positively correlated with the severity of GD, according to zhang et al.’s (39) research. In addition, thyroid tissues from GD patients showed higher numbers of Tfh cells and markers associated with them (CLCX13 and CXCR5) (40). Knowing how Tfh cells and IL-21 are involved in the immune system may help develop treatment strategies for autoimmune illnesses due to their active roles in the immune system. At present, there are few studies on Tfh cells in the pathogenesis of TAO, which can be further studied in the future. Depending on their various phenotypes, macrophages mediate various impacts on immunometabolism. When M1 macrophages are stimulated by lipopolysaccharide and interferon, they release tumor necrosis factor, IL-1, IL-6, and other proinflammatory cytokines, whereas M2 macrophages are stimulated by IL-4 and IL-13, and their anti-inflammatory cytokines include IL-10 and arginase-1 (41). Therefore, it is not difficult to understand that the M1 macrophages in the anterior orbital tissue of TAO are increased and the M2 macrophages are decreased compared with the normal subjects, and these OFGs (especially LINC01140 and LINC01697) have a negative association with M1 macrophages and a positive association with M2 macrophages to varying degrees. Previous studies (42, 43) have found an increased number of mast cells near fibroblasts in TAO orbital tissue, suggesting that mast cells are involved in the activation of orbital fibroblasts. Mast cells are thus thought to be orbital fibroblast activity regulators in TAO, and they may be therapeutic targets for TAO (44). Furthermore, we discovered that immune cell infiltration and OFGs had a negative connection with neutrophils. This finding may suggest that downregulating OFGs facilitates neutrophil infiltration in the anterior orbital region.

In the final results of ssGSEA, it was easy to find that the selected characteristic genes were closely related to lipid metabolism-related pathways, which also suggested that lipid metabolism was associated with the occurrence and progression of TAO.

Our research adds to our understanding of TAO pathogenesis, although it has certain limitations. First, our team only collected data from GEO databases, and sample size was small. More research studies with larger sample sizes are required to confirm the conclusions presented here. Second, while six genes have been identified as prospective TAO biomarkers, no *in vivo* or *in vitro* investigations have been undertaken, thus more research is needed to investigate the function and probable processes of these genes in the illness.

## Conclusion

In this research, we found that KLB, TBC1D2B, LINC01140, SGCG, TMEM37 and LINC01697 are intimately associated with lipid metabolism pathways and the pathogenesis and progression of TAO.

## Data availability statement

Publicly available datasets were analyzed in this study. This data can be found here: the GEO (https://www.ncbi.nlm.nih.gov/geo) database.

## Author contributions

CX and YW: As the first author of the study, performed all the data analysis and wrote the manuscript. YL, JY SW, LW, BZ, YC and PG: Responsible for collating research results and editing pictures. HL: As the instructor of this study, participated in the whole process of the study. All authors contributed to the article and approved the submitted version.
