# Supplementary figures and images for: Identification of optimal feature genes in patients with thyroid associated ophthalmopathy and their relationship with immune infiltration: a bioinformatics analysis
Source: Front Endocrinol (Lausanne). 2023 Oct 13;14:1203120. doi: 10.3389/fendo.2023.1203120 (PMC10611488; doi:10.3389/fendo.2023.1203120)

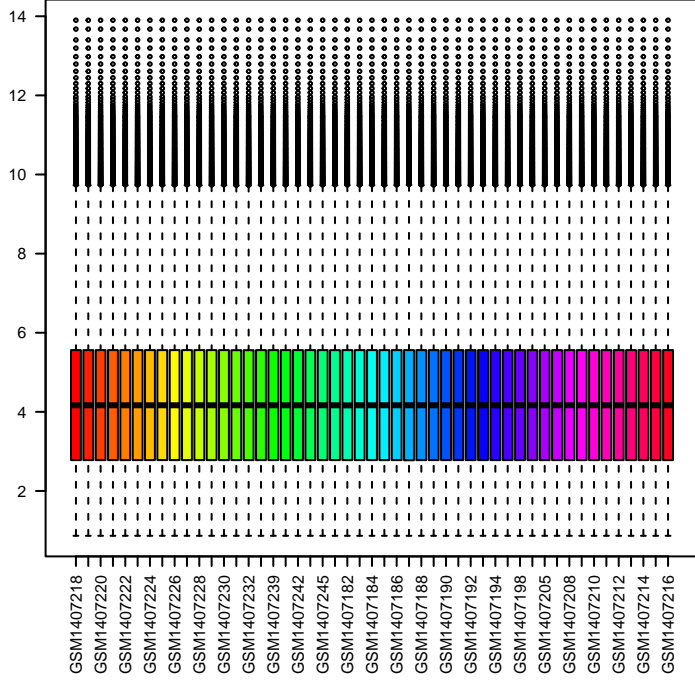

Supplement: Supplementary file 1 [file DataSheet_1.zip › 1.deg+wgcna/1.nor.pdf]

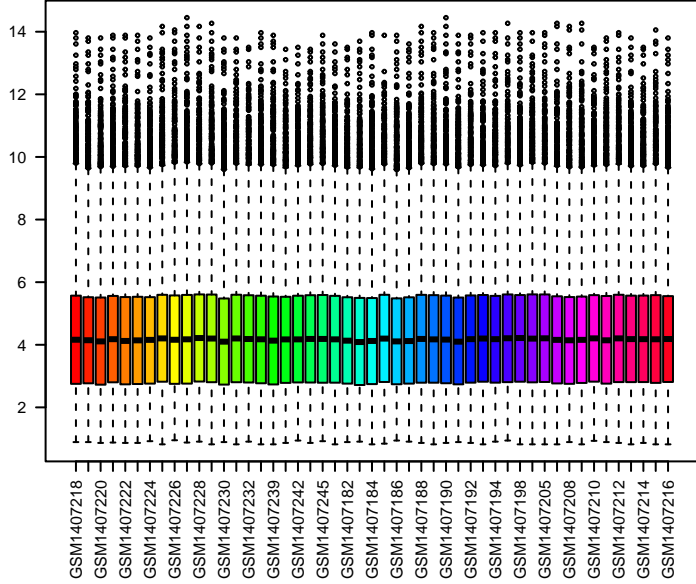

Supplement: Supplementary file 1 [file DataSheet_1.zip › 1.deg+wgcna/1.raw.pdf]

Network heatmap plot, selected genes

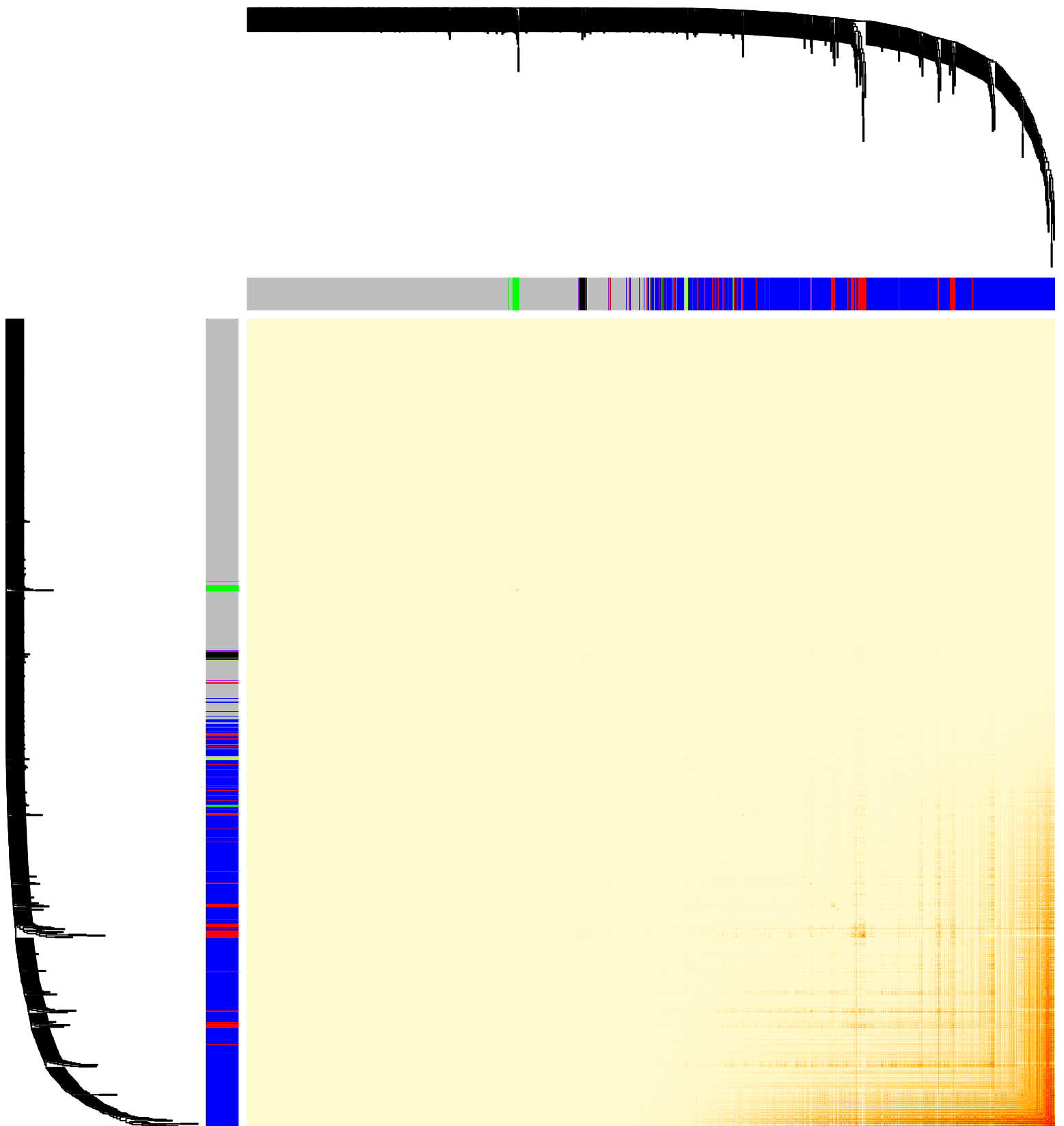

Supplement: Supplementary file 1 [file DataSheet_1.zip › 1.deg+wgcna/13_Network heatmap plot_selected genes.pdf]

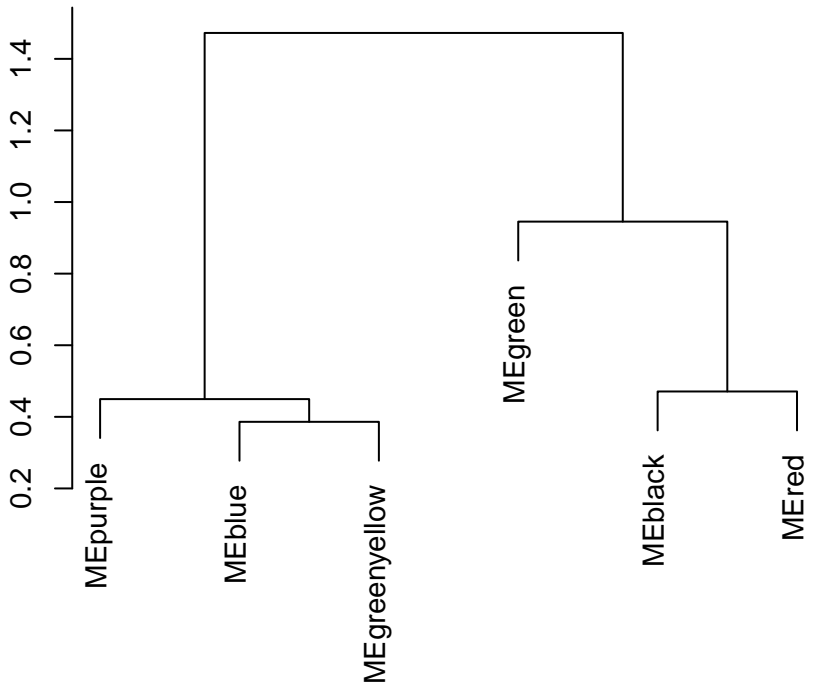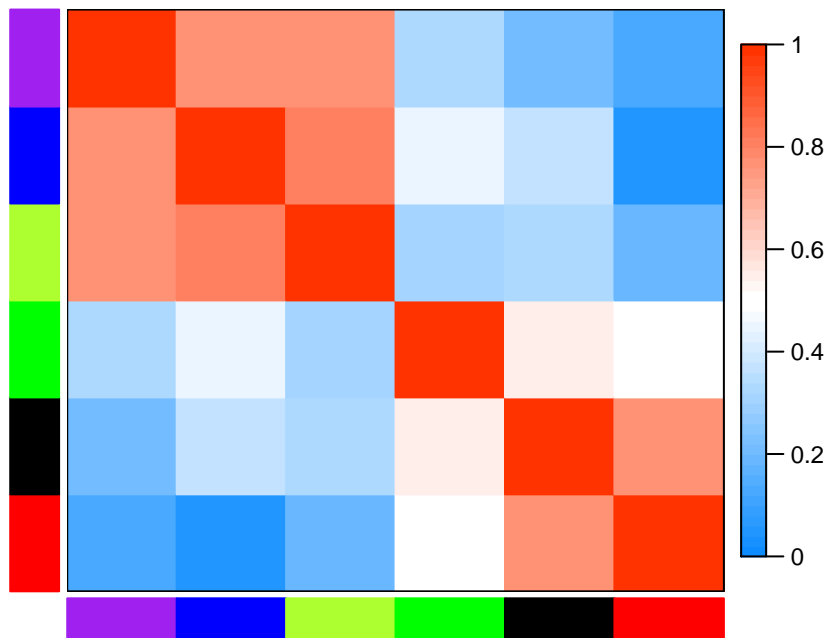

Supplement: Supplementary file 1 [file DataSheet_1.zip › 1.deg+wgcna/14_Eigengene dendrogram and Eigengene adjacency heatmap.pdf]

Sample clustering to detect outliers

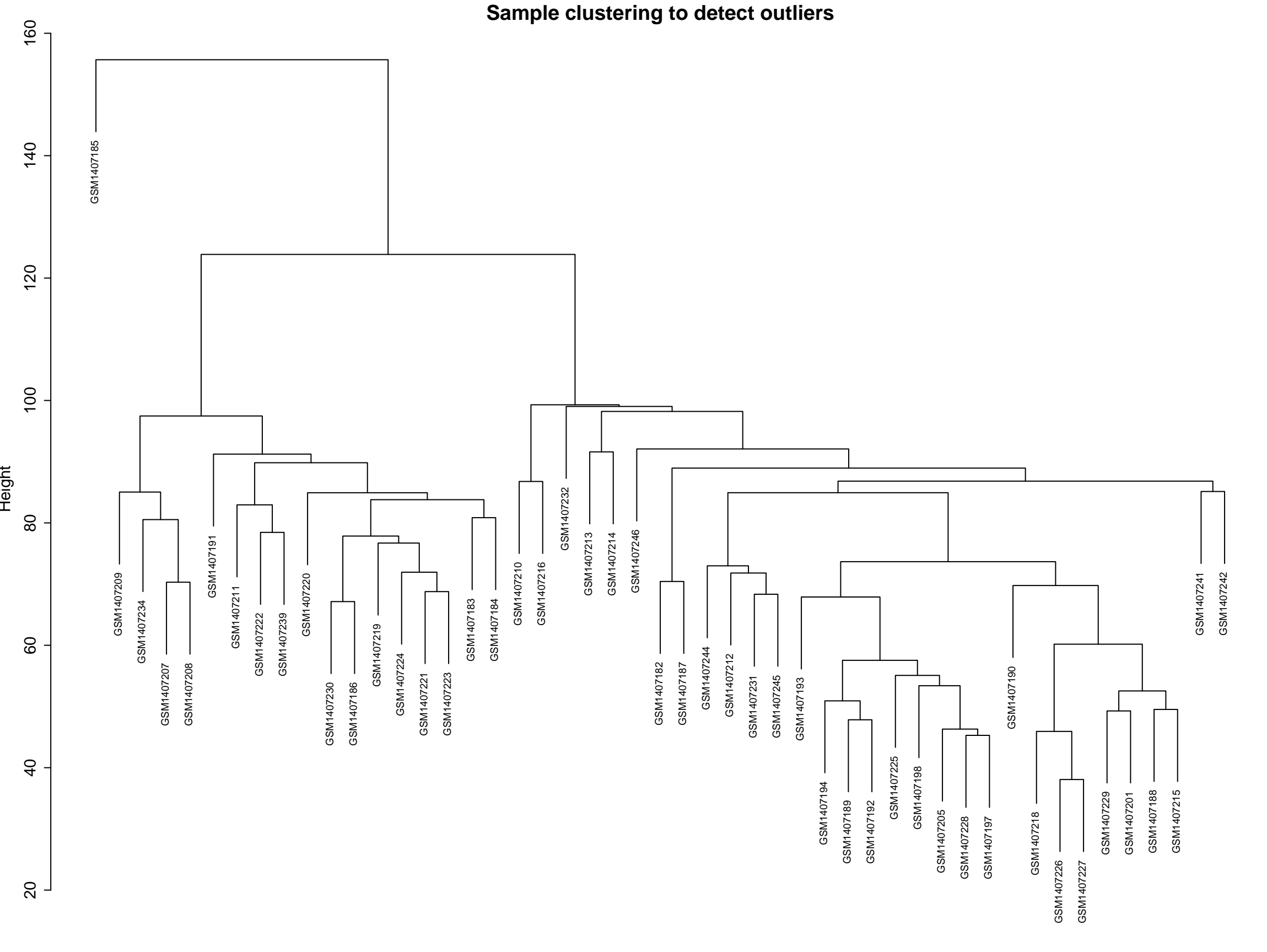

Supplement: Supplementary file 1 [file DataSheet_1.zip › 1.deg+wgcna/1_sampleClustering.pdf]

Sample dendrogram and trait heatmap

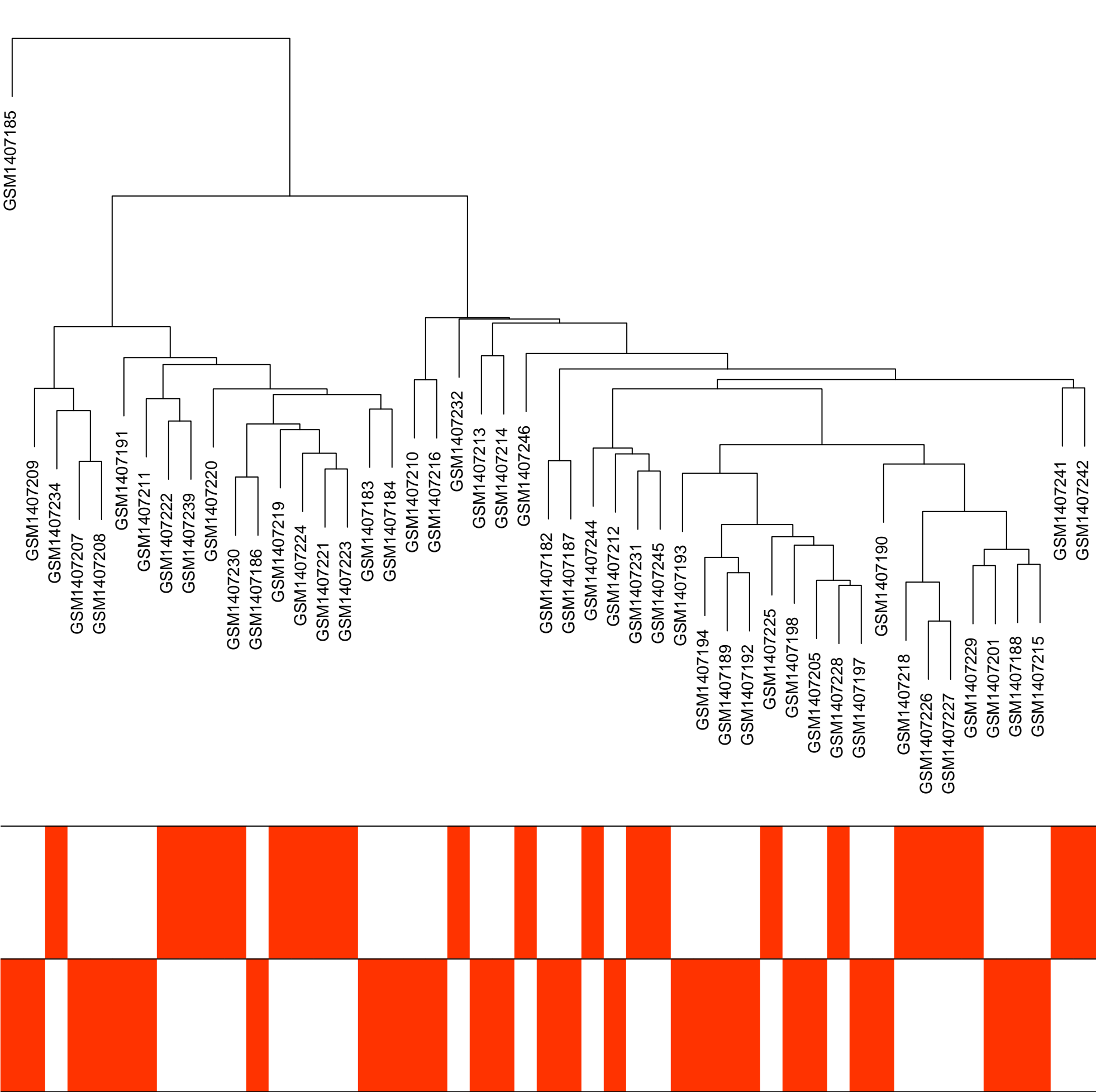

Supplement: Supplementary file 1 [file DataSheet_1.zip › 1.deg+wgcna/2_Sample dendrogram and trait heatmap.pdf]

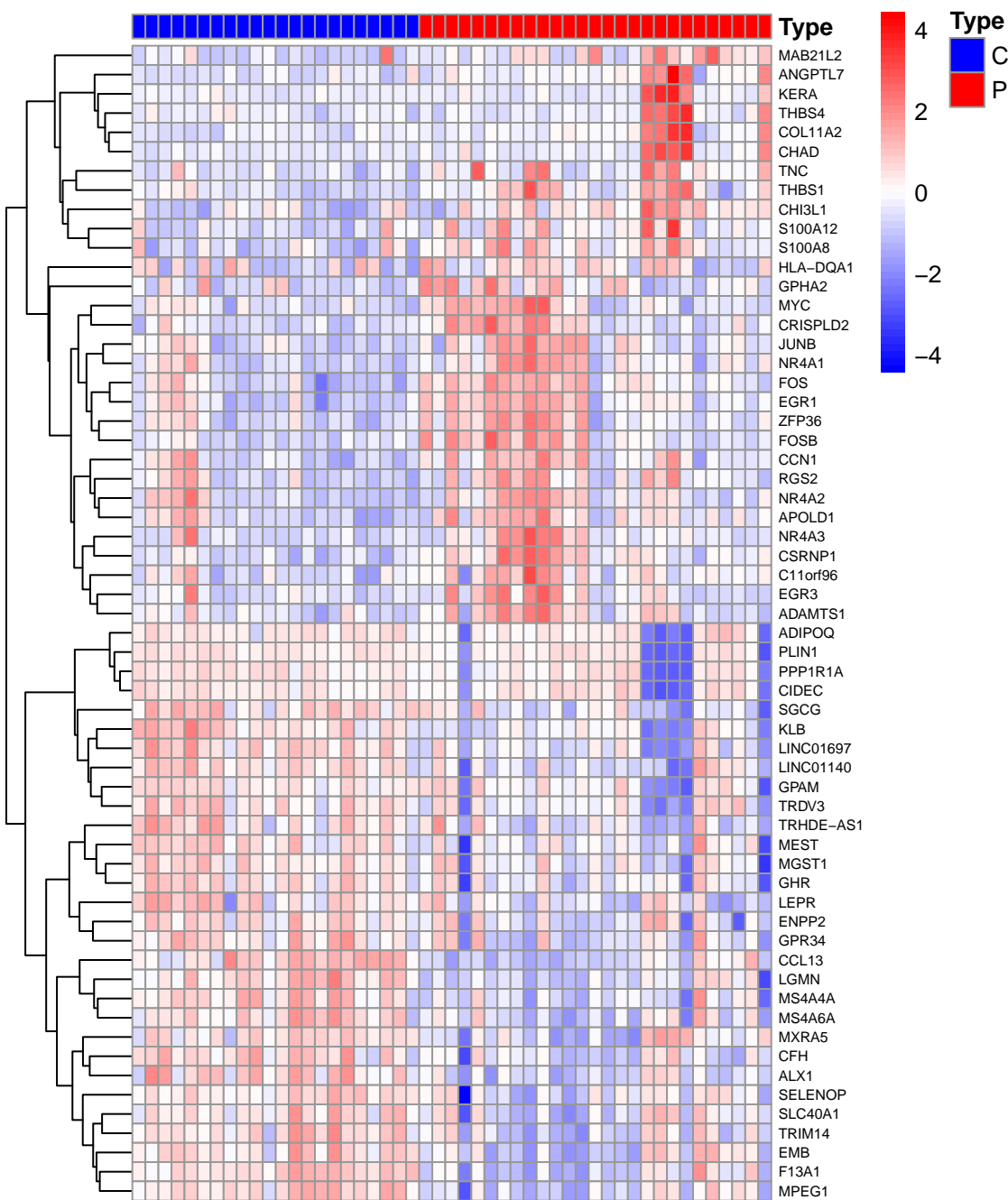

Supplement: Supplementary file 1 [file DataSheet_1.zip › 1.deg+wgcna/3.DIFF_heatmap.pdf]

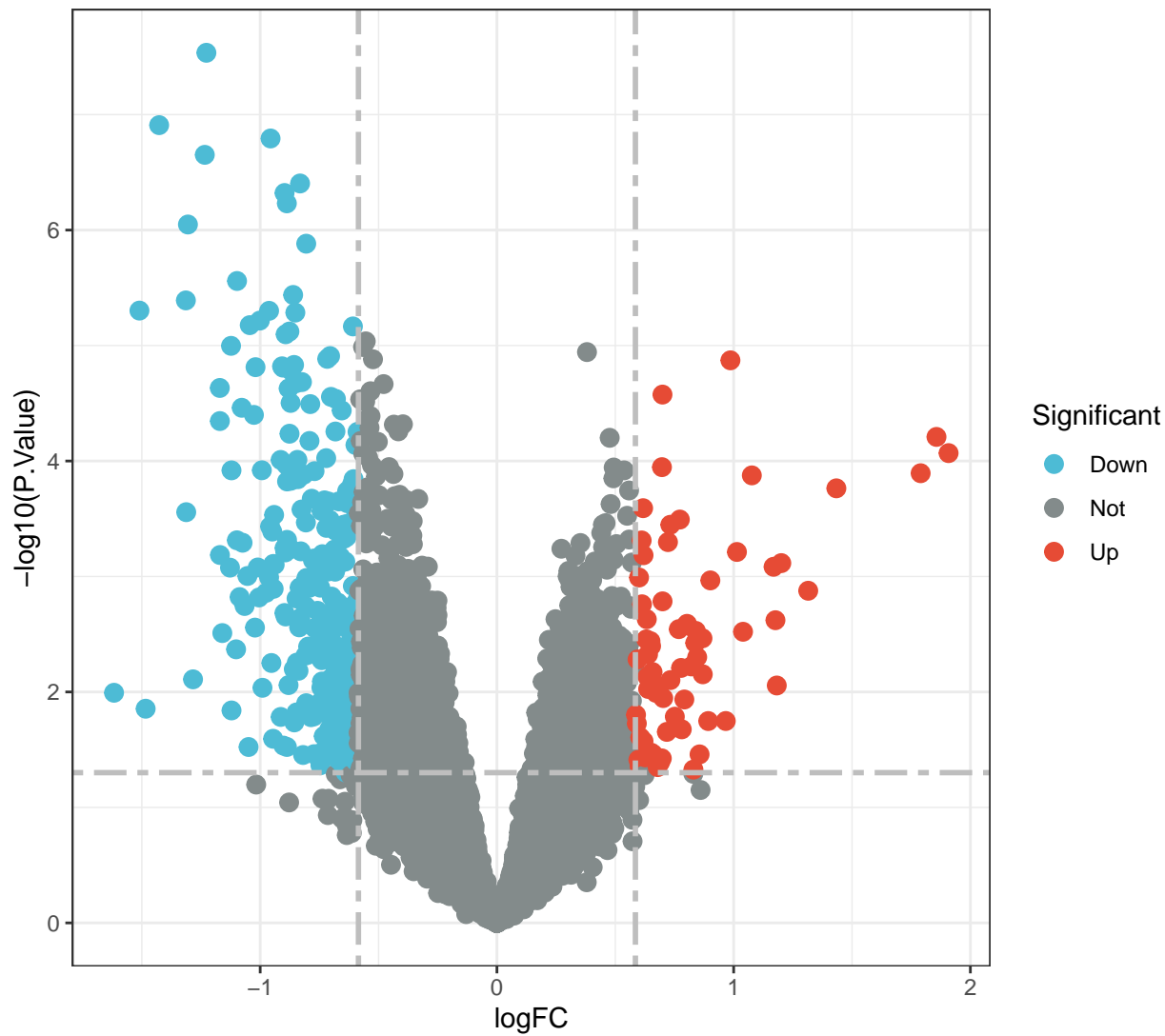

Supplement: Supplementary file 1 [file DataSheet_1.zip › 1.deg+wgcna/3.DIFF_vol.pdf]

### Scale independence

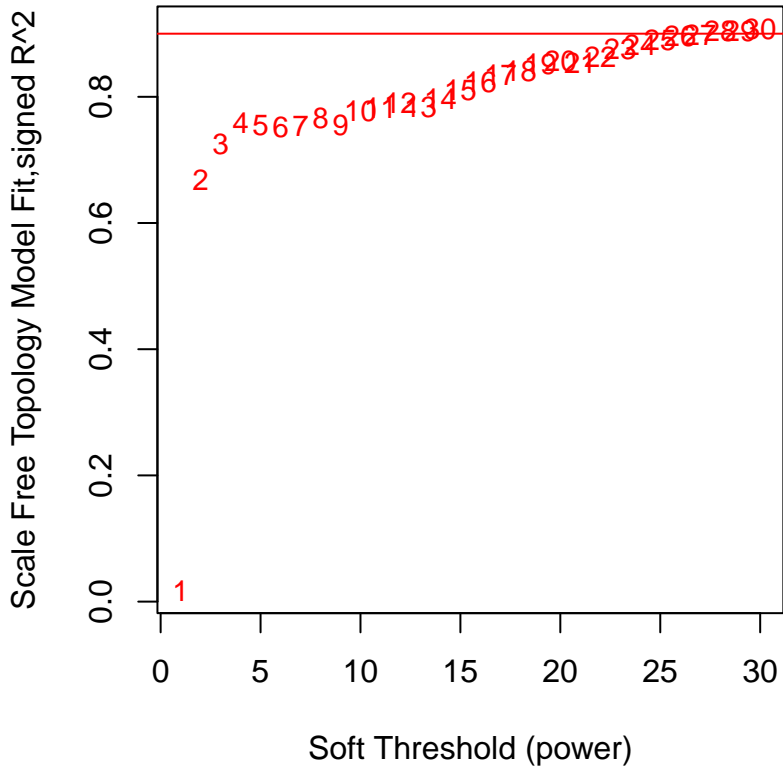

### Mean connectivity

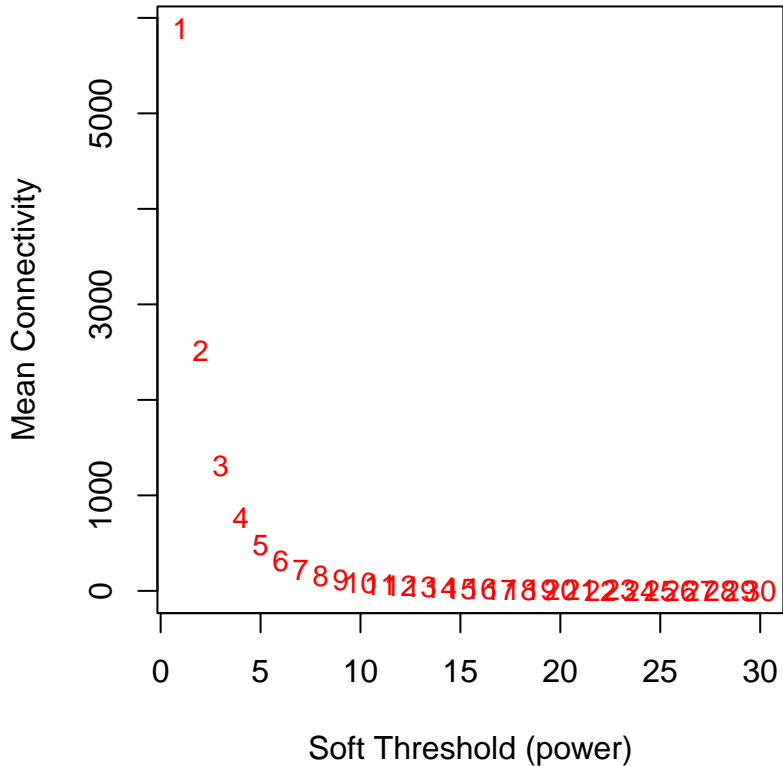

Supplement: Supplementary file 1 [file DataSheet_1.zip › 1.deg+wgcna/3_Scale independence.pdf]

# Gene clustering on TOM-based dissimilarity

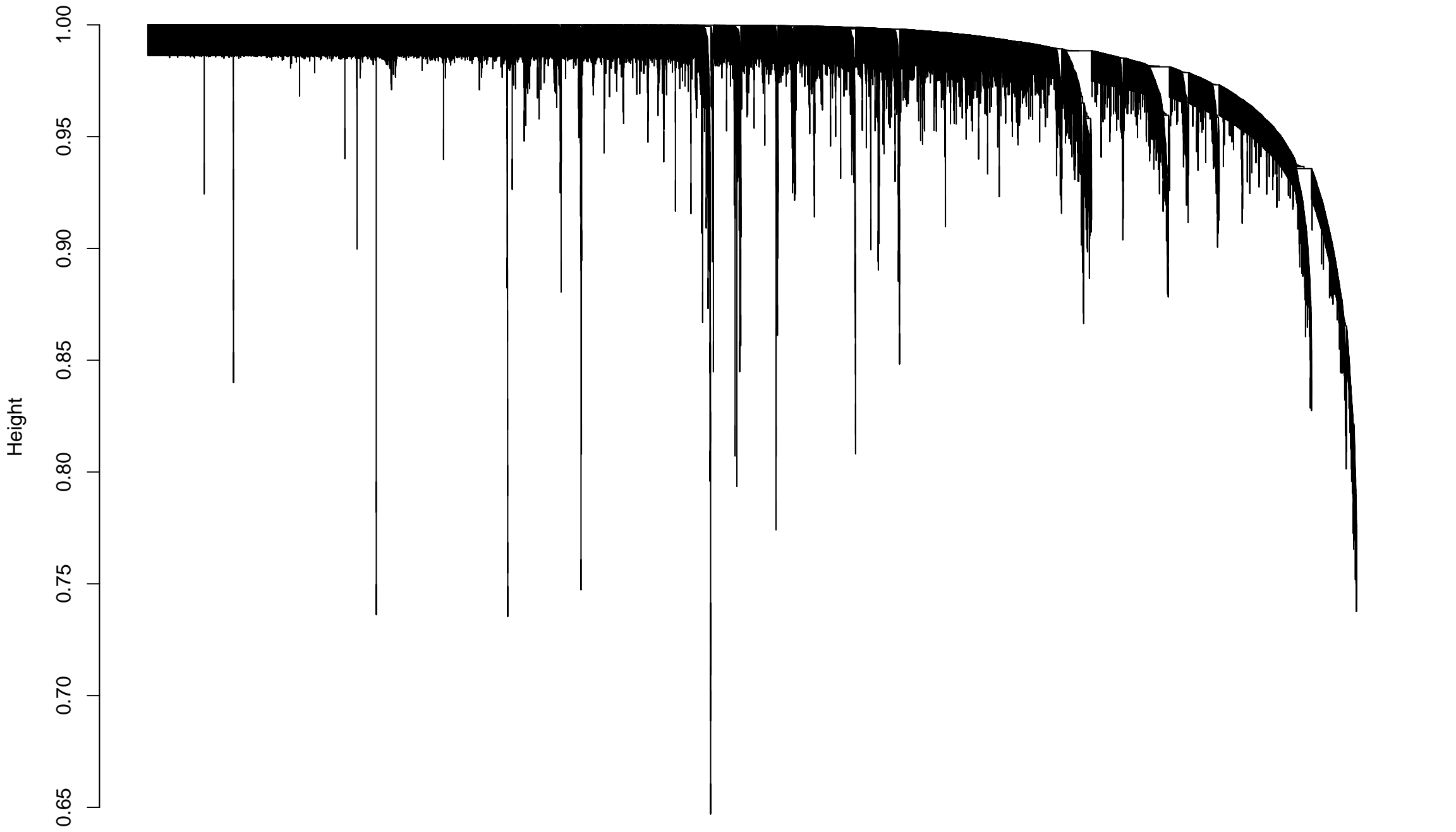

Supplement: Supplementary file 1 [file DataSheet_1.zip › 1.deg+wgcna/4_Gene clustering on TOM-based dissimilarity.pdf]

# Gene dendrogram and module colors

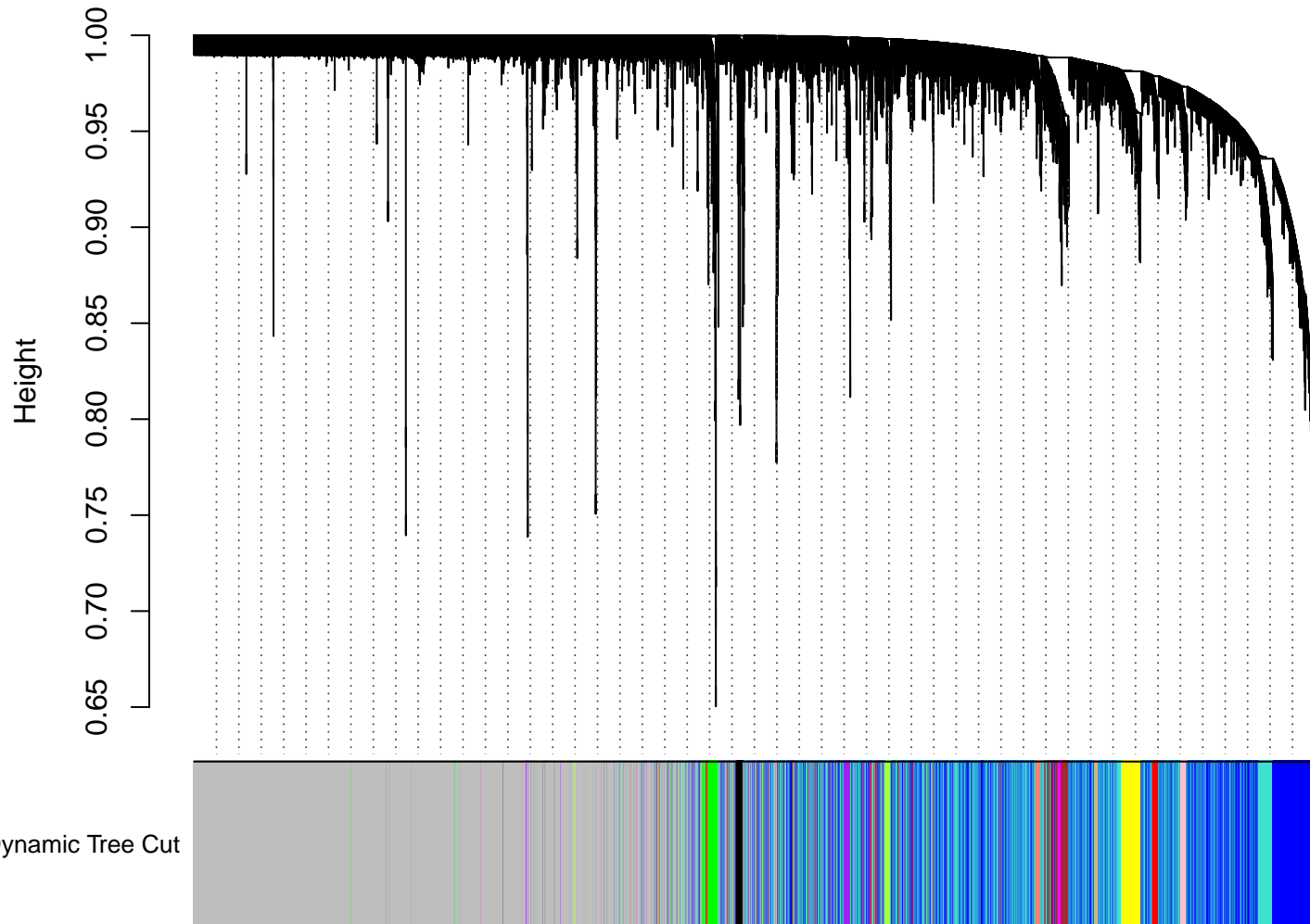

Supplement: Supplementary file 1 [file DataSheet_1.zip › 1.deg+wgcna/5_Dynamic Tree Cut.pdf]

# Clustering of module eigengenes

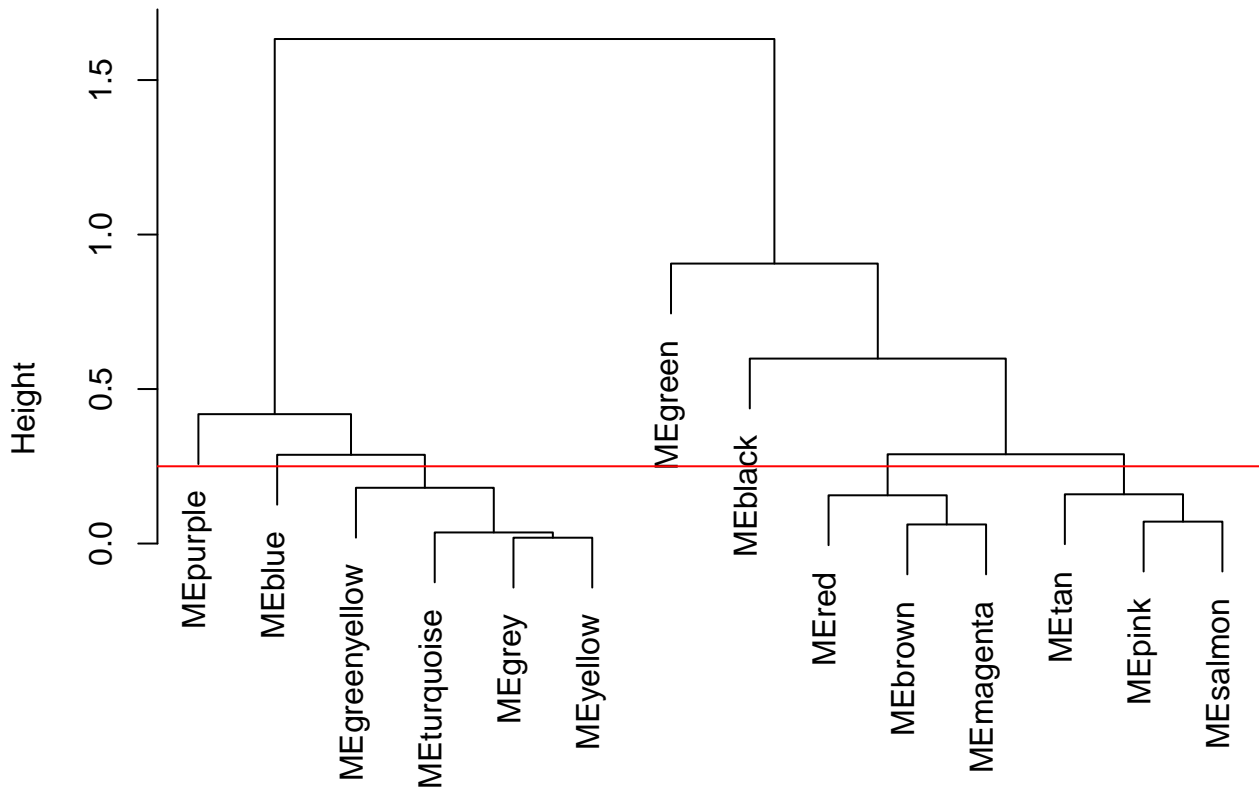

Supplement: Supplementary file 1 [file DataSheet_1.zip › 1.deg+wgcna/6_Clustering of module eigengenes.pdf]

# Cluster Dendrogram

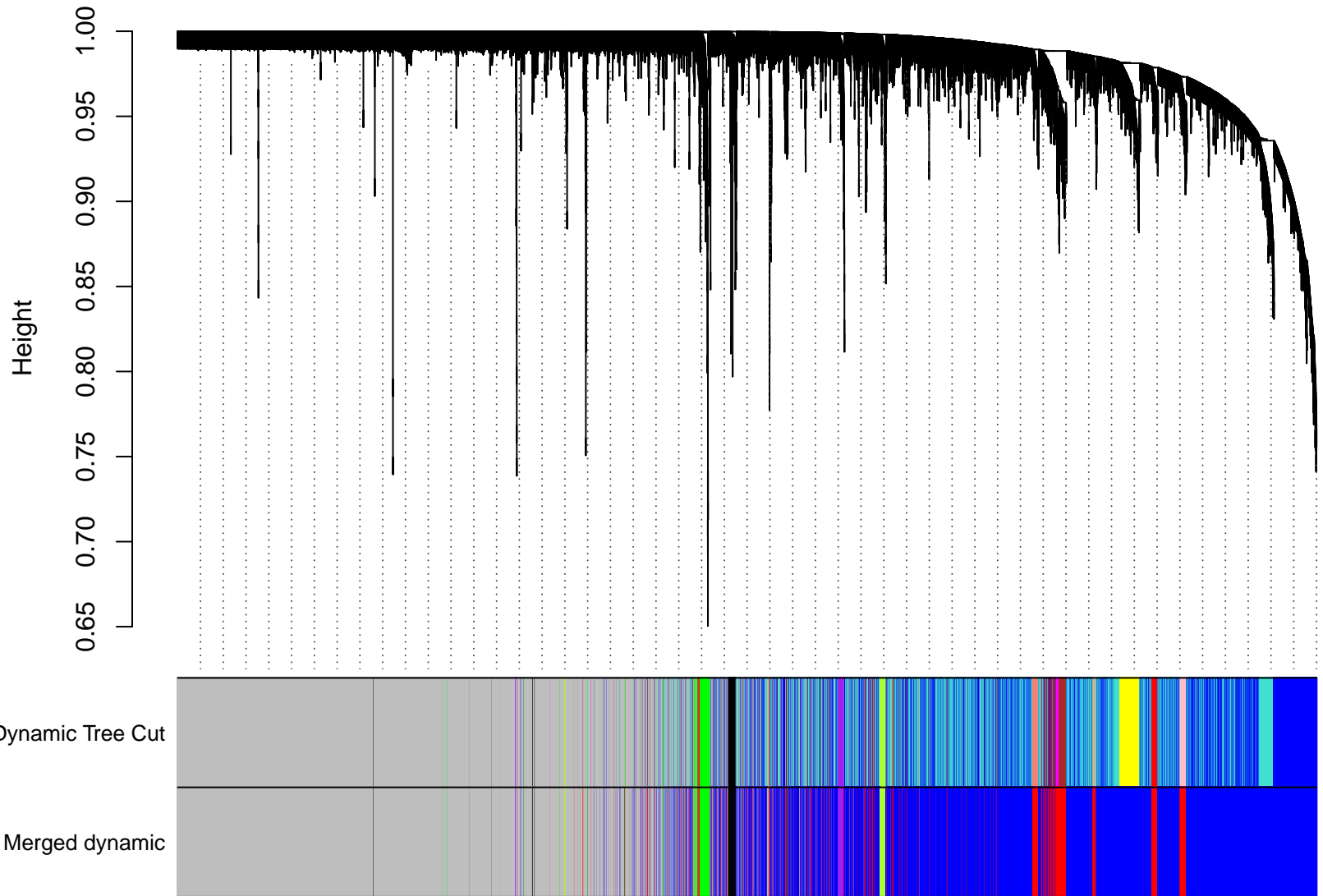

Supplement: Supplementary file 1 [file DataSheet_1.zip › 1.deg+wgcna/7_merged dynamic.pdf]

Module–trait relationships

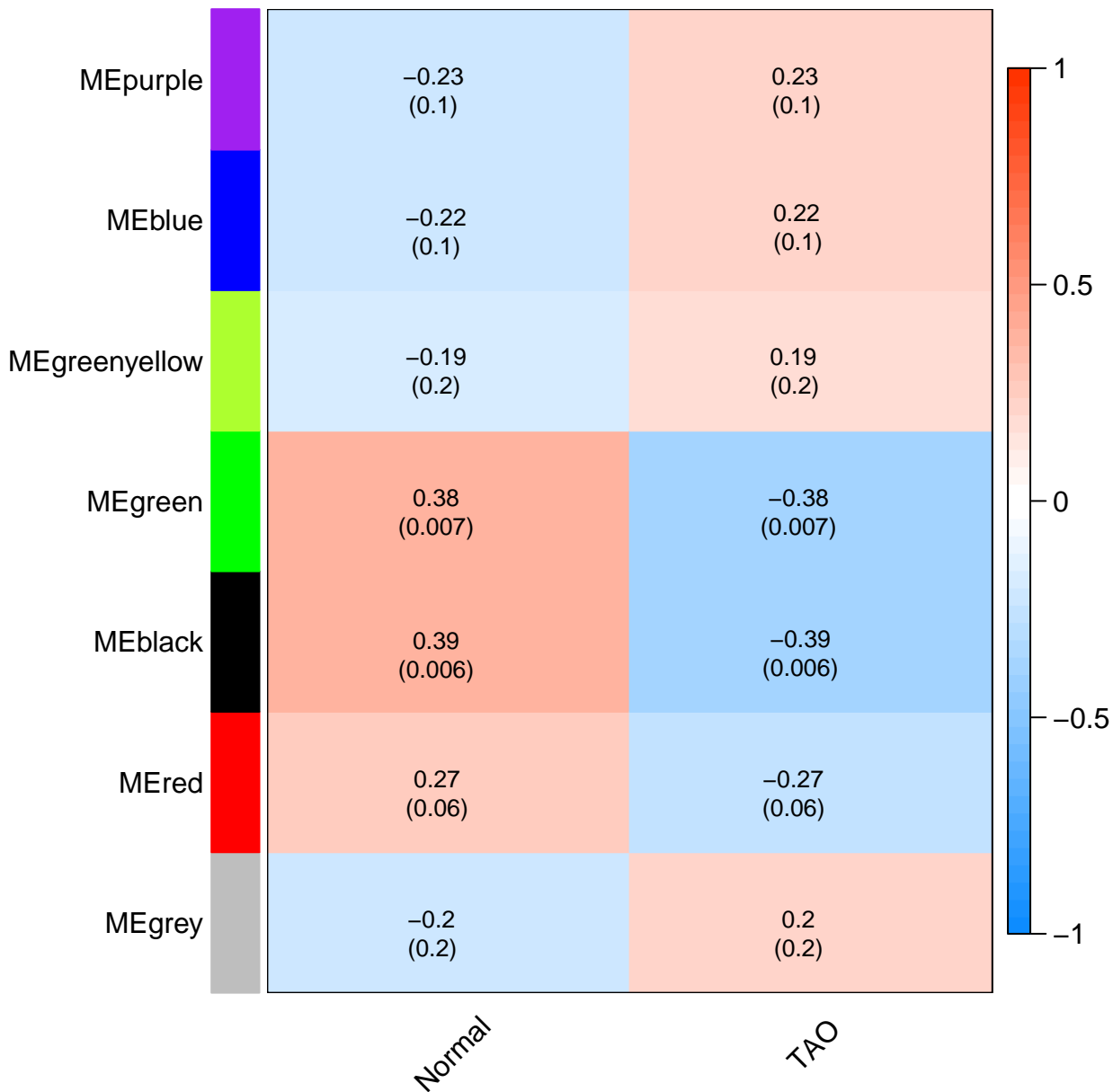

Supplement: Supplementary file 1 [file DataSheet_1.zip › 1.deg+wgcna/8_Module-trait relationships.pdf]

**Module membership vs. gene significance**  
**cor=0.5, p=1.2e-12**

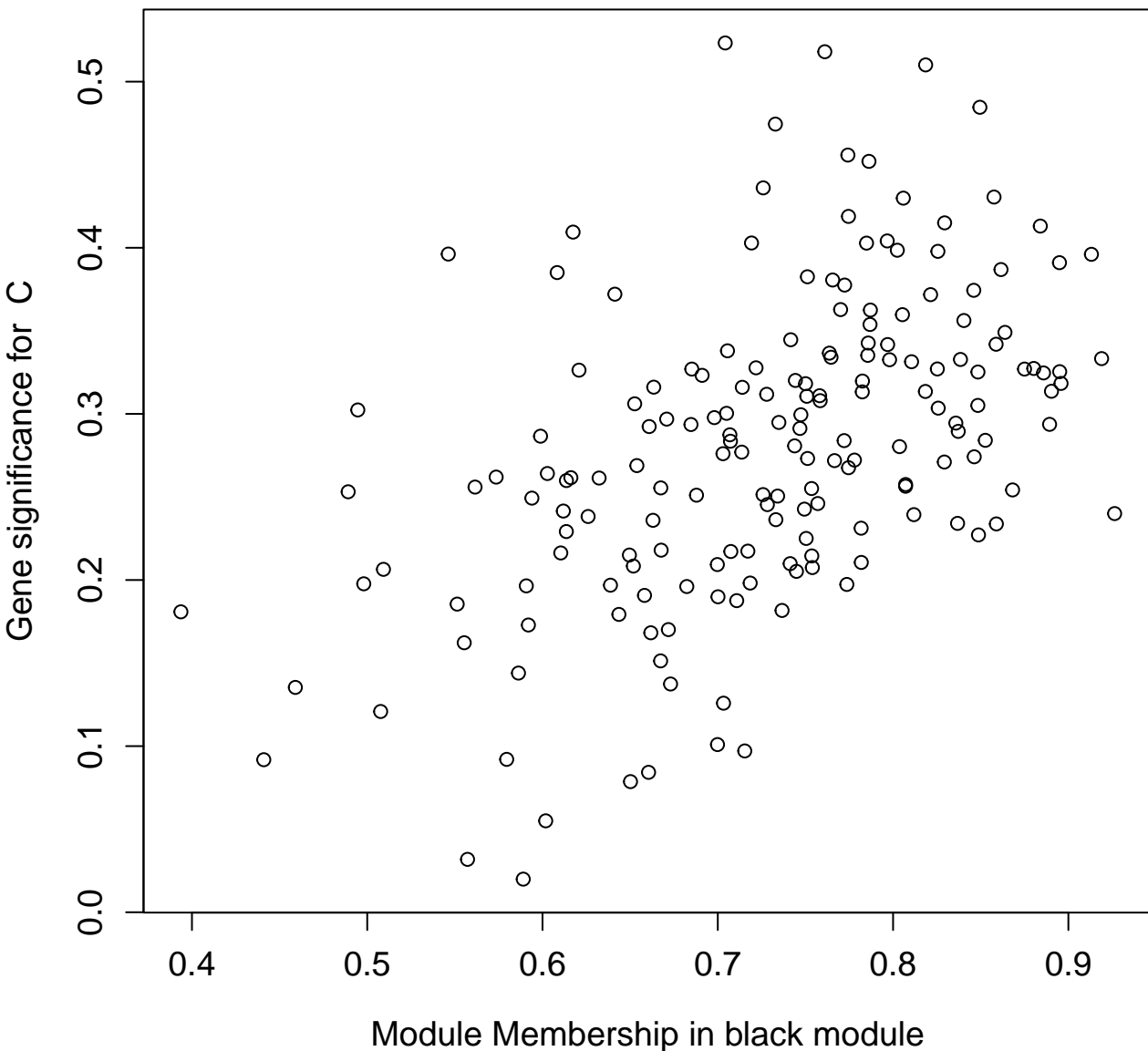

Supplement: Supplementary file 1 [file DataSheet_1.zip › 1.deg+wgcna/9_C_black_Module membership vs gene significance.pdf]

**Module membership vs. gene significance**  
**cor=0.19, p=7.4e-76**

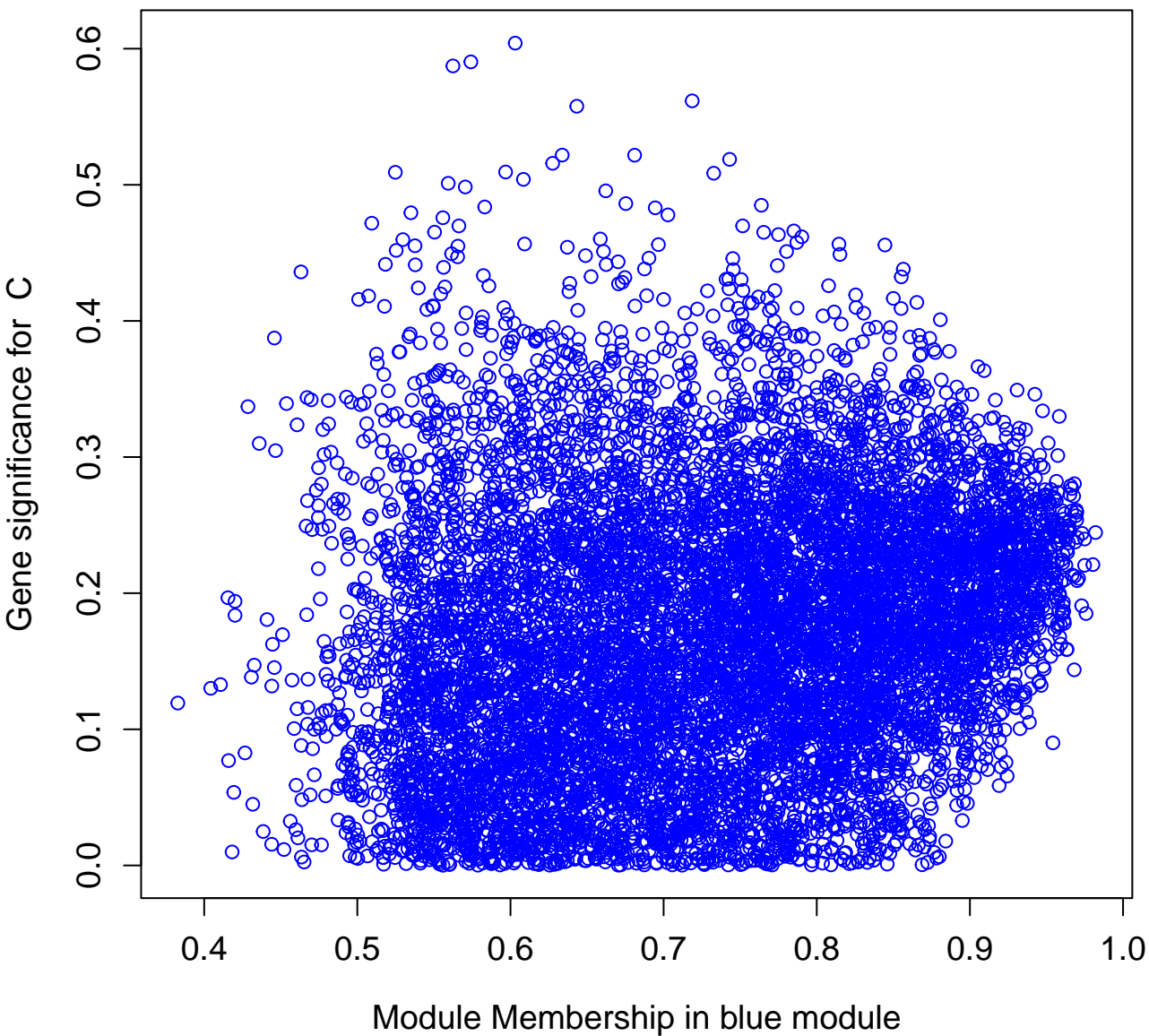

Supplement: Supplementary file 1 [file DataSheet_1.zip › 1.deg+wgcna/9_C_blue_Module membership vs gene significance.pdf]

**Module membership vs. gene significance**  
**cor=0.2, p=0.00044**

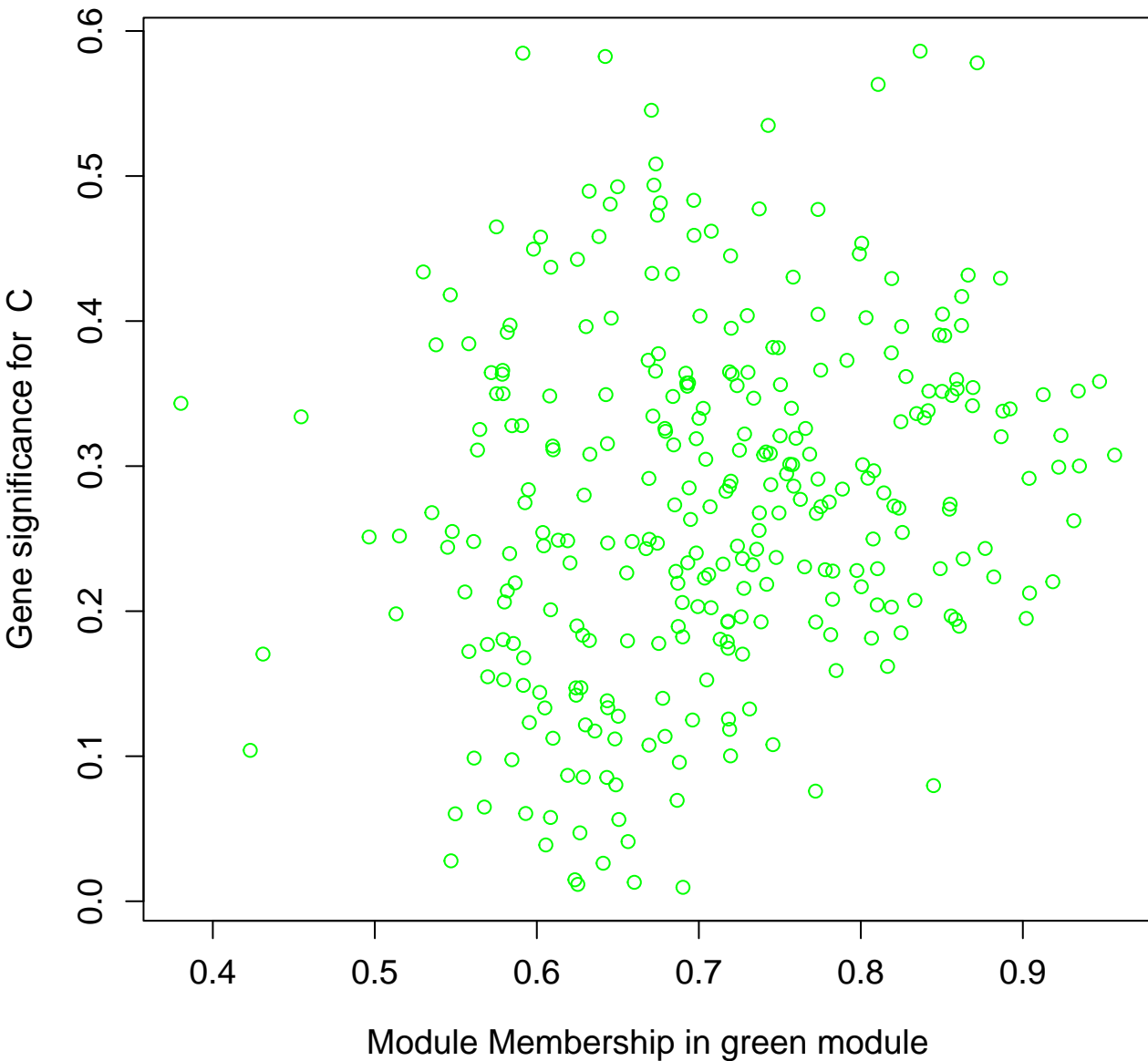

Supplement: Supplementary file 1 [file DataSheet_1.zip › 1.deg+wgcna/9_C_green_Module membership vs gene significance.pdf]

**Module membership vs. gene significance**  
**cor=0.13, p=0.13**

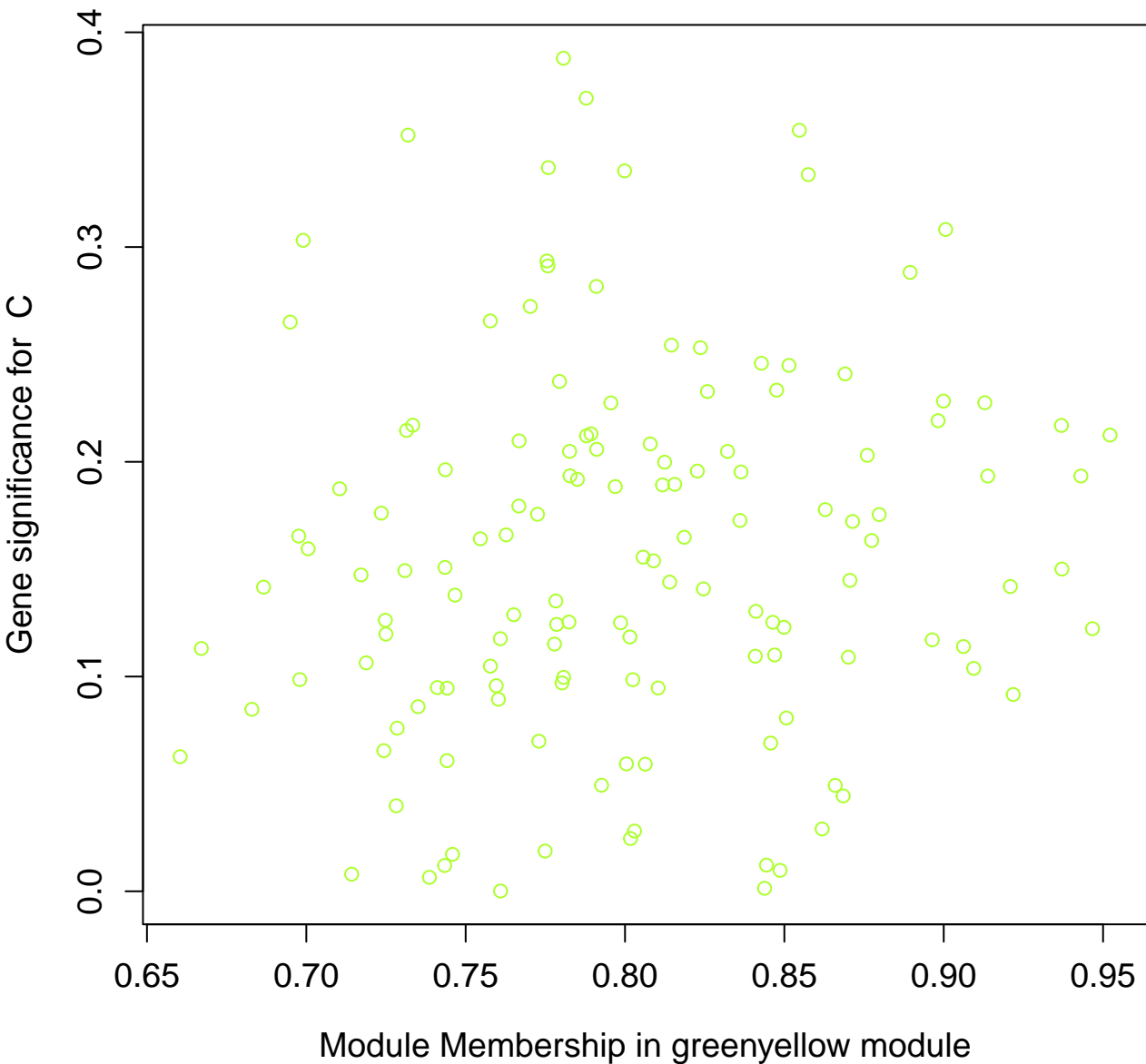

Supplement: Supplementary file 1 [file DataSheet_1.zip › 1.deg+wgcna/9_C_greenyellow_Module membership vs gene significance.pdf]

**Module membership vs. gene significance**  
**cor=0.0079, p=0.44**

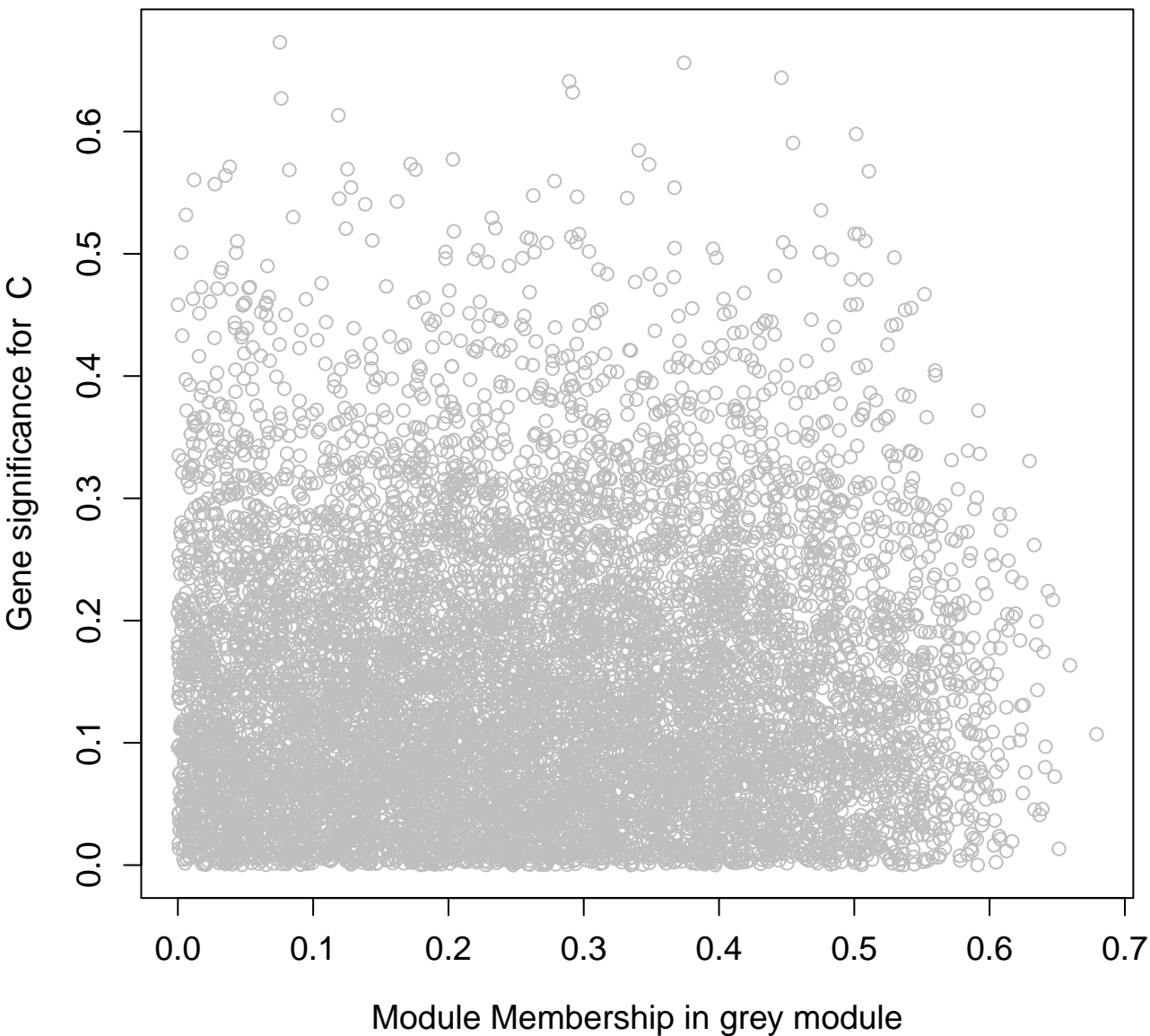

Supplement: Supplementary file 1 [file DataSheet_1.zip › 1.deg+wgcna/9_C_grey_Module membership vs gene significance.pdf]

**Module membership vs. gene significance**  
**cor=0.21, p=0.012**

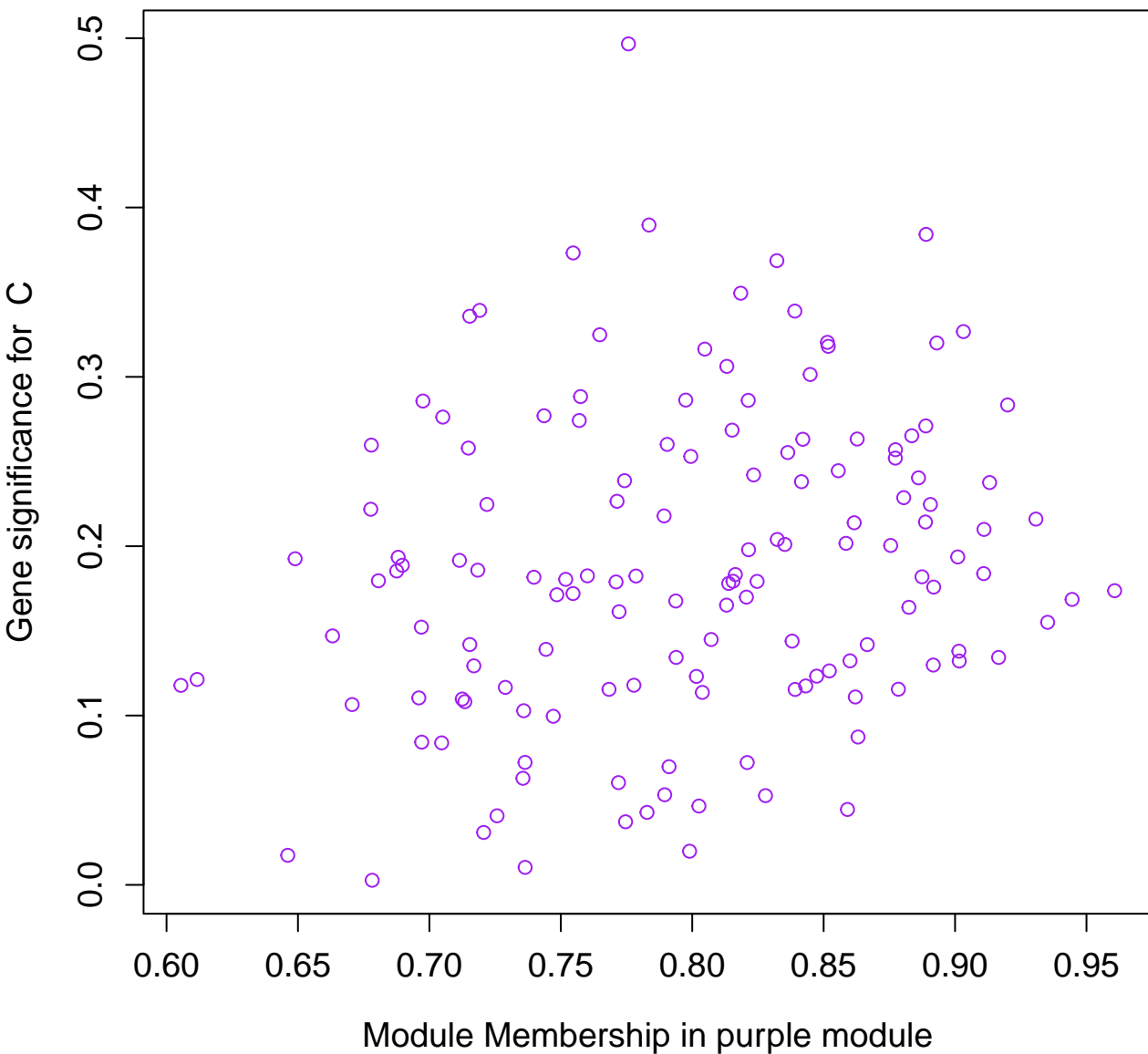

Supplement: Supplementary file 1 [file DataSheet_1.zip › 1.deg+wgcna/9_C_purple_Module membership vs gene significance.pdf]

**Module membership vs. gene significance**  
**cor=0.18, p=8.9e-12**

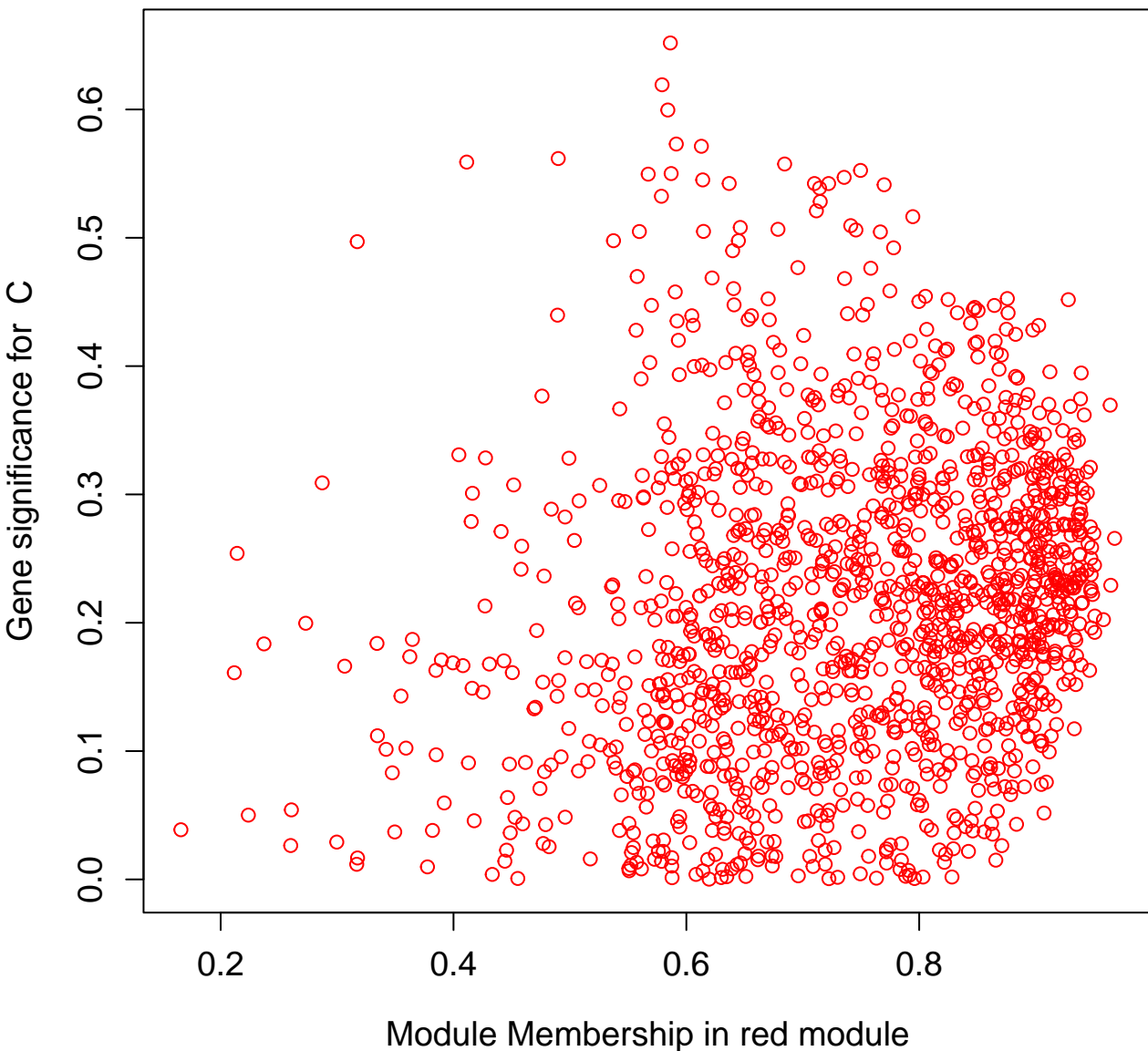

Supplement: Supplementary file 1 [file DataSheet_1.zip › 1.deg+wgcna/9_C_red_Module membership vs gene significance.pdf]

**Module membership vs. gene significance**  
**cor=0.5, p=1.2e-12**

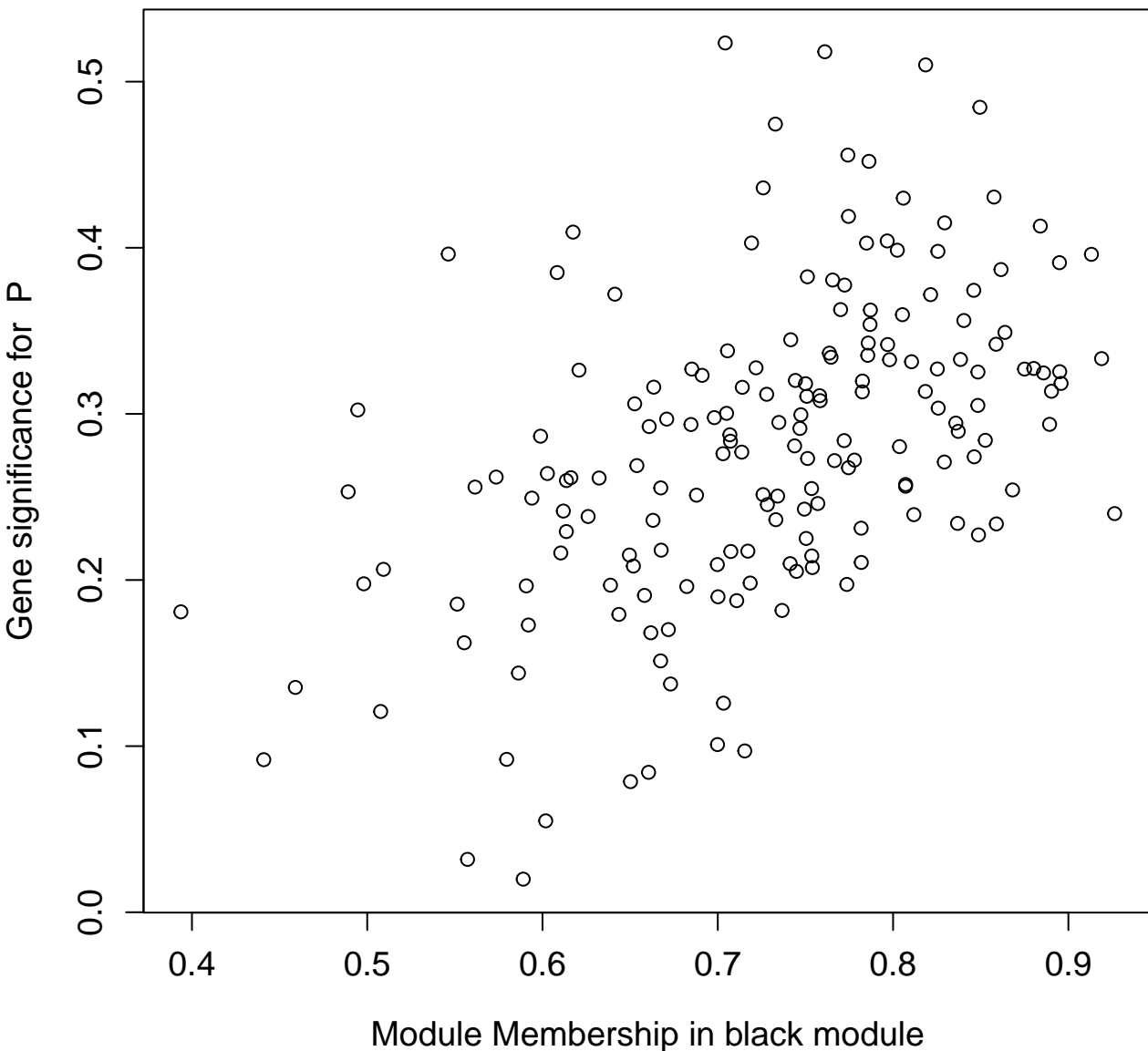

Supplement: Supplementary file 1 [file DataSheet_1.zip › 1.deg+wgcna/9_P_black_Module membership vs gene significance.pdf]

**Module membership vs. gene significance**  
**cor=0.19, p=7.4e-76**

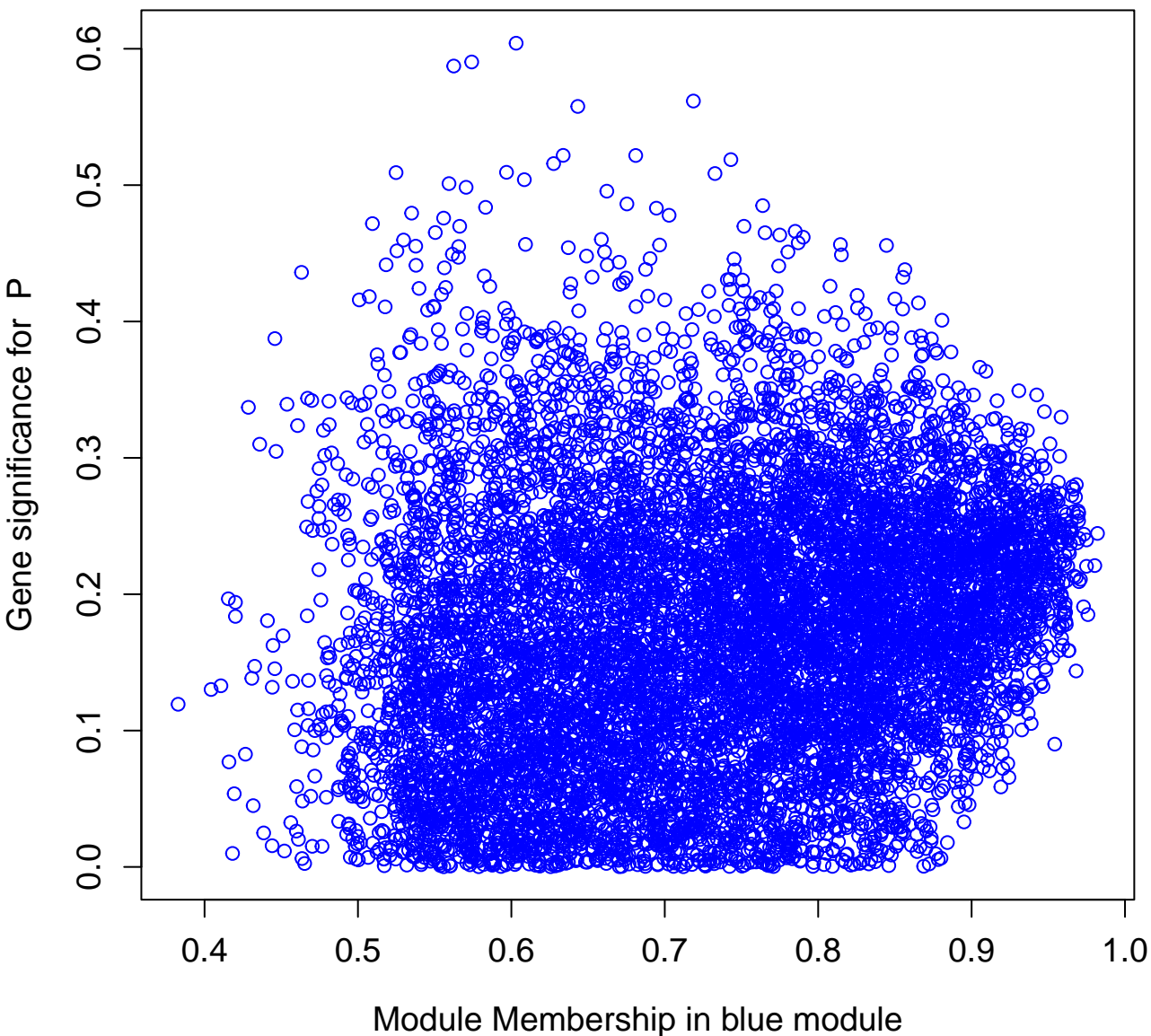

Supplement: Supplementary file 1 [file DataSheet_1.zip › 1.deg+wgcna/9_P_blue_Module membership vs gene significance.pdf]

**Module membership vs. gene significance**  
**cor=0.2, p=0.00044**

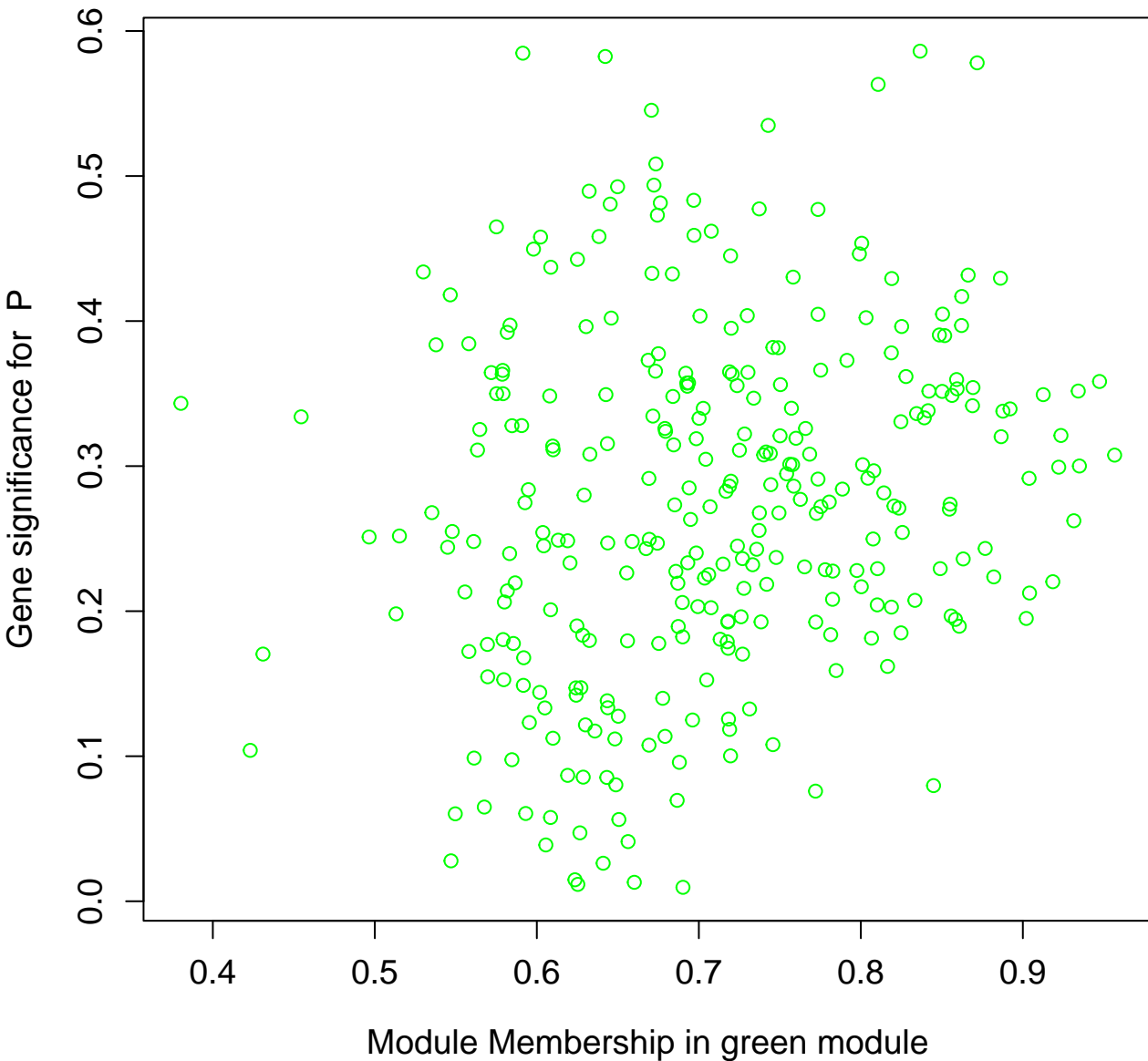

Supplement: Supplementary file 1 [file DataSheet_1.zip › 1.deg+wgcna/9_P_green_Module membership vs gene significance.pdf]

**Module membership vs. gene significance**  
**cor=0.13, p=0.13**

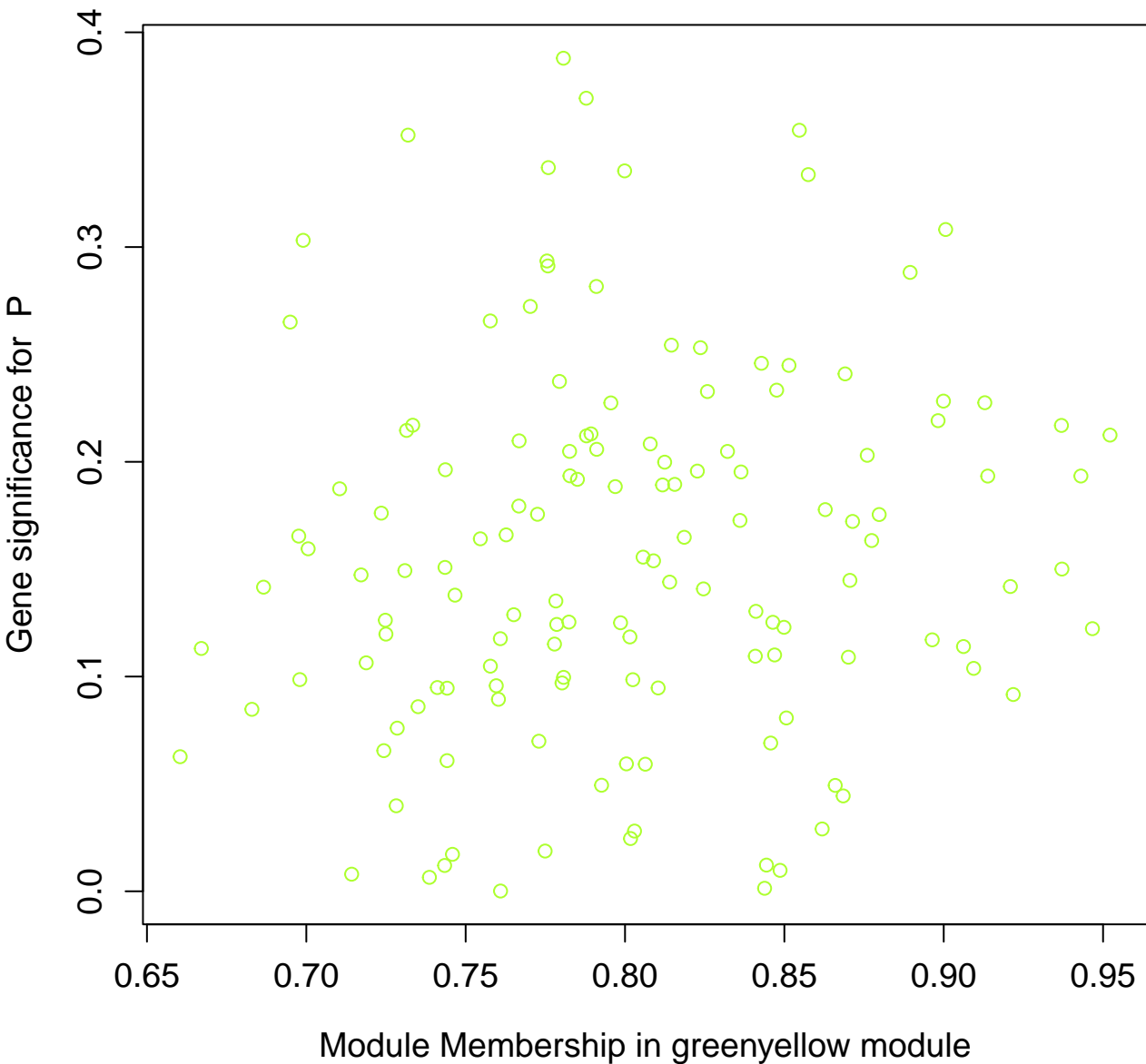

Supplement: Supplementary file 1 [file DataSheet_1.zip › 1.deg+wgcna/9_P_greenyellow_Module membership vs gene significance.pdf]

**Module membership vs. gene significance**  
**cor=0.0079, p=0.44**

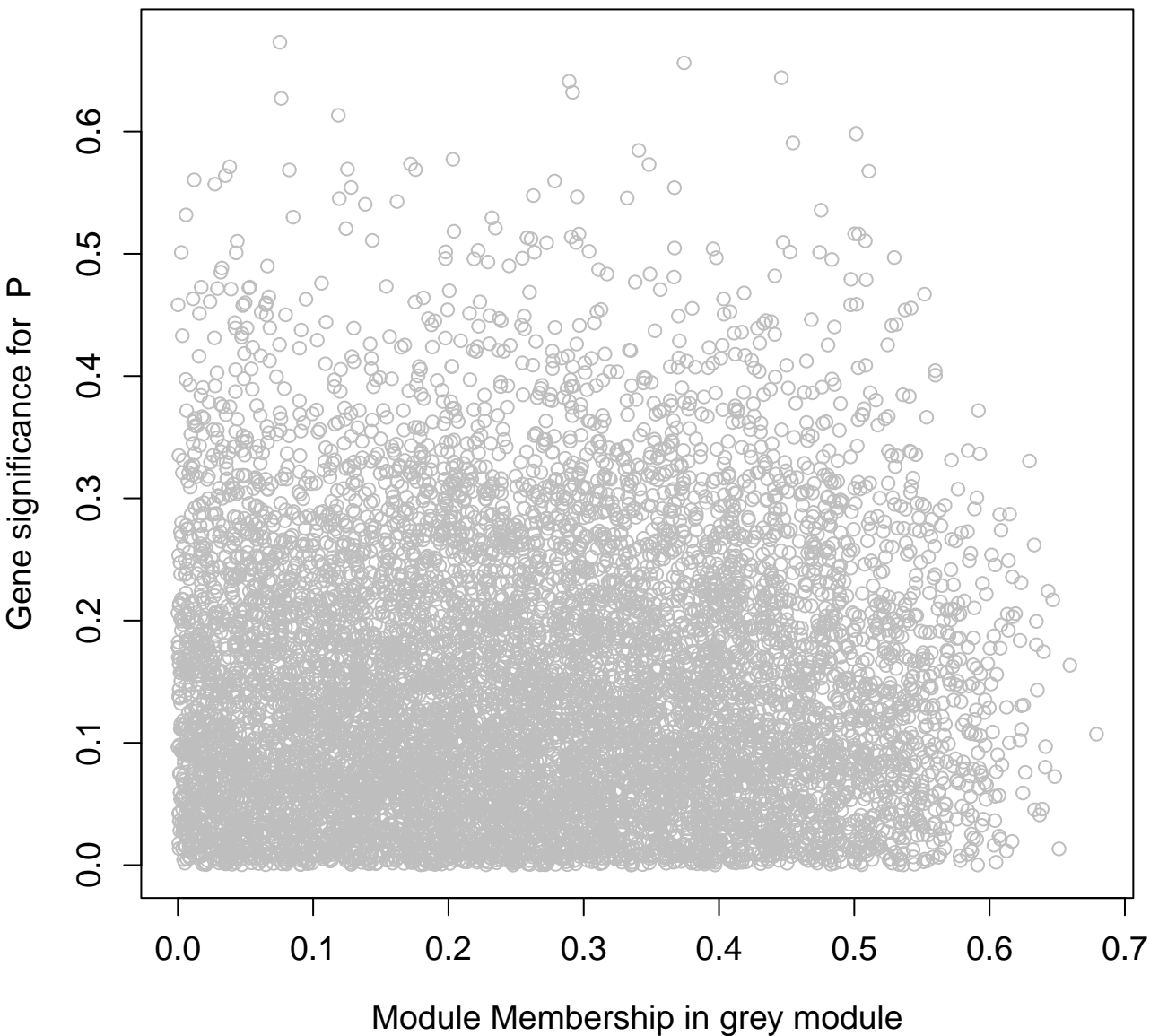

Supplement: Supplementary file 1 [file DataSheet_1.zip › 1.deg+wgcna/9_P_grey_Module membership vs gene significance.pdf]

**Module membership vs. gene significance**  
**cor=0.21, p=0.012**

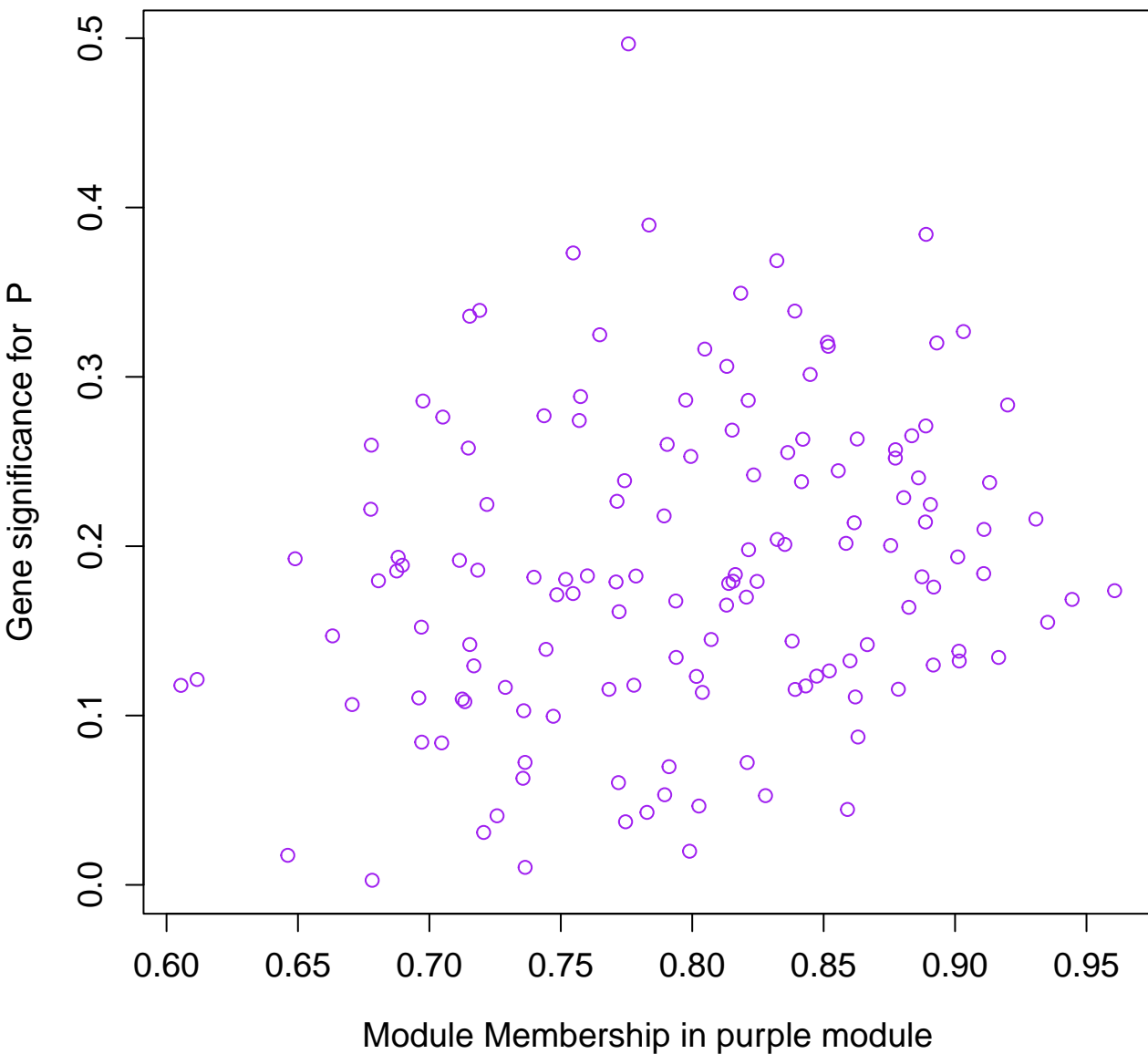

Supplement: Supplementary file 1 [file DataSheet_1.zip › 1.deg+wgcna/9_P_purple_Module membership vs gene significance.pdf]

**Module membership vs. gene significance**  
**cor=0.18, p=8.9e-12**

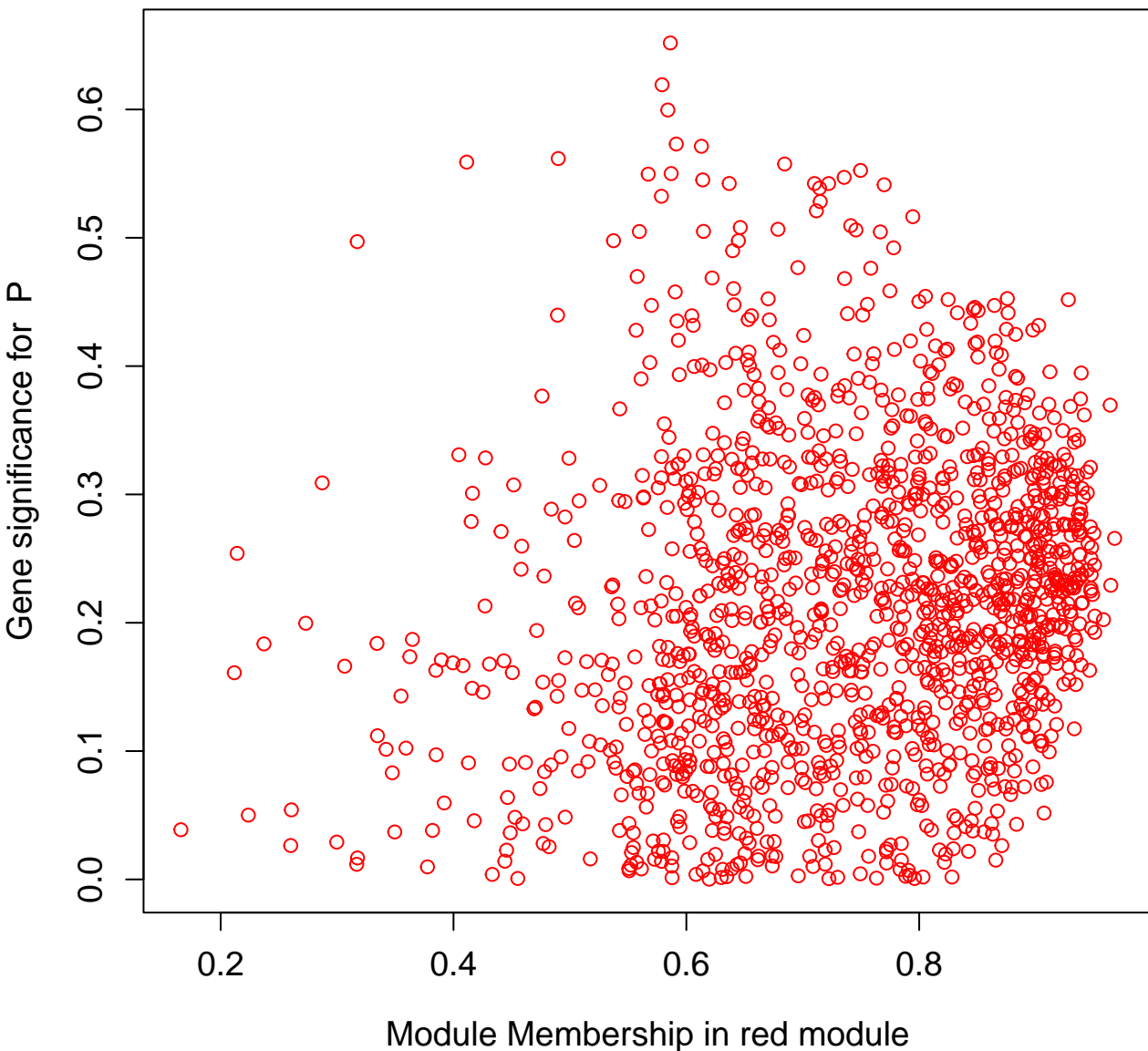

Supplement: Supplementary file 1 [file DataSheet_1.zip › 1.deg+wgcna/9_P_red_Module membership vs gene significance.pdf]

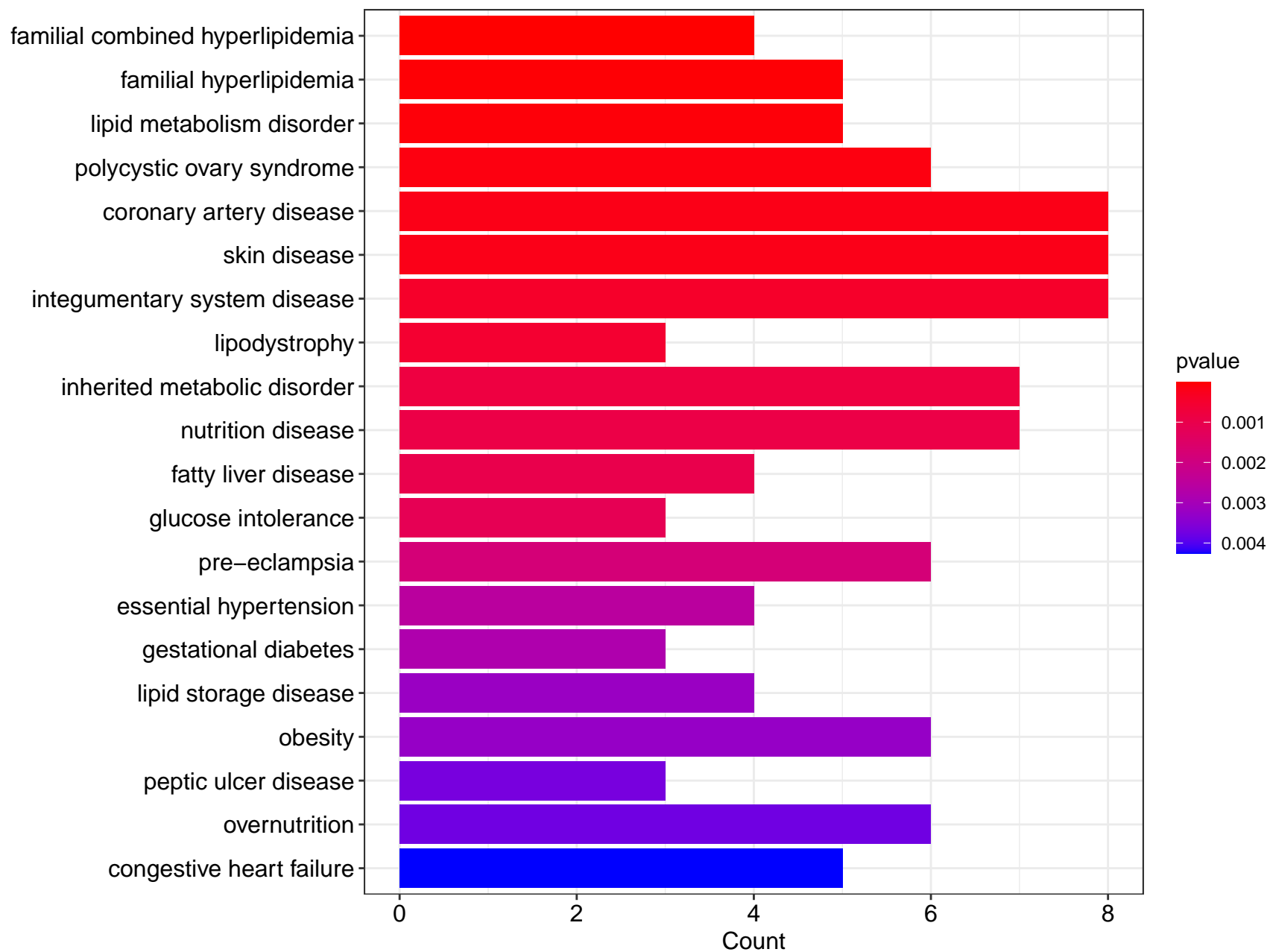

Supplement: Supplementary file 2 [file DataSheet_2.zip › 2.GO+KEGG+DO/DO_barplot.pdf]

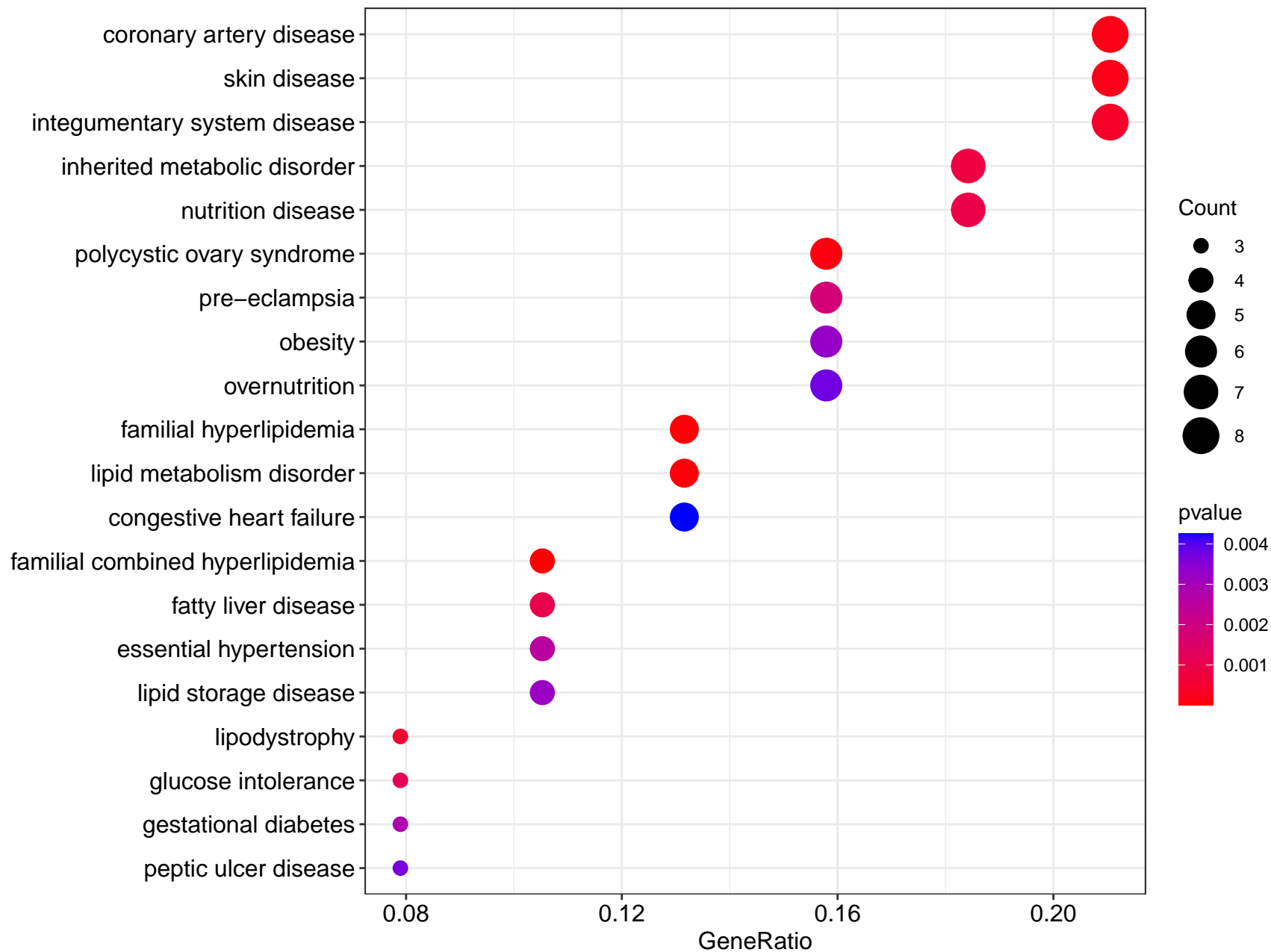

Supplement: Supplementary file 2 [file DataSheet_2.zip › 2.GO+KEGG+DO/DO_bubble.pdf]

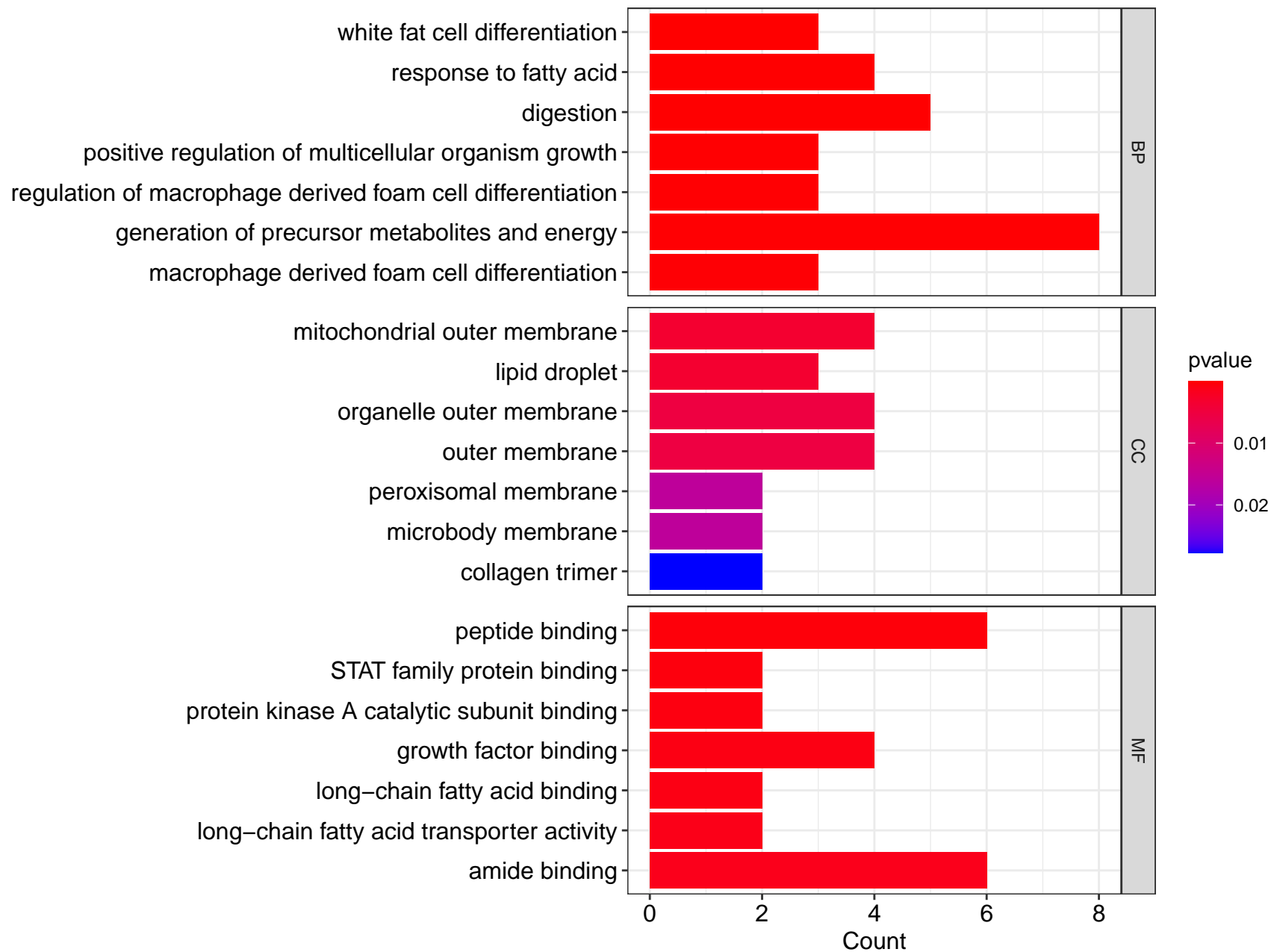

Supplement: Supplementary file 2 [file DataSheet_2.zip › 2.GO+KEGG+DO/GO_barplot.pdf]

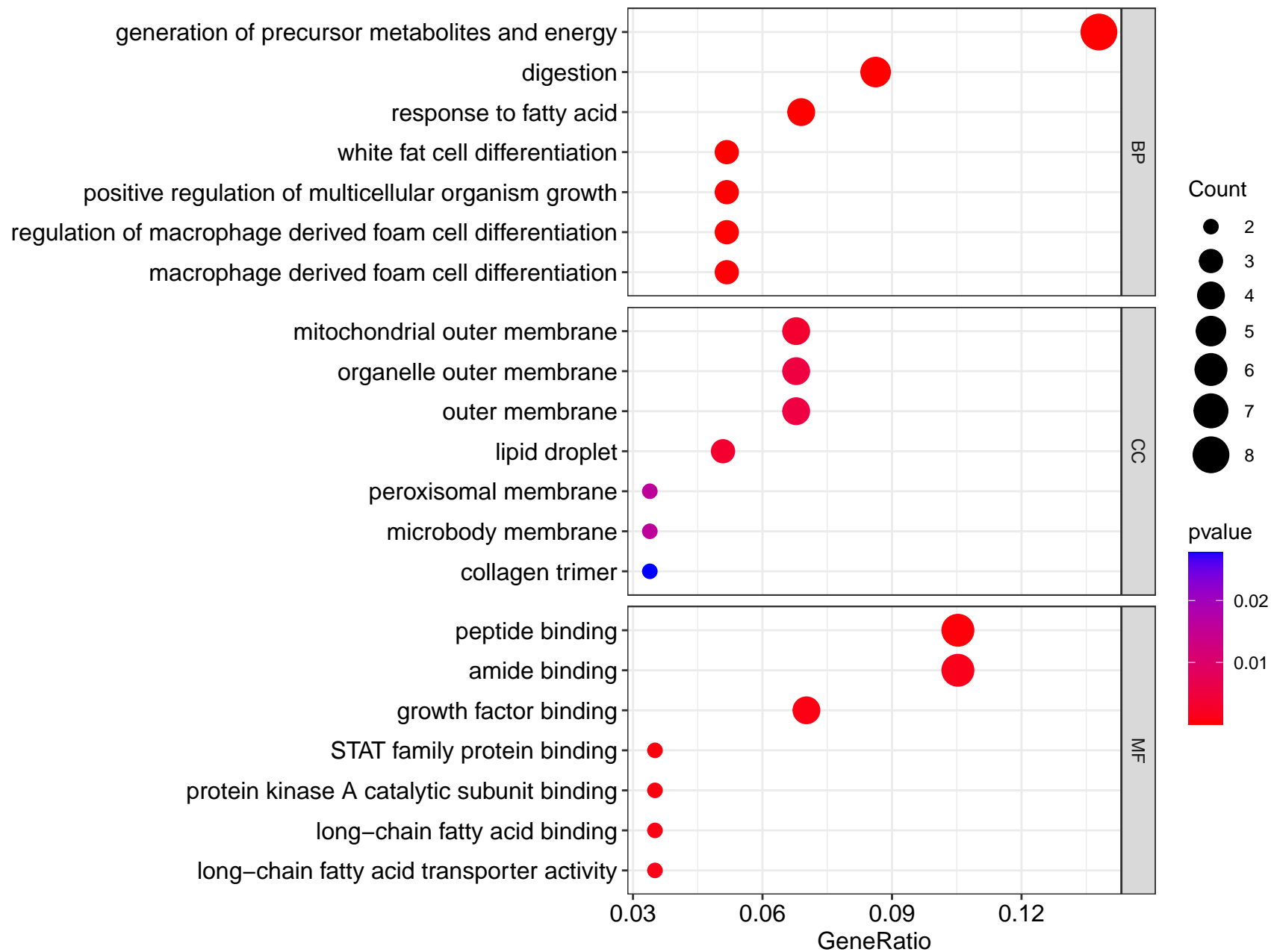

Supplement: Supplementary file 2 [file DataSheet_2.zip › 2.GO+KEGG+DO/GO_bubble.pdf]

DEGs

WGCNA

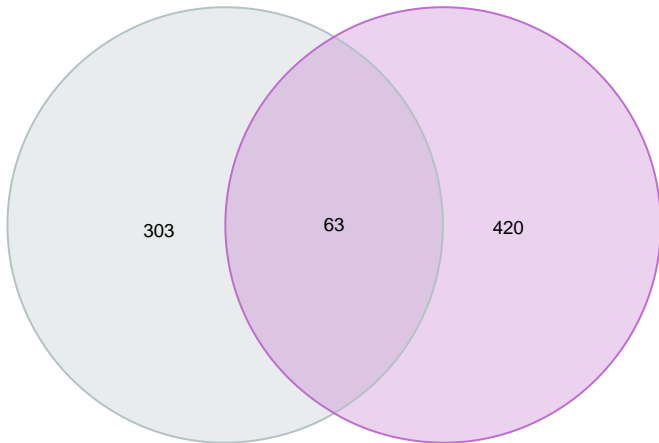

Supplement: Supplementary file 2 [file DataSheet_2.zip › 2.GO+KEGG+DO/key genes.pdf]

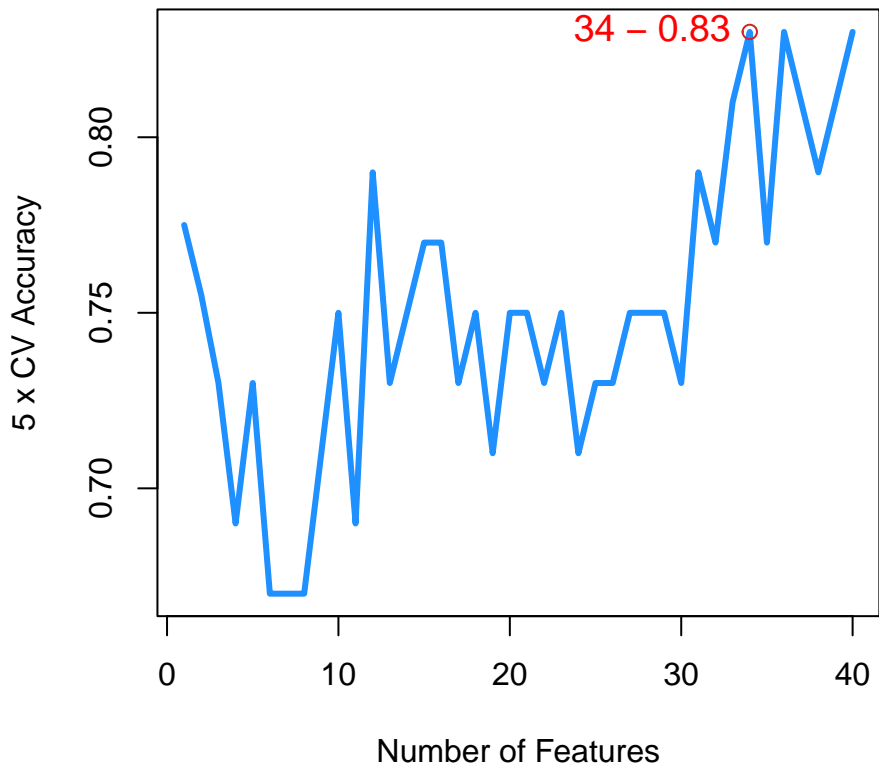

Supplement: Supplementary file 3 [file DataSheet_3.zip › 3.machine learning/1.svm/svm-accuracy.pdf]

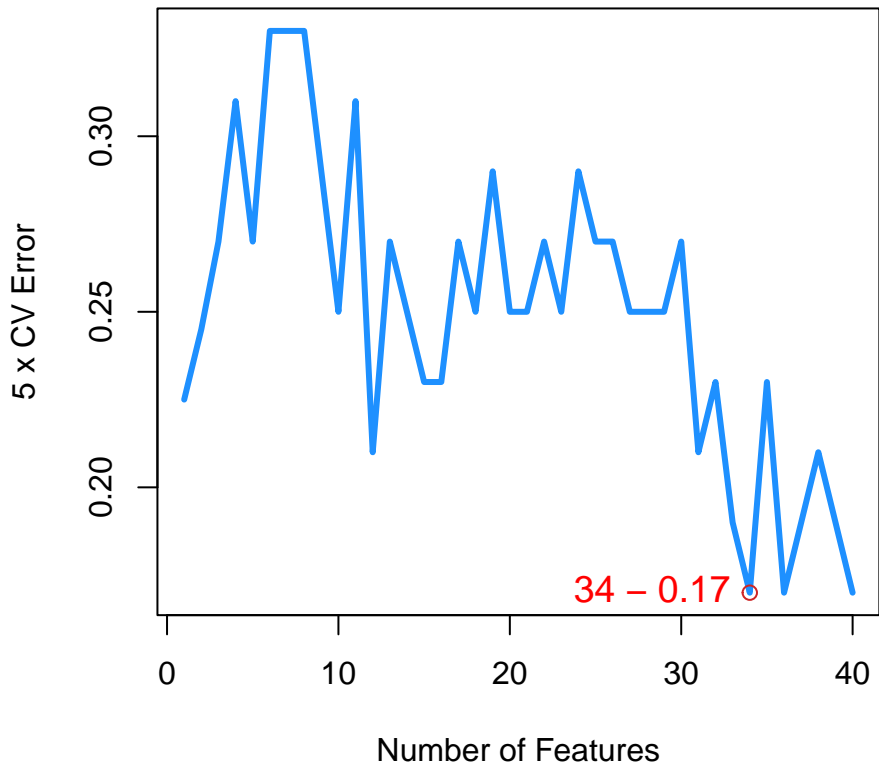

Supplement: Supplementary file 3 [file DataSheet_3.zip › 3.machine learning/1.svm/svm-error.pdf]

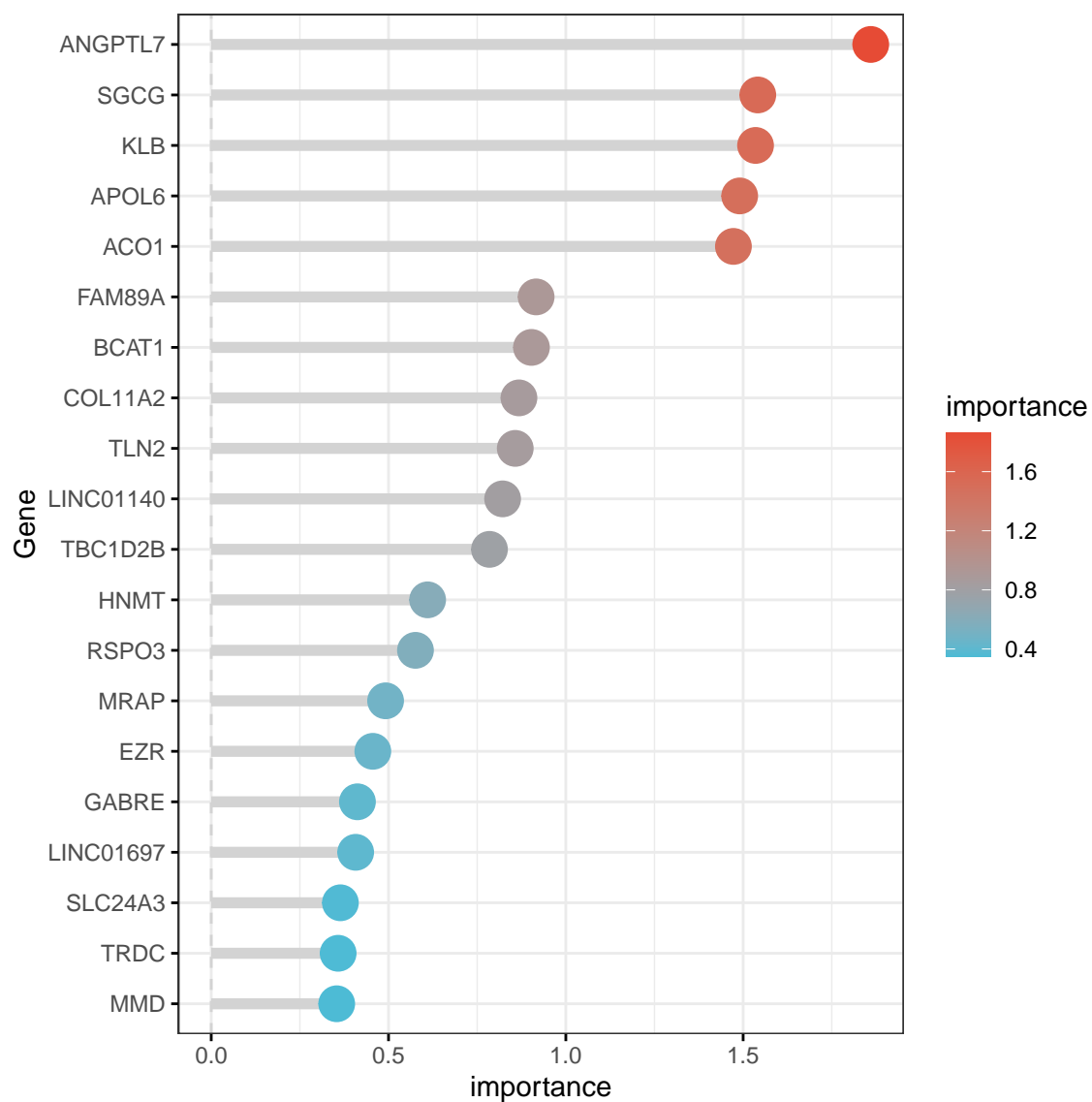

Supplement: Supplementary file 3 [file DataSheet_3.zip › 3.machine learning/2.lasso/importance.pdf]

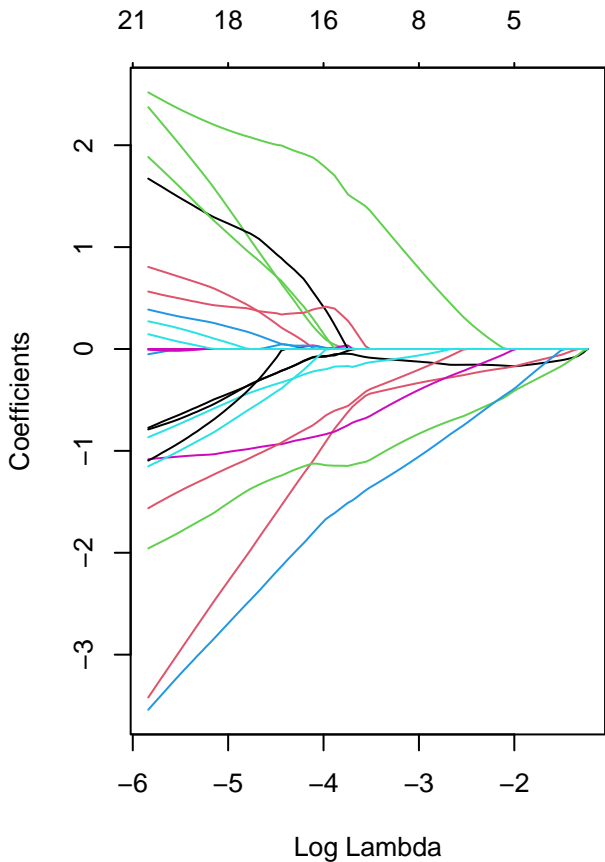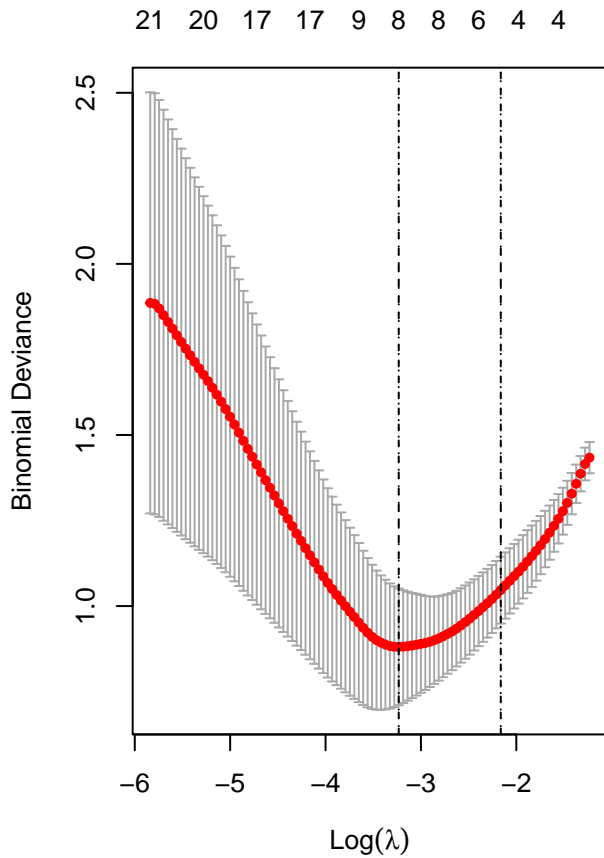

Supplement: Supplementary file 3 [file DataSheet_3.zip › 3.machine learning/2.lasso/lasso.pdf]

## Random forest

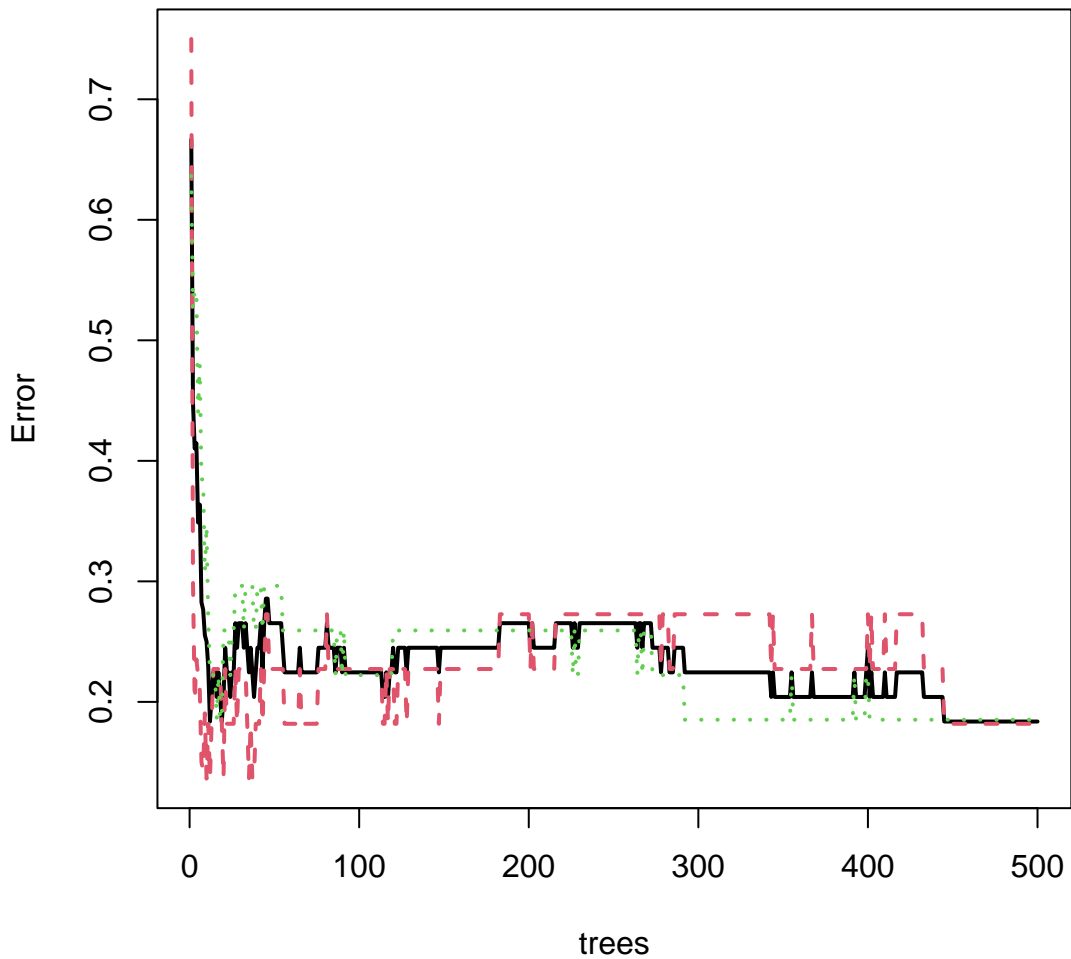

Supplement: Supplementary file 3 [file DataSheet_3.zip › 3.machine learning/3.randomForest/forest.pdf]

Gene

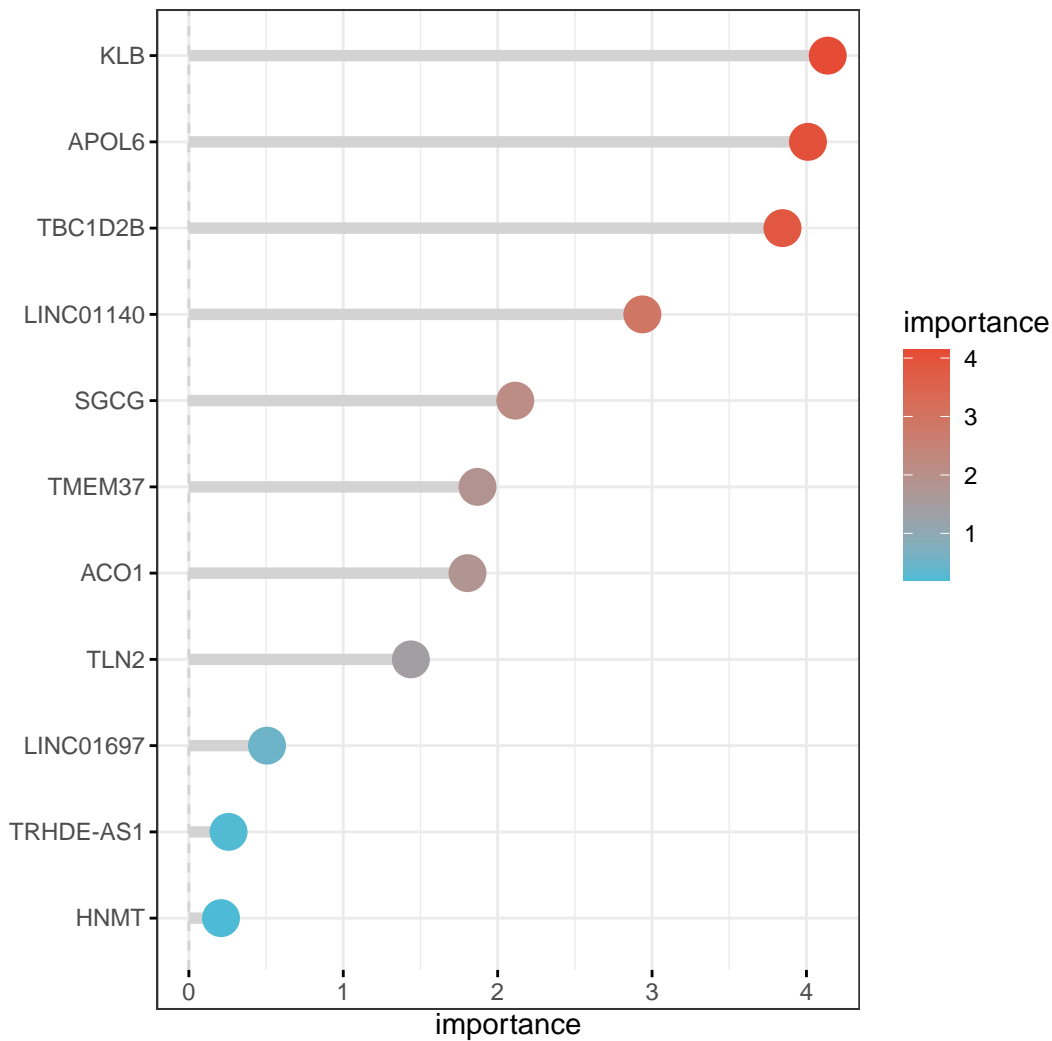

Supplement: Supplementary file 3 [file DataSheet_3.zip › 3.machine learning/3.randomForest/importance.pdf]

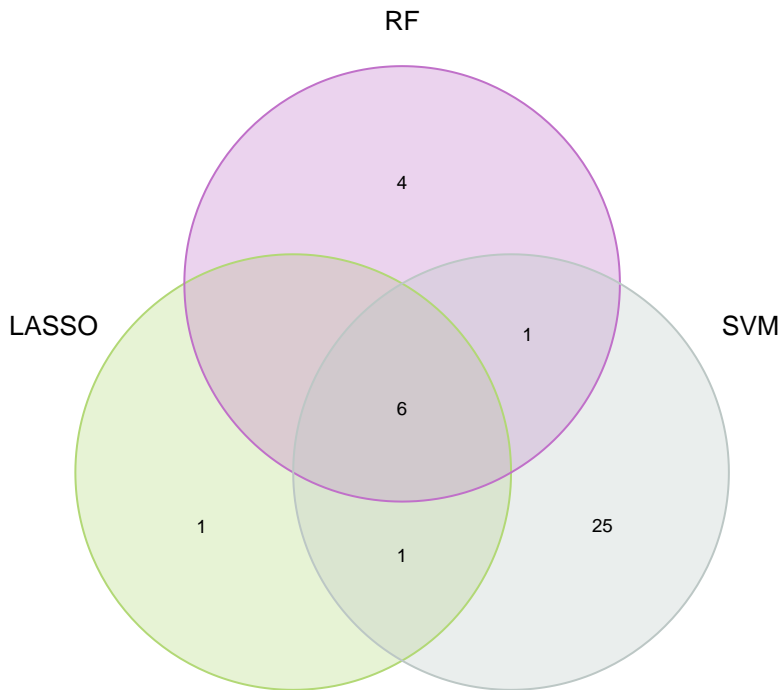

Supplement: Supplementary file 3 [file DataSheet_3.zip › 3.machine learning/4.venn/OFGs.pdf]

**KLB**

Sensitivity

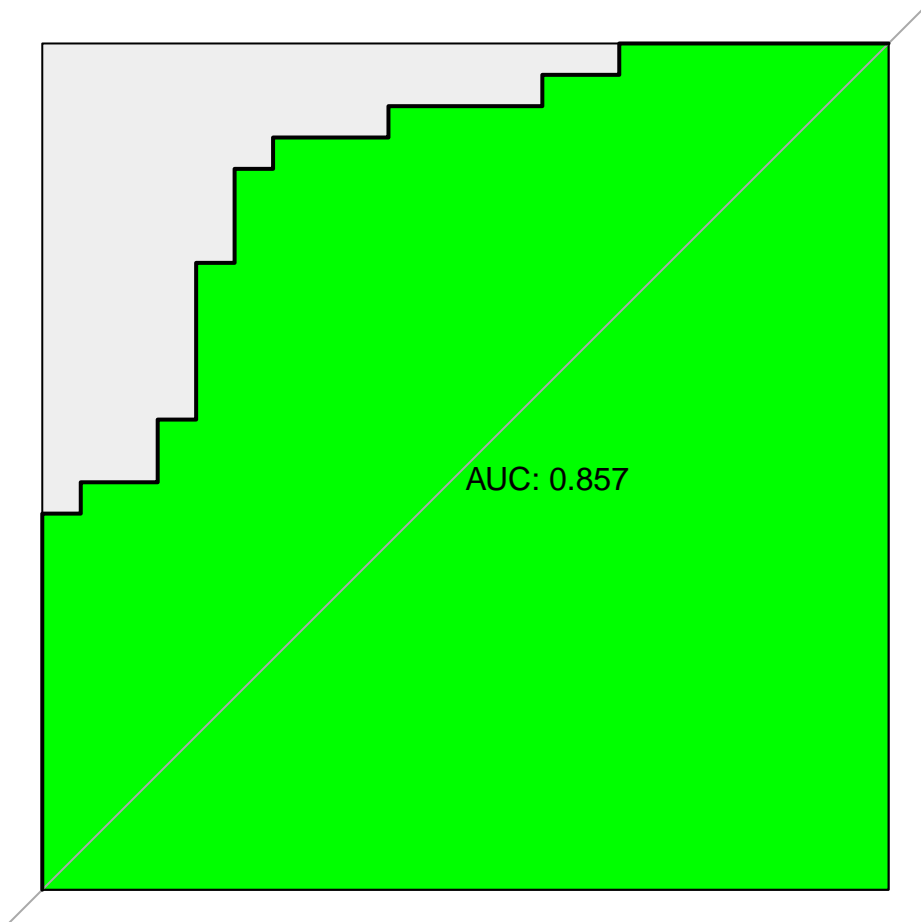

AUC: 0.857

Specificity

Supplement: Supplementary file 4 [file DataSheet_4.zip › 4.ROC+nomogram/KLB_P.pdf]

**LINC01140**

Sensitivity

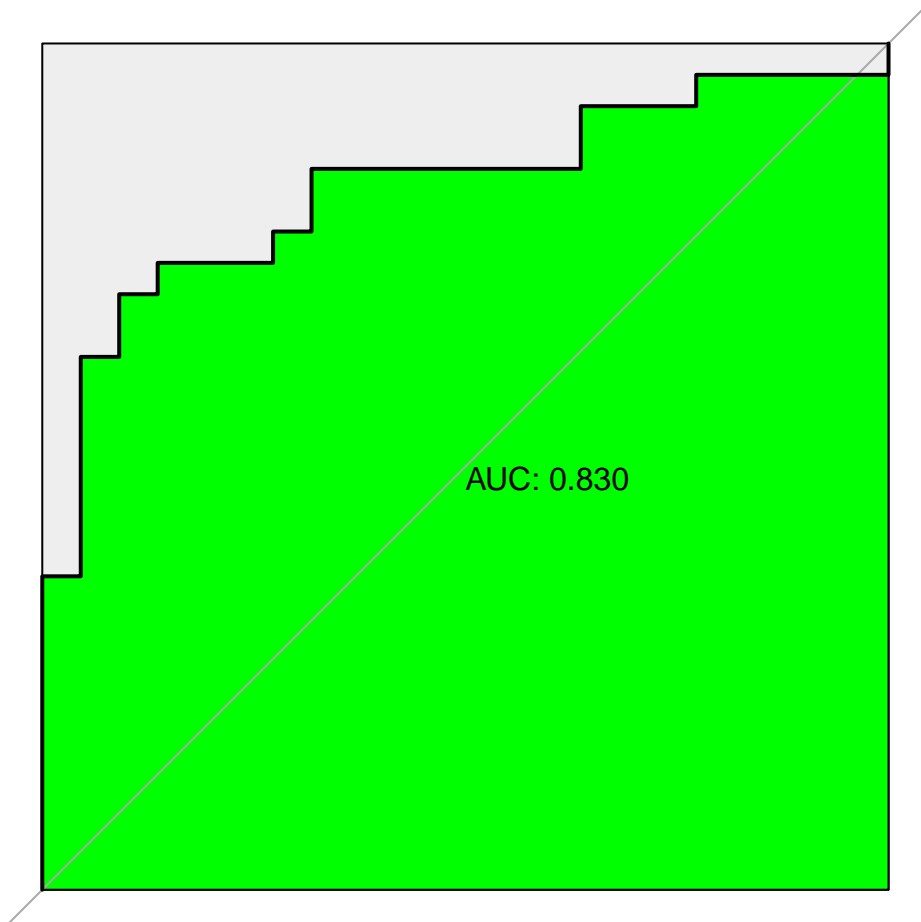

AUC: 0.830

Specificity

Supplement: Supplementary file 4 [file DataSheet_4.zip › 4.ROC+nomogram/LINC01140_P.pdf]

**LINC01697**

Sensitivity

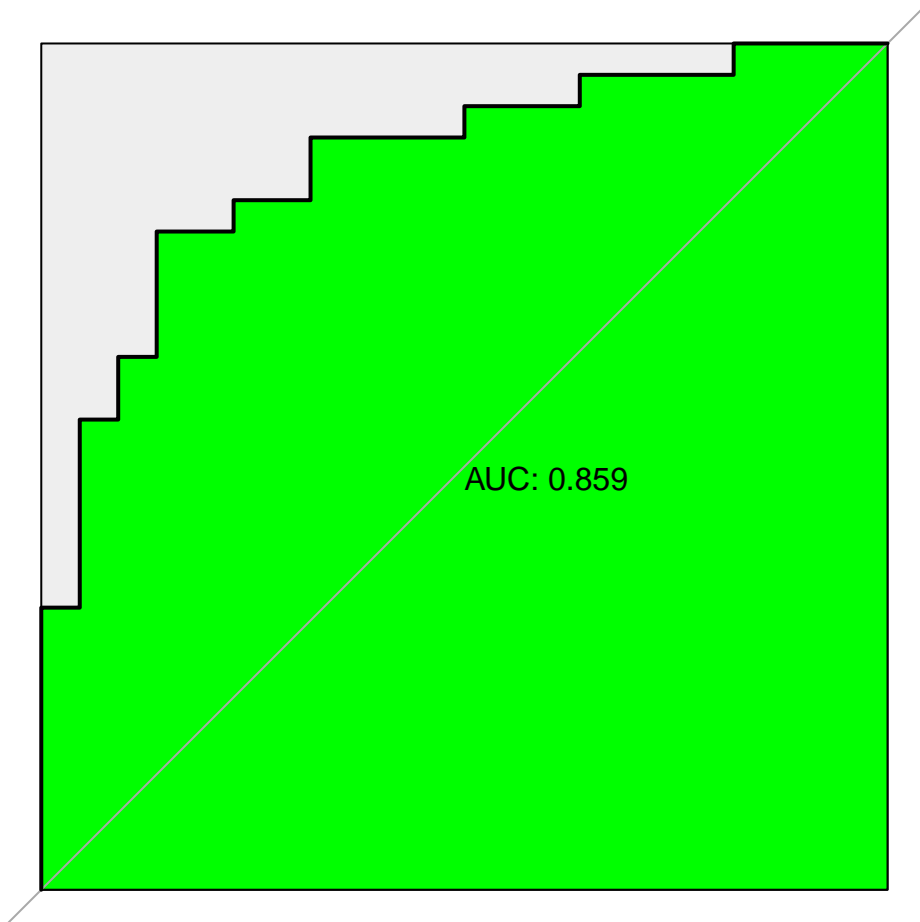

AUC: 0.859

Specificity

Supplement: Supplementary file 4 [file DataSheet_4.zip › 4.ROC+nomogram/LINC01697_P.pdf]

**SGCG**

Sensitivity

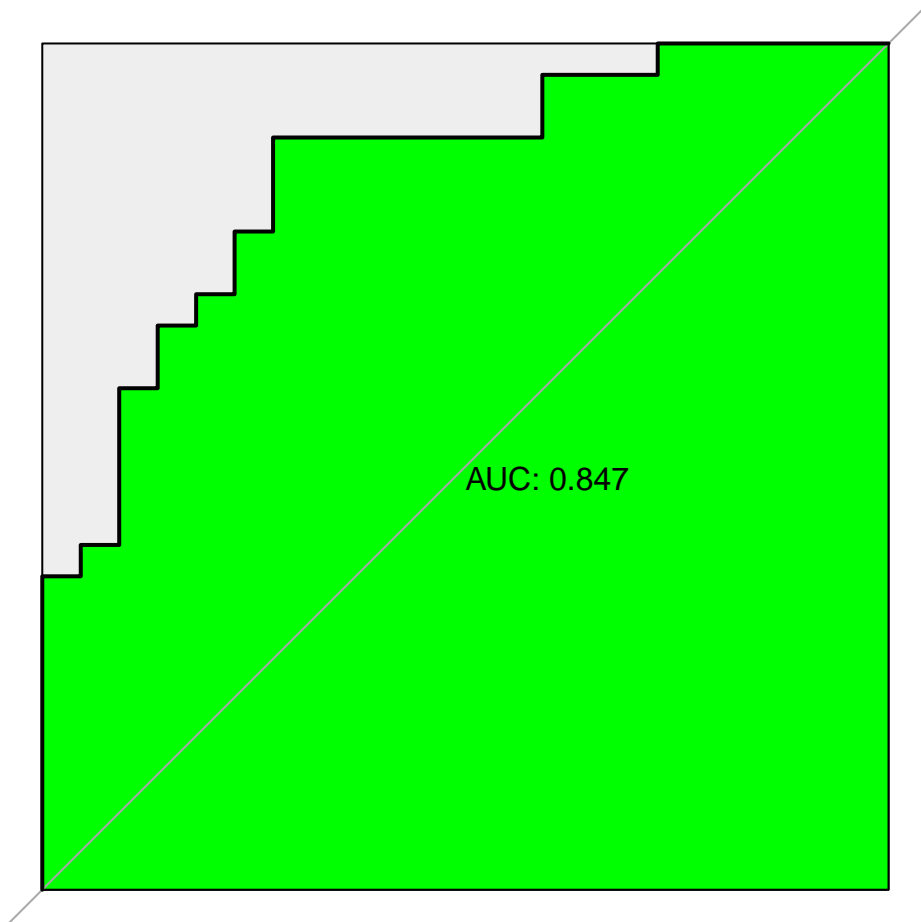

AUC: 0.847

Specificity

Supplement: Supplementary file 4 [file DataSheet_4.zip › 4.ROC+nomogram/SGCG_P.pdf]

# TBC1D2B

Sensitivity

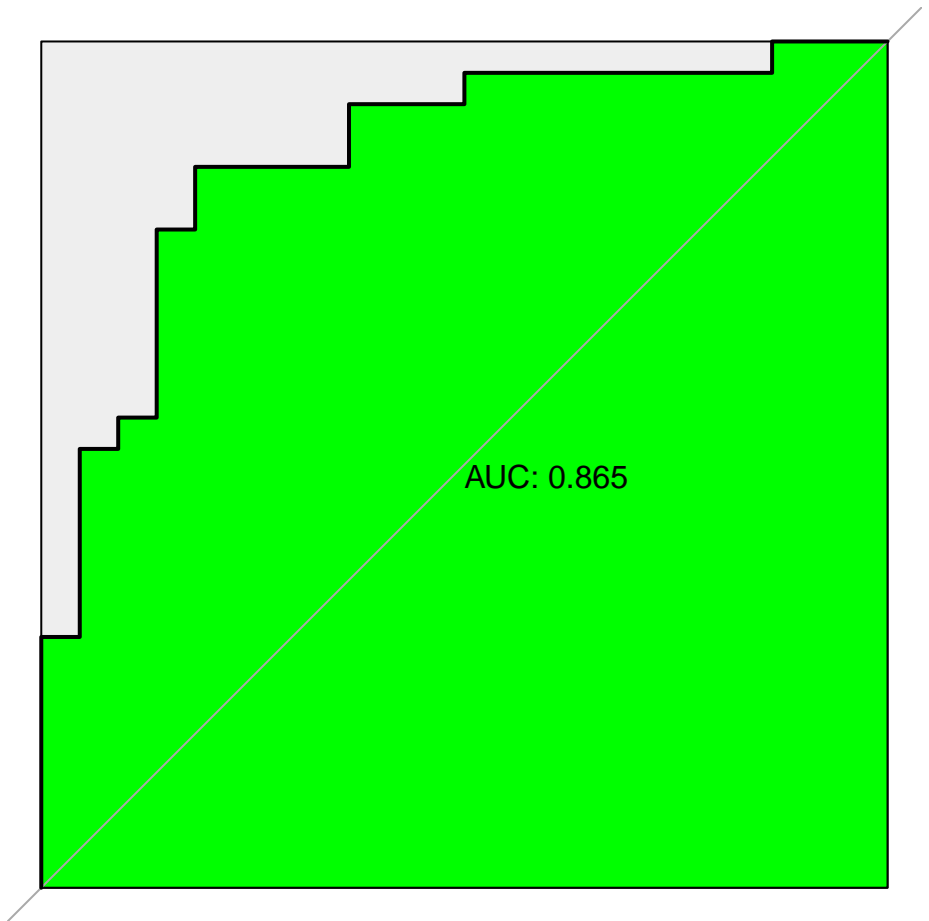

AUC: 0.865

Specificity

Supplement: Supplementary file 4 [file DataSheet_4.zip › 4.ROC+nomogram/TBC1D2B_P.pdf]

**TMEM37**

Sensitivity

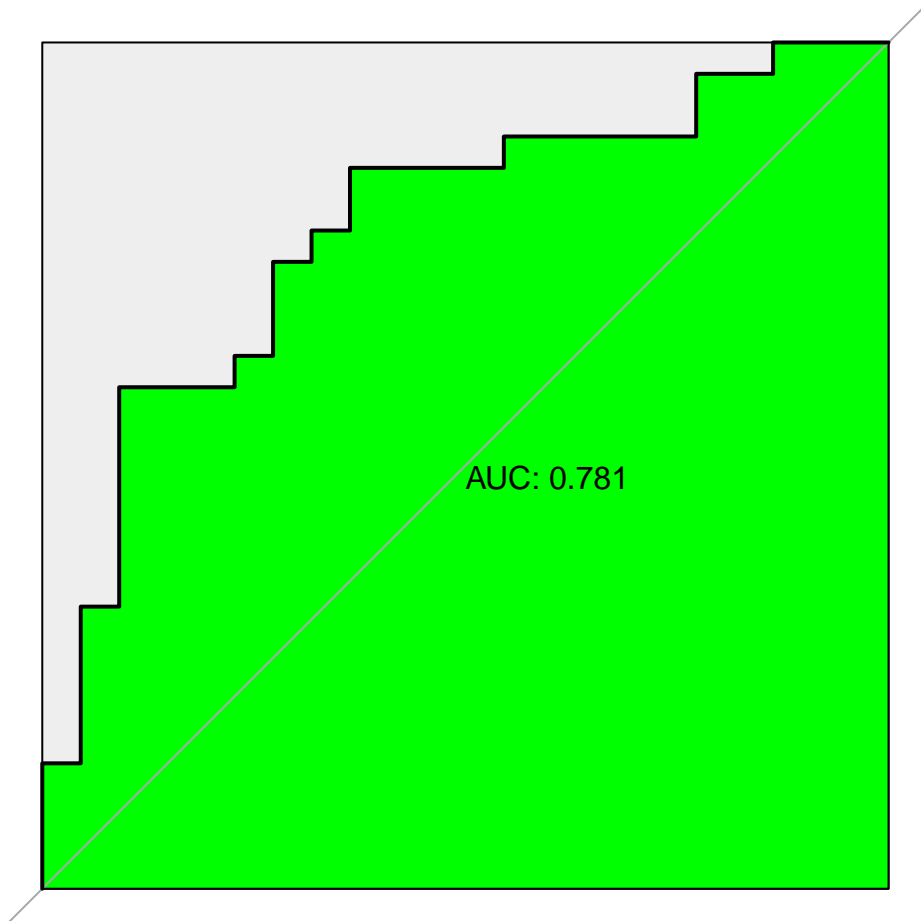

AUC: 0.781

Specificity

Supplement: Supplementary file 4 [file DataSheet_4.zip › 4.ROC+nomogram/TMEM37_P.pdf]

Points

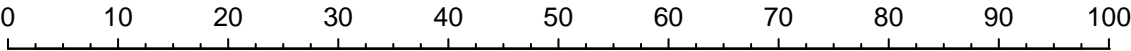

KLB

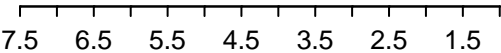

TBC1D2B

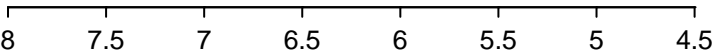

LINC01140

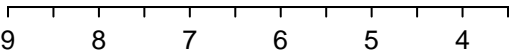

SGCG

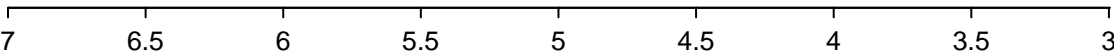

TMEM37

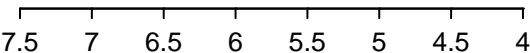

LINC01697

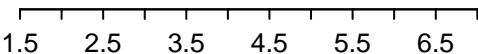

Total Points

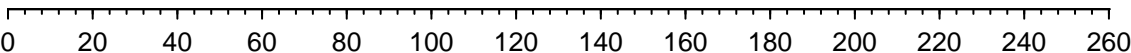

Linear Predictor

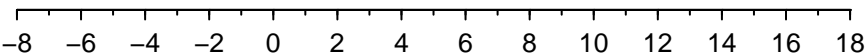

Supplement: Supplementary file 4 [file DataSheet_4.zip › 4.ROC+nomogram/nomogram.pdf]

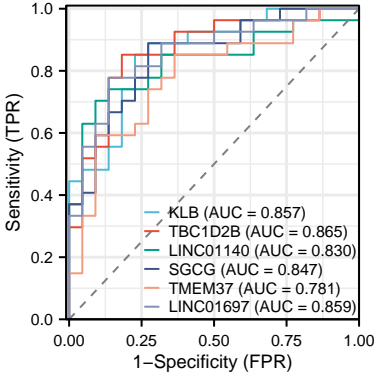

Supplement: Supplementary file 4 [file DataSheet_4.zip › 4.ROC+nomogram/诊断ROC.pdf]

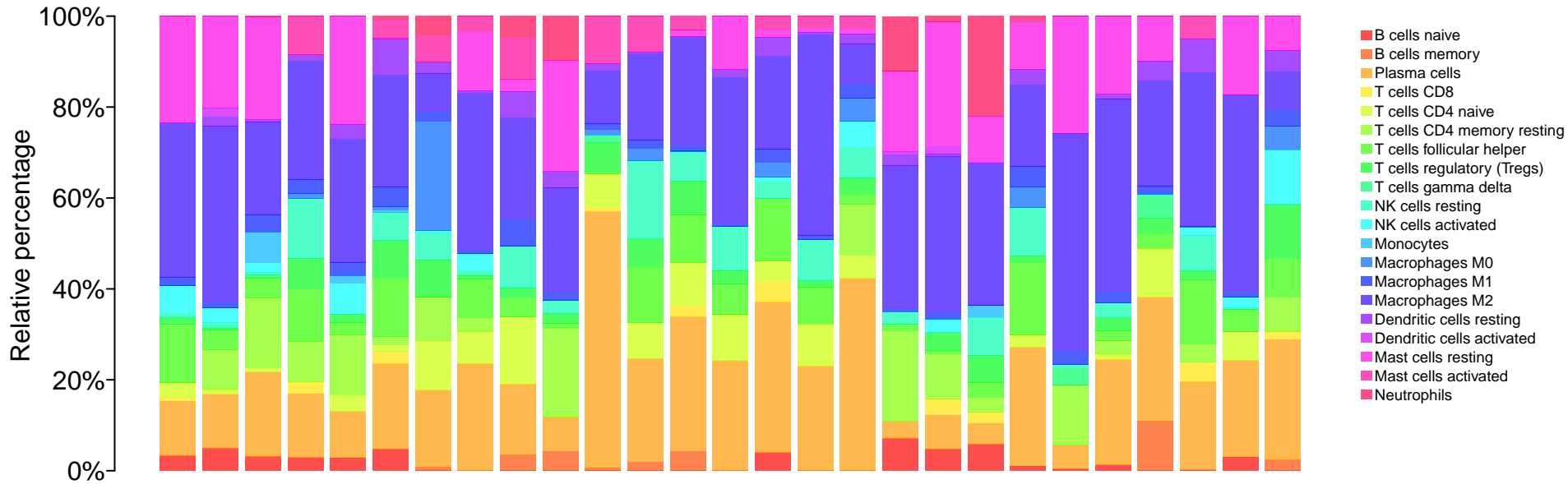

Supplement: Supplementary file 5 [file DataSheet_5.zip › 5.cibersort/Rplot.pdf]

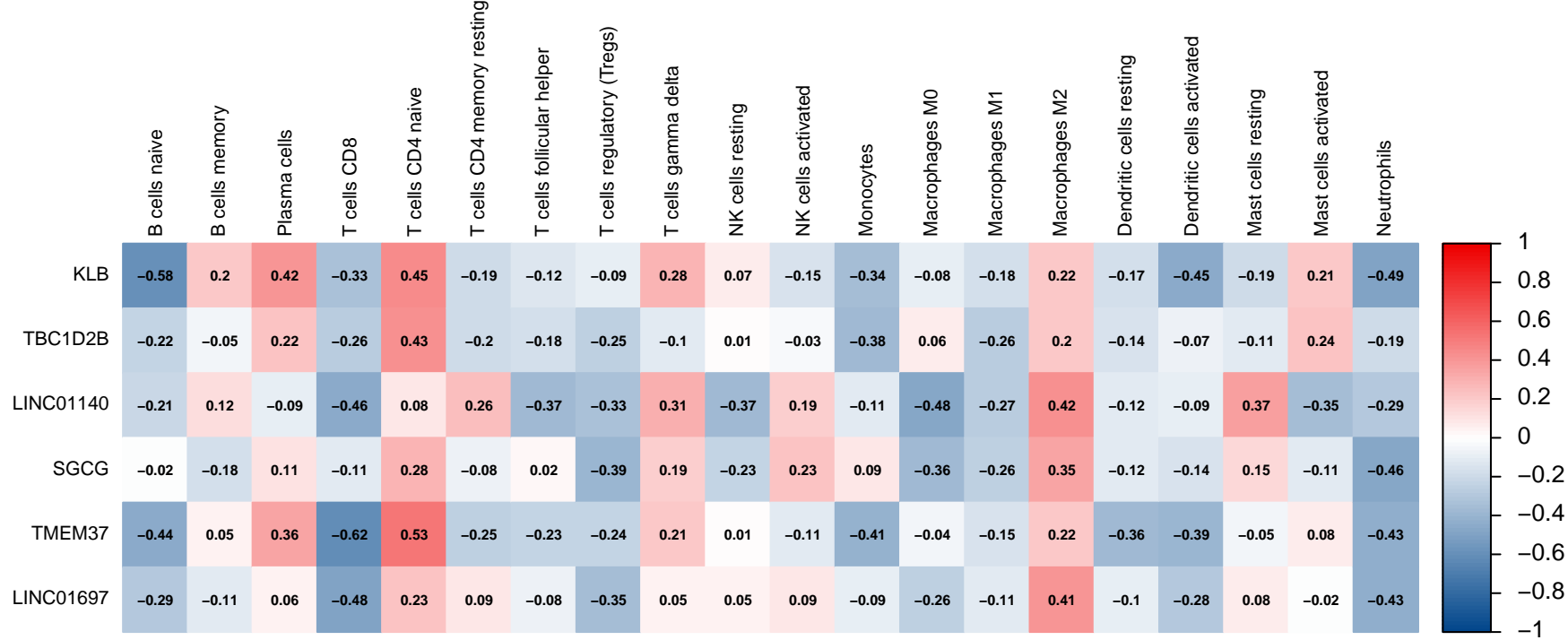

Supplement: Supplementary file 5 [file DataSheet_5.zip › 5.cibersort/Rplot01.pdf]

Proportion

group Normal TAO

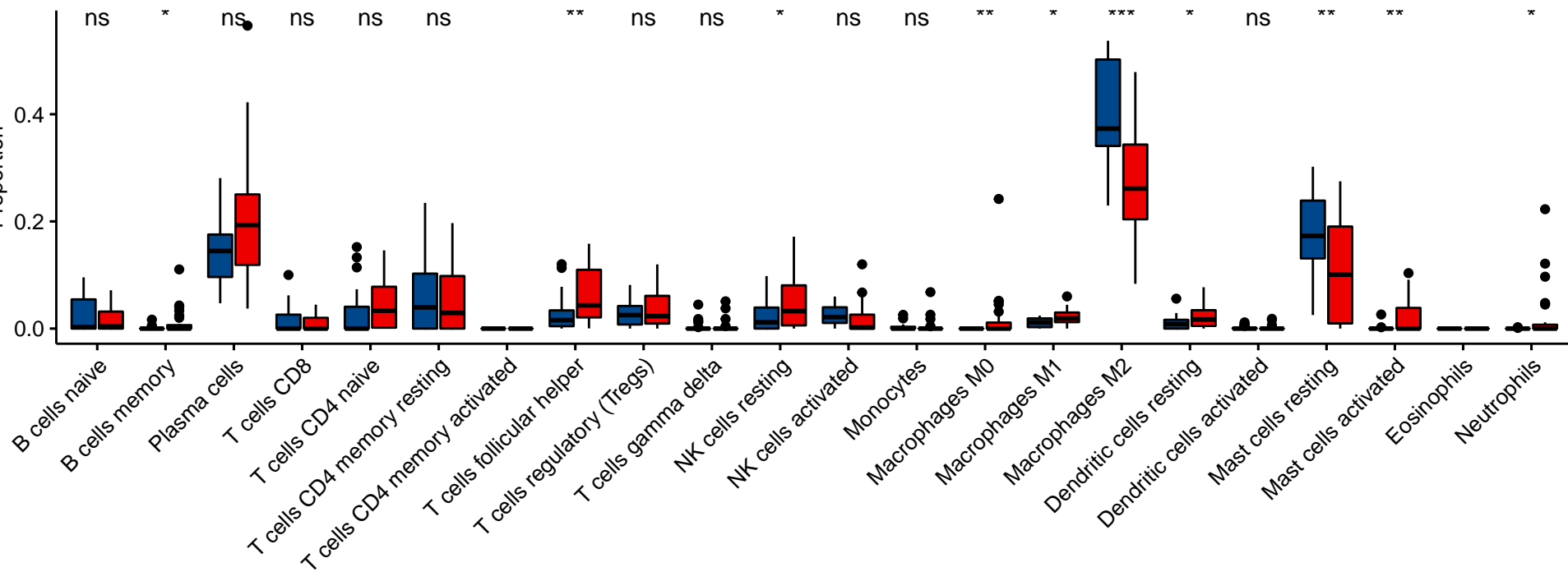

CIBERSORT

Supplement: Supplementary file 5 [file DataSheet_5.zip › 5.cibersort/Rplot02.pdf]

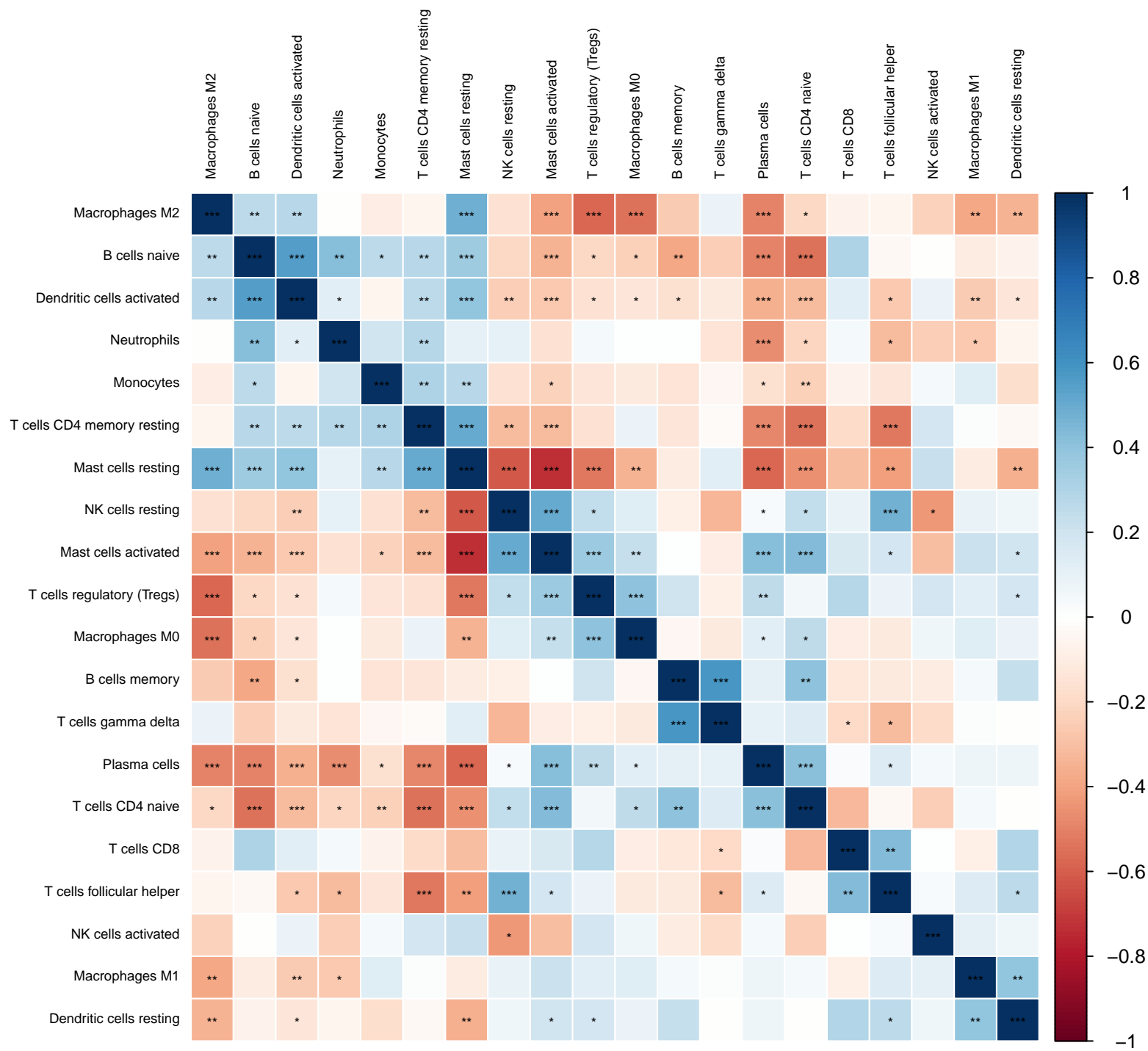

Supplement: Supplementary file 5 [file DataSheet_5.zip › 5.cibersort/Rplot03.pdf]

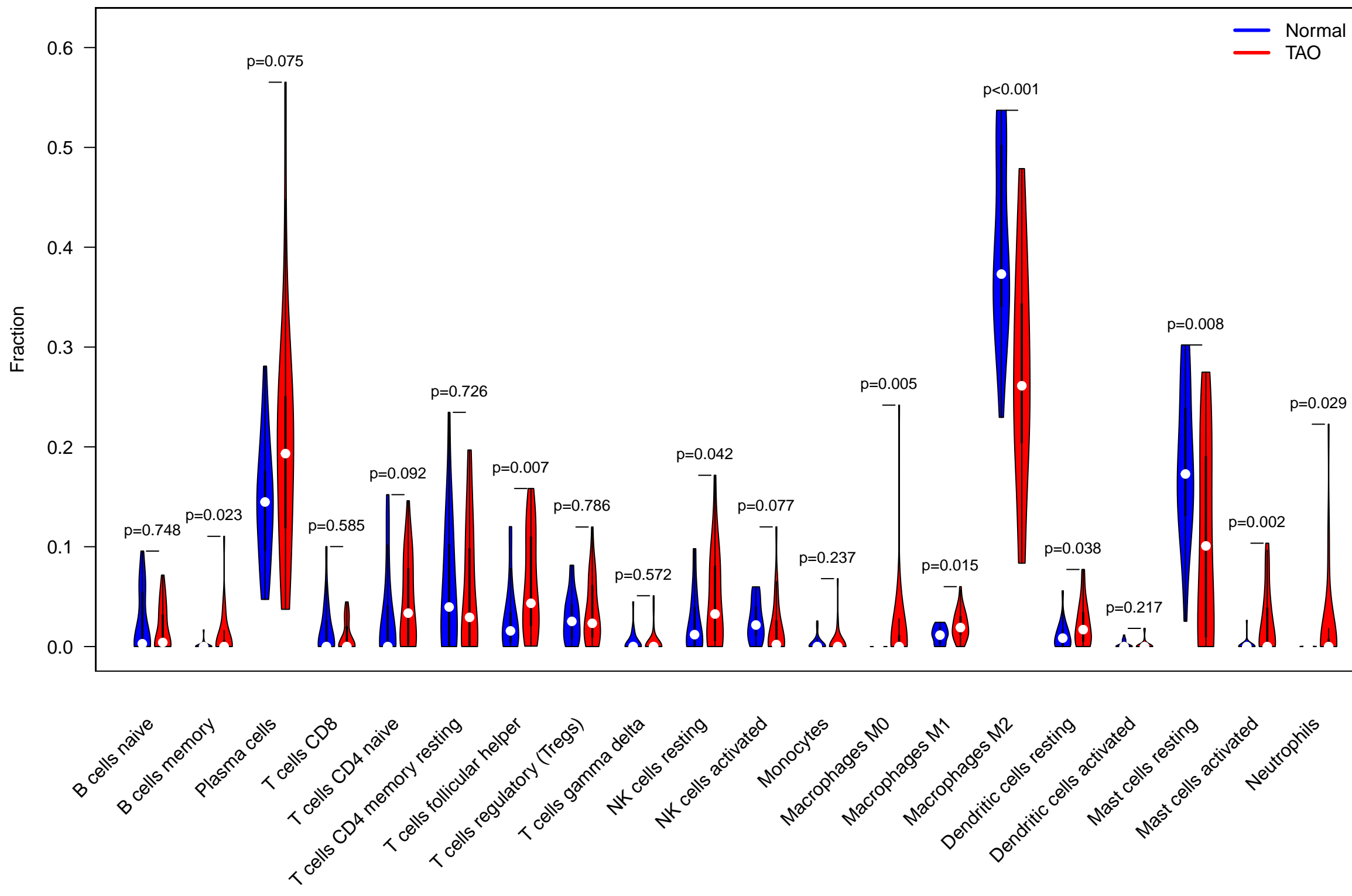

Supplement: Supplementary file 5 [file DataSheet_5.zip › 5.cibersort/vioplot.pdf]

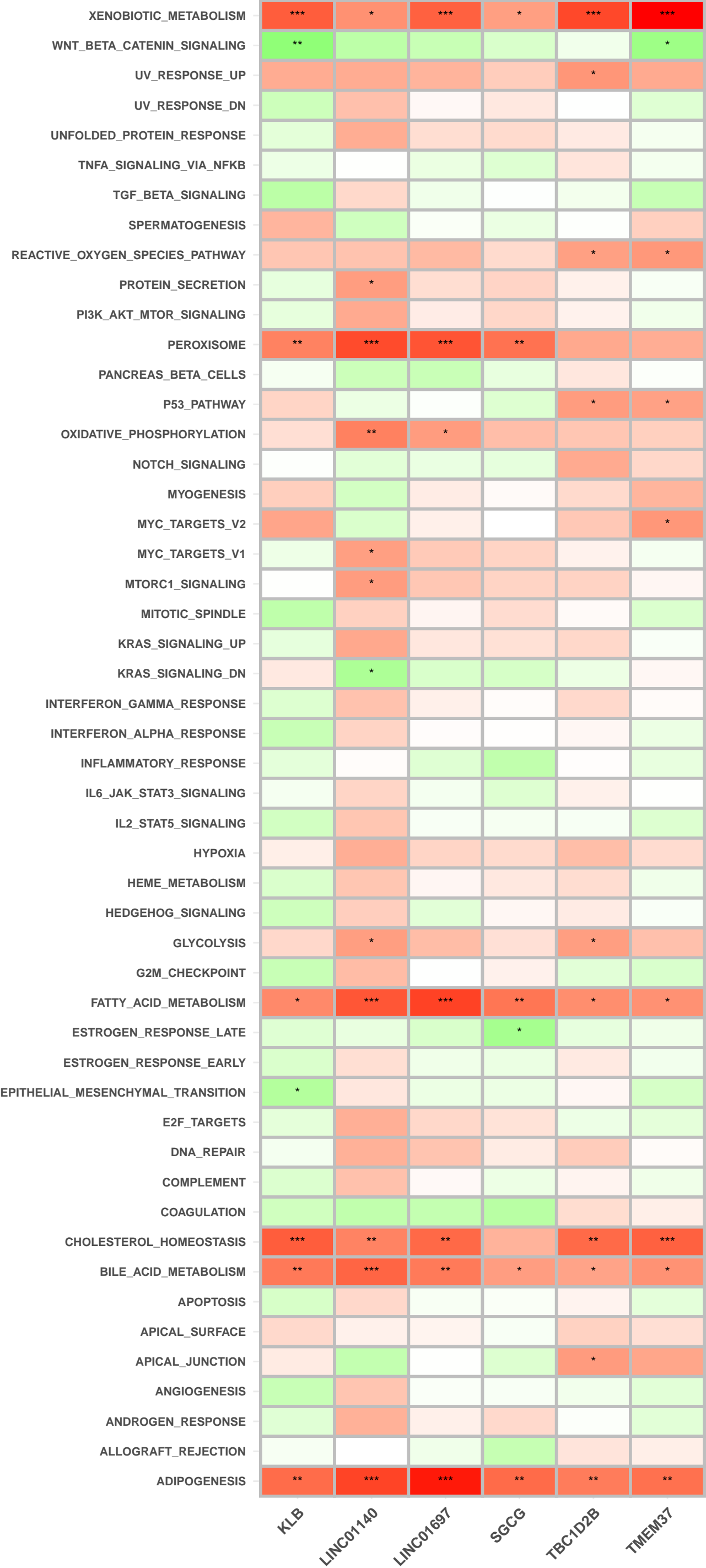

\*\*\* p<0.001  
\*\* p<0.01  
\* p<0.05

Correlation

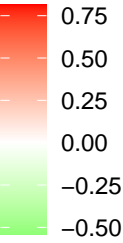

Supplement: Supplementary file 6 [file DataSheet_6.zip › 6.ssGSEA/cor.pdf]
